# Tipping Points in Bacterial Richness Amplify Extracellular Enzyme Activities in Northern Peatlands

**DOI:** 10.1111/gcb.71099

**Published:** 2026-09-17

**Authors:** Vincent E. J. Jassey, Tristan Lafont Rapnouil, Marie Le Geay, Romain Walcker, Laure Gandois, Maialen Barret, Nicolas Fanin, Bjorn J. M. Robroek, Béatrice Lauga, Luke O. Andrews, Alix Badré Greuza, Mark R. Bakker, Guillaume Bertrand, Tobias Birkwald, Paulo A. V. Borges, Brian Branfireun, Francis Q. Brearley, Cécile Brousseau, Zhao‐Jun Bu, Jean‐François Carrias, Sarah Coffinet, Bruno Corbarra, Eduardo M. F. Dias, Ellen Dorrepaal, André‐Jean Francez, Yuwen Fu, Marvin Gabriel, Rosalina Gabriel, Stéphanie Gerin, Daniel Gilbert, Sébastien Gogo, Gustaf Granath, Emma Gray, Liam Heffernan, Thierry J. Heger, Sari Juutinen, Paul Kardol, Edgar Karofeld, Agata Klimkowska, Martin Küttim, Liisa Küttim, Olivia Kuuri‐Riutta, Terri Lacourse, Mariusz Lamentowicz, Gaël Le Roux, Zoë Lindo, Roy Mackenzie, Katarzyna Marcisz, Yuri A. Mazei, Natalia G. Mazei, Cândida M. F. Mendes, Edward A. D. Mitchell, Juanita Mora‐Gomez, Raphael Müller, Kathy Pouliot, Anna‐Helena Purre, Achim Quaiser, Brenda Riquelme‐del Río, Allison R. Rober, Line Rochefort, Ana C. Rodríguez, A. Britta K. Sannel, Fabian Seemann, Laurent Servière, Maria Strack, Graeme T. Swindles, Julie Talbot, Andrey N. Tsyganov, Eeva‐Stiina Tuittila, Alexander T. Tveit, Minna M. Väliranta, Kevin H. Wyatt, Henni Ylänne, Zicheng Yu, Evgeny A. Zarov, Samuel Hamard

**Affiliations:** ^1^ Université de Toulouse, Toulouse INP, CNRS, IRD, CRBE Toulouse France; ^2^ Université d'Angers, Institut Agro, INRAE, IRHS, SFR QUASAV Angers France; ^3^ INRAE, Bordeaux Sciences Agro, UMR 1391 ISPA Villenave‐d'Ornon France; ^4^ Department of Ecology, Radboud Institute for Biological and Environmental Sciences Radboud University Nijmegen the Netherlands; ^5^ Universite de Pau et des Pays de l'Adour, IPREM Pau France; ^6^ School of Biological and Environmental Sciences Liverpool John Moores University Liverpool UK; ^7^ Department of Geography and Planning University of Liverpool Liverpool UK; ^8^ PNR des ballons des Vosges Munster France; ^9^ Université Marie et Louis Pasteur, CNRS, Chrono‐Environnement (UMR 6249) Besançon France; ^10^ UNESCO‐Biosphärenreservat Rhön, Bayerische Verwaltung Oberelsbach Germany; ^11^ CE3C‐Centre for Ecology, Evolution and Environmental Changes, Azorean Biodiversity Group, CHANGE‐Global Change and Sustainability Institute. School of Agricultural and Environmental Sciences University of the Azores Angra do Heroísmo Azores Portugal; ^12^ Department of Biology Western University London Ontario Canada; ^13^ Department of Natural Sciences Manchester Metropolitan University Manchester UK; ^14^ ANA Conservatoire d'Espaces Naturels Ariège Cos France; ^15^ Key Laboratory of Geographical Processes and Ecological Security in Changbai Mountains, Ministry of Education, School of Geo‐Graphical Sciences Northeast Normal University Changchun China; ^16^ Laboratoire Microorganismes: Génome et Environnement (LMGE) CNRS, Université Clermont Auvergne Clermont‐Ferrand France; ^17^ Université de Rennes, CNRS, UMR ECOBIO Rennes France; ^18^ IITAA, Azores University Açores Portugal; ^19^ Climate Impacts Research Centre, Department of Ecology and Environmental Science Umeå University Abisko Sweden; ^20^ Northeast Institute of Geography and Agroecology Chinese Academy of Sciences Changchun China; ^21^ Team Ecological Restoration Nature and Biodiversity Conservation Union (NABU) Berlin Germany; ^22^ Climate System Research Finnish Meteorological Institute Helsinki Finland; ^23^ Department of Ecology and Genetics EBC Uppsala Sweden; ^24^ Atlantic Technological University, ATU Galway City Galway Ireland; ^25^ Earth Sciences Vrije Universiteit Amsterdam Amsterdam the Netherlands; ^26^ Changins, HES‐SO University of Applied Sciences and Arts Western Nyon Switzerland; ^27^ Department of Forest Mycology and Plant Pathology Swedish University of Agricultural Sciences Uppsala Sweden; ^28^ Department of Forest Ecology and Management Swedish University of Agricultural Sciences Umeå Sweden; ^29^ Institute of Ecology and Earth Sciences University of Tartu Tartu Estonia; ^30^ Department of Biology, Geobiology Research Group University of Antwerp Wilrijk Belgium; ^31^ Wetlands International Wageningen the Netherlands; ^32^ Institute of Ecology Tallinn University Tallinn Estonia; ^33^ School of Forest Sciences University of Eastern Finland Joensuu Finland; ^34^ Department of Biology University of Victoria Victoria British Columbia Canada; ^35^ Climate Change Ecology Research Unit, Faculty of Geographical and Geological Sciences Adam Mickiewicz University Poznan Poland; ^36^ Instituto de Ciencias Aplicadas Universidad Autónoma de Chile. Avenida del Valle 534 Santiago Chile; ^37^ Millenium Institute Biodiversity of Antarctic and Subantarctic Ecosystems (BASE) Santiago Chile; ^38^ Cape Horn International Center (CHIC) Universidad de Magallanes Puerto Williams Chile; ^39^ Lomonosov Moscow State University Moscow Russia; ^40^ Shenzhen MSU‐BIT University Shenzhen China; ^41^ Laboratory of Soil Biodiversity University of Neuchâtel Neuchâtel Switzerland; ^42^ Institut des Sciences de la Terre d'Orléans (ISTO) UMR 7327, Univ. Orléans, CNRS, BRGM, OSUC Orléans France; ^43^ Faculty of Earth Sciences, Geography and Astronomy, Department of Geography and Regional Research, Geoecology University of Vienna Vienna Austria; ^44^ Department of Plant Science, Peatland Ecology Research Group and Centre for Northern Studies Université Laval Quebec Quebec Canada; ^45^ Elige OÜ Tallinn Estonia; ^46^ School of Environment and Natural Resources The Ohio State University Columbus Ohio USA; ^47^ Fundación Centro de los Bosques Nativos FORECOS Valdivia Chile; ^48^ Department of Physical Geography Stockholm University Stockholm Sweden; ^49^ Permafrost Research Section Alfred Wegener Institute Helmholtz Centre for Polar and Marine Research Potsdam Germany; ^50^ Ana‐Conservatoire d'espaces Naturels Ariège Alzen France; ^51^ Department of Geography and Environmental Management University of Waterloo Waterloo Canada; ^52^ Geography, School of Natural and Built Environment Queen's University Belfast Belfast UK; ^53^ Groupe de Recherche Interuniversitaire en Limnologie (GRIL) Montréal Canada; ^54^ Département de Géographie Université de Montréal Montréal Quebec Canada; ^55^ A.N. Severtsov Institute of Ecology and Evolution Russian Academy of Sciences Moscow Russia; ^56^ Department of Arctic and Marine Biology, Faculty of Biosciences, Fisheries and Economics UiT—The Arctic University of Norway Tromsø Norway; ^57^ Department of Environmental Sciences, Environmental Change Research Unit University of Helsinki Helsinki Finland; ^58^ Yugra State University, UNESCO Chair “Environmental Dynamic and Global Climate Changes” Khanty‐Mansiysk Russian Federation

## Abstract

Bacterial communities are vital to northern peatlands' carbon and nutrient cycling, one of the Earth's largest terrestrial carbon stores. However, their diversity and ecological roles at broad geographic scales remain partially understood, limiting our ability to predict their response to global change. Here, we combine a trans‐Holarctic survey across 171 *Sphagnum*‐dominated peatlands (SDPs) with a continental‐scale reciprocal transplantation experiment to quantify how bacterial diversity shapes carbon and nutrient cycling across bioclimatic gradients. We show that bacterial diversity and composition differ markedly among peatland bioclimatic regions and are primarily structured by the universal abiotic drivers of northern ecosystems: minimum temperature, snow cover, and soil water content. Ecological models further revealed that deterministic processes accounted for approximately 80% of bacterial community assembly, highlighting the strong influence of environmental filtering. Biodiversity‐Ecosystem Function analyses identified bacterial richness as a key driver of carbon and nutrient cycling. Using moving‐window structural equation models, we identified a critical bacterial richness threshold, below which extracellular enzyme activities increased by ~60%. This transition coincided with stronger environmental filtering and shifts towards bacterial communities with a greater predicted potential for extracellular nutrient acquisition and heterotrophic metabolism. Together, these findings demonstrate that bacterial richness underpins peatland biogeochemical functioning and suggest that biodiversity loss may accelerate carbon turnover, weakening the capacity of northern peatlands to retain carbon. As climate change threatens this unique microbiome, safeguarding bacterial diversity will be critical for maintaining ecosystem resilience and the stability of this globally important carbon sink.

## Graphical abstract

Using a trans‐Holarctic survey in *Sphagnum*‐dominated peatlands, we identify a critical bacterial biodiversity threshold. Below this threshold, ecosystem function shifts, increasing enzyme activities by 60%, potentially accelerating carbon turnover. This weakens the peatland carbon sink, emphasizing the need to safeguard diversity.

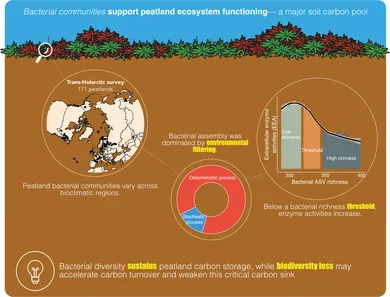

## 1 Introduction

Peatlands are among the most vital and globally significant soil carbon (C) sequestration hotspots, providing critical climate‐regulating services (Strack et al. 2022). These ecosystems, characterised by slow decomposition and the accumulation of organic matter over time, store vast quantities of soil C, estimated at 500–1000 Gt C globally (Nichols and Peteet 2019). As persistent sinks of atmospheric carbon dioxide (CO_2_), and despite C losses as methane and dissolved organic C (DOC), peatlands have effectively cooled the global climate for centuries (Frolking and Roulet 2007). However, these ecosystems are increasingly vulnerable to global change (Dise 2009). Once widespread, peatlands are now primarily confined to the Holarctic region, which holds nearly 85% of the global peatland C stock (Hugelius et al. 2020).

The size of the peatland organic C pool strongly relies on the activity of microorganisms (Fenner and Freeman 2011). With billions of cells per gram of peat (Gilbert and Mitchell 2006), microorganisms dominate life in northern peatlands and drive C dynamics, including the decomposition of organic matter, C mineralisation, and greenhouse gas fluxes (Andersen et al. 2013; Gios et al. 2024; Hamard et al. 2025). Consequently, shifts in microbial community composition can profoundly affect these biogeochemical processes (Hamard et al. 2025; Jassey et al. 2018). Despite its central role in peatland C cycling, microbial life remains largely unexplored across broad spatial scales. Most studies focus on local and regional patterns (e.g., Bragina et al. 2014; Kolton et al. 2022; Peltoniemi et al. 2015; Robroek et al. 2021), leaving the Holarctic peatland microbiome unresolved. In particular, the ecological processes that drive large‐scale variation in peatland microbial diversity, community composition and abundance, and their implications for peatland functioning, remain poorly understood. This knowledge gap hinders our ability to predict how environmental changes will alter microorganism‐mediated processes that regulate peatland C cycling on a global scale.

Understanding the processes that govern microbial community assembly and biogeography is challenging. Microbial communities are not random assemblages but comprise taxa with diverse ecological strategies, environmental tolerances and dispersal capacities. According to the niche theory, deterministic processes, such as environmental selection imposed by abiotic conditions (e.g., pH, nutrient availability) and biotic interactions (e.g., competition), primarily structure microbial communities (Riddley et al. 2025; Zhou and Ning 2017). Depending on environmental heterogeneity, deterministic selection can drive communities towards either divergent (heterogeneous selection) or convergent (homogeneous selection) compositions. For example, in peatlands, microbial communities occupying similar environmental niches can converge, even in distant locations (Hamard et al. 2021; Kolton et al. 2022). In contrast, the neutral theory emphasises stochastic processes, such as dispersal limitation, ecological drift and homogenising dispersal. These processes can maintain differences among communities regardless of contemporary environmental conditions (Caruso et al. 2011; Zhou and Ning 2017). Despite their potential importance, the relative contributions of assembly processes in northern peatlands are largely unknown. Given the strong environmental constraints imposed by the acidic, nutrient‐poor, and waterlogged conditions of peatlands, we hypothesise that deterministic environmental selection dominates microbial community assembly. This would result in predictable shifts in microbial diversity and community composition along environmental gradients. Conversely, a greater contribution from stochastic processes would suggest that historical and dispersal signatures outweigh environmental filtering, resulting in less predictable biodiversity patterns across northern peatlands.

In addition to identifying which processes determine community composition, an important unresolved question is whether assembly‐driven shifts in microbial community composition translate into changes in ecosystem functioning. Large‐scale studies have shown that peatland plant communities can maintain ecosystem resilience through the turnover of functionally redundant species (Robroek et al. 2017). Similarly, microorganisms exhibit substantial functional overlap despite their high taxonomic diversity (Louca et al. 2016, 2018). However, functional redundancy is unlikely to be unlimited (White et al. 2023). While many microorganisms share broad metabolic capabilities, they often differ in terms of enzyme efficiencies, growth rates, and biomass allocation under environmental gradients (Galen 2025; Maynard et al. 2019). As microbial diversity declines, the pool of functionally similar species may shrink, reducing the capacity of communities to buffer ecosystem functioning (Mori et al. 2018). Beyond a critical biodiversity threshold, even small changes in microbial diversity may trigger disproportionately large changes in ecosystem processes. Therefore, we hypothesise that peatland functioning remains relatively stable across moderate levels of microbial community turnover due to functional redundancy among species. However, once microbial diversity declines below a critical threshold, peatland functioning will change markedly. Establishing whether such a threshold exists is crucial for forecasting the stability of peatland C cycling in the context of ongoing climate change.

**FIGURE 1:**
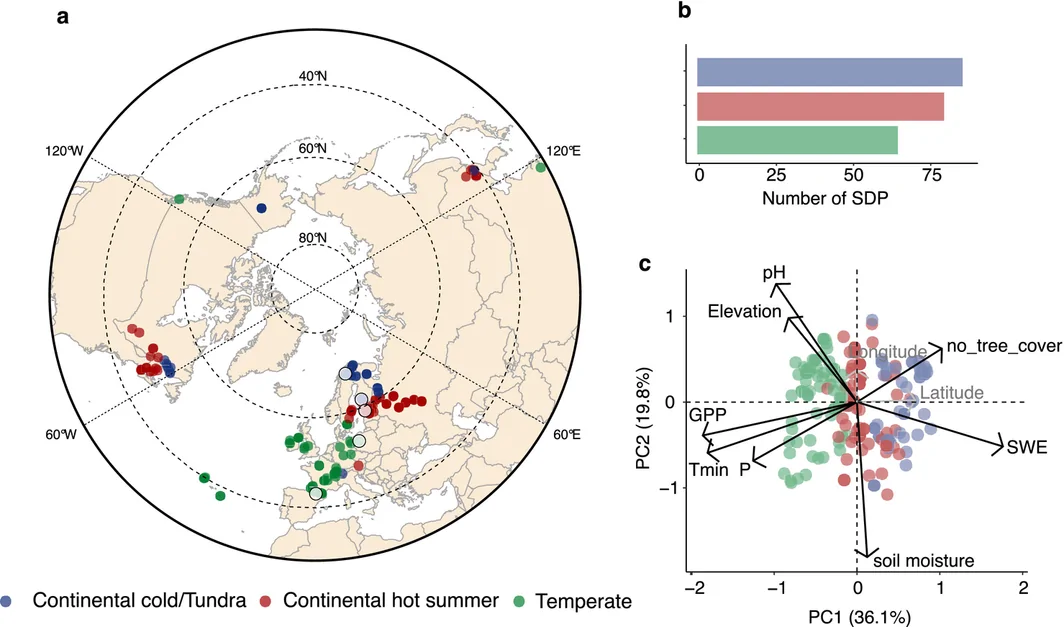
Sampled *Sphagnum*‐dominated peatlands (SDPs) and their environmental characteristics. (a) Circumpolar map showing the location of each sampled SDP. Dots colours depict the different bioclimatic regions sampled, according to the Köppen climate classification (see Methods for details). Pale white dots indicate sites where the reciprocal transplant experiment was conducted (Hamard et al. 2025). (b) Number of SDP sampled in each bioclimatic area. (c) Principal component analysis depicting the main environmental gradients characterising SDPs. Variables were selected among a stack of 178 remote sensing variables to reduce collinearity and represent the gradients and types of variables best (see Methods). Latitude and longitude were projected onto the PCA space a posteriori for visualisation purposes and were not included in the PCA construction. GPP, gross primary productivity; Tmin, minimum temperature; SWE, snow water equivalent; P, precipitation, no_tree_cover: percentage of non‐tree cover.

Herein, we investigated large‐scale variation in bacterial communities living on the surface (0–5 cm) of *Sphagnum*‐dominated peatlands (hereafter, SDPs). Across three bioclimatic regions (Continental cold/Tundra, Continental hot summer and Temperate; Figure 1a,b), each characterised by distinct climate and soil properties (Figure 1c), we sampled dominant *Sphagnum* patches in 171 SDPs. Leveraging 15,218 bacterial amplicon sequence variants (ASVs) for an average of 66.2 million bacterial cells per gram of dry moss, we characterised continental‐scale patterns in peatland bacterial diversity and cell density and identified the environmental drivers underlying these patterns. To assess the roles of deterministic and stochastic processes in bacterial community assembly in SDPs, we combined phylogenetic approaches with a continental reciprocal transplant experiment (Figure 1c). Finally, we linked bacterial diversity, community composition and cell density to biogeochemical functioning, as indicated by enzyme activities, to determine whether changes in bacterial communities translate into proportional changes in ecosystem functioning or whether critical thresholds in bacterial diversity mark abrupt shifts in this relationship. Together, these analyses establish a comprehensive framework for understanding how peatland bacterial diversity shapes peatland functioning across continental scales.

## 2 Methods

### 2.1 Trans‐Holarctic Sample Collection

*Sphagnum* samples were collected from 171 SDPs spanning the Azores, the British Isles, the Pyrenees, the European Alps and the Jura Mountains, Fennoscandia, Estonia, eastern and western Canada, Alaska, China, and western Russia, between May and September 2021 (Table S1). Sampling was mainly conducted in the summer during the growing season. At each SDP, we followed a standardised protocol to sample the dominant *Sphagnum*‐dominated microhabitat (hummocks, hollows, or lawns) with minimal vascular plant cover, allowing us to prioritise a broad spatial coverage across sites rather than intensive replication within sites. This choice was supported by preliminary analyses that showed that variability between sites exceeded variability within sites (Figure S1), indicating that a single standardised sample per site captured the dominant source of variation in our system. Consequently, we focused on sampling across a larger number of sites to better cover the *Sphagnum*‐peatland distribution gradient. From each habitat, we collected *Sphagnum* shoots with sterilised tweezers from 0 to 5 cm depth near the capitula; all sampling tools were cleaned with ethanol beforehand. Immediately after collection, approximately 4 g of *Sphagnum* were cut into small pieces using scissors and divided equally into sterile 5 mL tubes. One tube contained 4 mL of DNA Lifeguard solution to preserve DNA, and another contained 4 mL of 5% Lugol solution to fix cells for subsequent flow cytometric analyses. An additional 5 g of *Sphagnum* were also collected and stored in a plastic bag for other analyses, such as water content and enzyme activities. After collection, samples were kept fresh and shipped to Toulouse (France) for DNA extraction, flow cytometry, and enzymatic analyses. Permits for sampling and export were obtained from the relevant authorities where necessary. Of these 171 samples, 115 were used for cytometry and enzymatic activity analyses due to material availability (see below).

### 2.2 Site Data

We systematically acquired a stack of 178 remote sensing‐derived variables for each of the 171 SDP locations using the Google Earth Engine Platform (Gorelick et al. 2017). All data were resampled to a uniform 250 m pixel resolution before extraction. The comprehensive variable stack (detailed in Table S3) comprised climatic variables extracted from the monthly TerraClimate dataset (Abatzoglou et al. 2018), soil variables sourced from the OpenLand (Hengl et al. 2025) and SMAP datasets (Entekhabi et al. 2016), vegetation productivity and structure metrics including vegetation indices, Leaf Area Index (LAI), Fraction of Photosynthetically Active Radiation (fPAR), Gross Primary Productivity (GPP), and Net Primary Productivity (NPP), all derived from the MODIS dataset (Zhao et al. 2005), and elevation data from the GMTED2010 dataset. For a subset of these variables (see Table S2), we extracted monthly data spanning March to October 2021, covering the sampling period. For each SDP, we used the monthly value preceding the sampling date; for example, if sampling occurred in early September, we assigned the value from the previous month, August. This monthly time series was augmented by calculating and including the mean value from January 2016 to January 2021 to capture multiannual conditions. Additionally, we retrieved bioclimatic zones for each sample from the Köppen Climate classification using the *kgc* R package (Bryant et al. 2017). Köppen codes were then aggregated into three broad regions: Temperate, Continental hot and Continental cold/Tundra (see Table S4 for details). For each site, we also requested information on soil properties, including pH and C/N ratio. While pH values were available for 40% of the sites, data on other soil properties were largely missing and excluded from analysis. Given the strong correlation between field‐measured pH values and those obtained from OpenLand (*r* = 0.49, *p* < 0.001, Figure S2), we used OpenLand pH values in our statistical analyses.

### 2.3 Reciprocal Transplant Experiment and Sampling

Five SDPs were selected to represent a broad gradient of temperature and precipitation across Europe (Figure 1): Counozouls (42°41′19.7′′ N, 2°14′02.4′′ E, France), Bagno Kusowo (53°48′47.9′′ N, 16°35′12.1′′E, Poland), Männikjärve bog (58°52′26.4″ N, 26°15′03.6″ E, Estonia), Siikaneva mire (61°50′41.6′′ N, 24°17′17.5″ E, Finland), and Stordalen (68°20′43.1″ N, 19°03′58.7″ E, Sweden). In July 2018, 125 intact *Sphagnum*‐dominated peat blocks with minimal vascular plant cover (60 × 40 × 20 cm), encased in sterilised plastic boxes, were reciprocally transplanted across the five sites. The experiment consists of five blocks per site, with each block including peat from each location (*n* = 25 per site, 125 mesocosms in total). This setup created mesocosms experiencing warmer, colder, wetter or drier climates, depending on the origin and transplant location. Untouched control plots were also established at each site (*n* = 5), one in each block at each site, to assess for potential encasing effects, which were found to be non‐significant for most monitored biotic and abiotic variables (see Hamard et al. (2025) for details). In particular, pore water chemistry analyses revealed that transplantation did not impact pH, or dissolved organic C and total nitrogen contents in mesocosms (Hamard et al. 2025; Sytiuk et al. 2023). Environmental variables, including air/soil temperature, photosynthetically active radiation (PAR) and precipitation, were continuously monitored. For further details on the experimental design and a comprehensive overview of the environmental data, see Hamard et al. (2025) and Sytiuk et al. (2023).

In July 2019, we collected *Sphagnum* shoots (0–5 cm depth) as described above. Briefly, approximately 4 g of *Sphagnum* were equally divided for DNA and flow cytometric analyses (see above for details), whilst an additional 5 g were stored in plastic bags for enzymatic analyses.

### 2.4 Bacterial Cell Density

Bacterial densities were quantified using flow cytometry. Bacterial cells were extracted from Lugol‐fixed samples following Hamard et al. (2025). Each sample was vortexed for 1 min and then manually pressed to recover the liquid fraction from the *Sphagnum* moss. From this extract, we used 1 mL aliquots to quantify the bacterial concentrations by flow cytometry. Aliquots were passed through a 40 μm filter to prevent clogging of the flow cytometer capillary, stained with SYBR Green I (0.1× final concentration), and incubated in the dark for 15 min. Samples were then analysed on a Guava easyCyte flow cytometer at a flow rate of 2 μL s^−1^ with count rates not exceeding 1000 events s^−1^. Bacterial densities were expressed as the cell numbers per gram of dry peat (ind. g^−1^ dw).

### 2.5 Bacterial Diversity and Community Composition

DNA was extracted from *Sphagnum* using the DNeasy PowerSoil Pro Kit (Qiagen) according to the manufacturer's instructions. The V3‐V4 region of the 16S rRNA gene was amplified with primers PCR1_515F (5′‐GTGYCAGCMGCCGCGGTA‐3′) and PCR1_909R (5′‐CCCCGYCAATTCMTTTRAGT‐3′) following Jassey et al. (2022), and sequenced on an Illumina MiSeq platform (250‐bp paired‐end) at the Genotoul platform (Toulouse, France). Blanks were included to monitor contamination, and all molecular work was performed in a UV‐decontaminated laboratory. Paired‐end reads were processed in QIIME2 (2020.8) and DADA2 (trimLeft = 20, trimRight = 70), yielding 19,182 ASVs after filtering for prevalence (≥ 2 SDPs) and rarefaction to 7000 reads per sample (Figure S3). Taxonomy was assigned against SILVA v138.2 (Quast et al. 2013), with uncertain assignments (< 75% bootstrap) labelled as “Unknown [level]”. A phylogenetic tree was constructed using maximum likelihood (GTR model, 1000 bootstraps). Functional potential was predicted using PICRUSt2 (Douglas et al. 2020) to infer gene family abundances, which were collapsed into Enzyme Commission (EC) numbers, retaining only those corresponding to the four quantified enzymes (Table S3). Additionally, ASVs were functionally annotated using FAPROTAX (Louca et al. 2016) via the *microeco* R package (Liu et al. 2021), and relative abundances of annotated functions were calculated, focusing on main functions related to enzyme activities. Because both PICRUSt2 and FAPROTAX infer functional potential from taxonomic composition rather than directly measuring gene content or activity, they were solely used as complementary approaches to support patterns observed in measured extracellular enzyme activities. More details can be found in Supporting Information: Methods.

### 2.6 Extracellular Enzyme Activities

The activity of four hydrolytic enzymes commonly associated with microbial metabolic rates and biogeochemical properties (Galen 2025; Sinsabaugh et al. 2008) was quantified using fluorogenic substrates labelled with 7‐amino‐4‐methylcoumarin (MUC) or methylumbelliferone (MUB; see Table S3): ß‐1,4‐glucosidase (BG; breaks down cellulose into glucose for C acquisition), ß‐1,4‐N‐acetylglucosaminidase (NAG; breaks down chitin and peptidoglycan for N and C acquisition), leucine aminopeptidase (LAP; breaks down proteins and polypeptides for N acquisition), and acid phosphatase (AP; mineralises organic phosphorus). Enzyme proteins were extracted following Criquet et al. (1999) and quantified following Jassey et al. (2018). All details are given in Supporting Information: Methods.

To ensure comparability across the trans‐Holarctic survey, enzyme activities were initially incubated at 25°C for 3 h to minimize variability from differing assay conditions (Sinsabaugh et al. 2008; Wallenstein and Weintraub 2008). However, standardized incubation may produce values differing from in situ conditions, potentially biasing observed biogeographic patterns. To address this, we also quantified enzyme activities at field‐relevant temperatures using samples from the reciprocal transplant experiment. Each sample was incubated at its receptor site's mean summer temperature and those of the other recipient sites, resulting in three incubation temperatures (8°C, 16°C, 20°C) due to the similar mean summer temperatures of the Polish, Estonian, and Finnish sites.

### 2.7 Data Analyses

All data analyses were performed with R v4.3.3 (R Core Team 2023) and RStudio v0.9.1 + 401 (Posit Team 2025) using specific packages, as indicated afterwards. All visualisations were produced using the *ggplot2* package (Wickham 2016). All statistical analyses are detailed in Supporting Information: Methods.

#### 2.7.1 Collinearity Among Environmental Predictors

To minimise collinearity among environmental variables, we first compared monthly and long‐term climatic variables using correlation analyses. Because monthly and long‐term variables were highly correlated (*r* > 0.8), only long‐term variables were retained. We then used principal component analysis (PCA) to identify the main environmental gradients while selecting variables that were both weakly correlated and ecologically relevant for northern peatlands (see Supporting Information: Methods). Variable selection aimed to avoid redundant predictors (e.g., minimum and maximum temperature), while retaining key climatic (e.g., snow cover, precipitation), edaphic (e.g., soil pH), topographic (e.g., elevation), and ecosystem functioning (gross primary productivity) variables. Finally, clustering of variables (ClustOfVar; Chavent et al. 2012) was used to verify the consistency of the identified environmental gradients (Figure 1c; Figure S4).

#### 2.7.2 Bioclimatic Effects

Differences in bacterial community attributes and extracellular enzyme activities among the three bioclimatic regions were evaluated using linear models. Variables were transformed when necessary to satisfy model assumptions, which were assessed from residual diagnostics. Because sample sizes differed among regions, statistical significance was tested using Type III analysis of variance (ANOVA), followed by Tukey's honestly significant difference (HSD) tests for pairwise comparisons (Supporting Information: Methods).

#### 2.7.3 Gamma and Alpha Diversity

Rank‐abundance curves were computed for each SDP's bioclimatic area. Patterns of gamma diversity were explored by calculating both observed and estimated (asymptotic) ASV richness using iNEXT (Hsieh et al. 2016). For alpha diversity, we applied the Hill numbers framework, which enables effective comparison of diversity indices based on the “equivalent number” of features (Jost 2006). Accordingly, we report observed ASV richness (*q* = 0) and the effective number of ASVs derived from the exponential of Shannon entropy (*q* = 1), calculated using the function hill_taxa in the *R* package hillR (Li 2018) (v.0.5.2). To evaluate the effects of environmental parameters on ASV richness, we fitted generalised linear models. Assumptions of normality and homoscedasticity were visually assessed using model residuals.

#### 2.7.4 Specific, Unique, and Core ASVs

Following Ezzat et al. (2025), we classified ASVs based on their distribution and relative abundance. ASVs detected exclusively in a single bioclimatic area, but across multiple SDPs of that particular bioclimatic area, were classified as specific, whereas those found in only two SDPs within a particular bioclimatic area were classified as unique. ASVs present in more than nine SDPs (i.e., 5% of the SDPs) and detected at a relative abundance ≥ 0.1% were classified as widespread ASVs. To visualise the relative abundance of the most abundant phyla and families across core, specific, and unique ASVs, we further plotted the mean non‐null relative abundance across samples for each ASV against their prevalence in our dataset.

#### 2.7.5 Beta Diversity

To evaluate differences in community composition, we performed Principal Coordinate Analysis (PCoA) on Hellinger‐transformed ASV abundances, which minimises the influence of dominant species on site dissimilarity (Legendre and Gallagher 2001). Pairwise site dissimilarities were calculated using the Morisita‐Horn distance within the *phyloseq* package (McMurdie and Holmes 2013). We then applied Generalised Dissimilarity Modelling (GDM) (Ferrier et al. 2007) to quantify how environmental gradients and geographic distance drive ASV turnover among SDPs. GDM is a nonlinear extension of matrix regression that models the relationship between compositional dissimilarity and environmental or spatial predictors, allowing each predictor to contribute flexibly to the overall turnover pattern. The ASV matrix (reads per sample) was Hellinger‐transformed, and environmental predictors were standardised prior to analysis. Collinearity among predictors was assessed to select a non‐redundant set (see details above). Geographic distance between sites was also included as a predictor to account for spatial effects. Pairwise ASV dissimilarities were calculated using both Morisita‐Horn and Jaccard distances to capture differences in abundance‐weighted and presence‐absence patterns, respectively. Variable significance in the GDM was evaluated via matrix permutations, comparing models with each variable permuted versus unpermuted. At each step, the least important variables were sequentially removed until only significant predictors remained. The final GDM was then refitted using only significant environmental variables, and model significance was verified and its deviance (i.e., the variance explained by the model) calculated. Finally, variance partitioning was performed based on the contribution of each predictor to the total deviance explained by the final GDM model.

#### 2.7.6 Deterministic and Stochastic Community Assembly

We quantified the relative importance of deterministic and stochastic processes in bacterial community assembly using the two‐step framework based on the beta Nearest Taxon Index (βNTI) and the abundance‐weighted Raup–Crick metric (RC_bray) (Riddley et al. 2025). Ecological assembly processes were estimated for the complete dataset and separately for each bioclimatic region and bacterial richness region (see Results). Relationships between assembly processes and environmental variables were assessed using modified Mantel tests (Riddley et al. 2025).

Using data from the reciprocal transplant experiment, we further explored the importance of environmental filtering on bacterial communities. By comparing distances between transplanted communities and their original versus receptor sites, we evaluated whether transplanted bacterial communities retained signatures of their origin or converged toward local receptor communities, providing insight into the strength of environmental filtering during community assembly. Community similarity was quantified using Morisita–Horn (abundance‐based) and Jaccard (presence–absence) dissimilarities. We further compared community positions in Principal Coordinates Analysis (PCoA) ordination space by calculating Euclidean distances between transplanted communities and the centroids of their original and receptor communities using the PCoA axes explaining 80% of the total variation. Differences were assessed using ANOVAs.

#### 2.7.7 Drivers of Extracellular Enzyme Activities

Relationships between environmental variables and enzyme activities were first evaluated using LMs, with model assumptions assessed from residual diagnostics. Associations between individual ASVs and each enzyme were then assessed using MaAsLin2 (Mallick et al. 2021), applying log‐transformed relative abundances and Benjamini–Hochberg false discovery rate correction. To disentangle the direct and indirect effects of environmental conditions and bacterial community attributes on extracellular enzyme activity (EEA), we used structural equation modelling (SEM) (Grace et al. 2014). The model structure was defined a priori based on current knowledge of microbial and peatland functioning (Figure S5; Table S4). While the overall model structure was theory‐driven, the inclusion of environmental variables was, however, evaluated empirically among a set of pre‐identified environmental covariates to address potential collinearity and avoid overfitting (Grace et al. 2016). Our final model retained minimum temperature and pH as best environmental covariates (Table S5). The SEM model included two latent variables representing bacterial community composition (derived from PCoA axes) and overall extracellular enzyme activity (derived from the four measured enzymes). Models were fitted using the *lavaan* R package, and model adequacy was evaluated using χ^2^ tests together with RMSEA, SRMR and CFI. More details are given in Supporting Information: Methods.

#### 2.7.8 Identifying Thresholds in Ecosystem Functioning

To investigate whether changes in bacterial diversity were associated with non‐linear changes in ecosystem functioning, we applied smoothed structural equation modelling (Jassey et al. 2018). Moving‐window SEMs were fitted along the bacterial richness gradient using the same conceptual model as the global SEM, thereby quantifying changes in the relationships among bacterial richness, community composition, cell density and extracellular enzyme activity. The optimal window size was selected based on model convergence, goodness‐of‐fit and consistency of the resulting patterns (Supporting Information: Methods). Enzyme multifunctionality was represented by the latent EEA variable, and complementary multifunctionality metrics together with null‐model randomisations confirmed the robustness of this approach (Figures S6–S8). Generalised additive models (GAMs) were used to test for non‐linear relationships between bacterial richness and EEA, and broken‐line regression was applied to estimate tipping points in EEA along the richness gradient. The stability of the estimated threshold was evaluated across the range of suitable moving‐window sizes (Supporting Information: Methods).

## 3 Results and Discussion

### 3.1 Global Biodiversity Patterns

**FIGURE 2:**
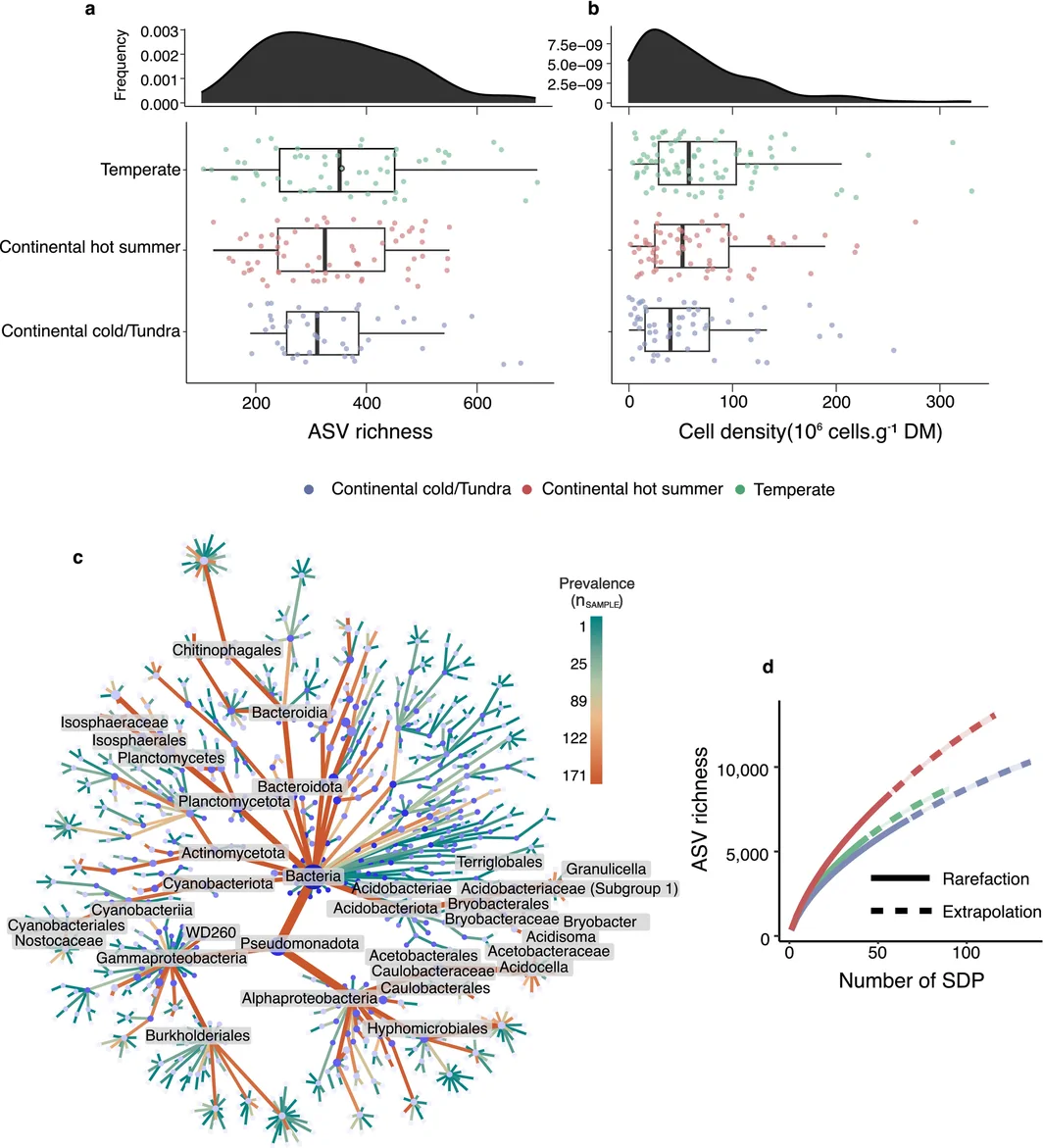
Global diversity and abundance patterns of the SDPs' microbiome. (a and b) Distribution of bacterial richness and cell density. Bacterial richness and cell density patterns across bioclimatic regions are illustrated in boxplots. Global distributions of estimated richness and cell density are shown in density curves above bioclimatic area‐level boxplots. Boxplots indicate the median (centre line), first and third quartiles (lower and upper box edges) and 1.5× the interquartile range (IQR) (box whiskers). The points shown represent all samples that passed quality control checks. (c) Heat tree illustrating the bacterial taxonomic structure, from domain to genus level, and highlighting the most prevalent lineages in SDPs. Only taxa with a minimum prevalence of 75% are identified. (d) Observed and estimated gamma diversity per bioclimatic area based on asymptotic estimates (*n* = 171 SDPs).

We expected low bacterial biodiversity and biomass in SDPs due to conditions of waterlogging, acidity, and poor nutrient availability (Van Breemen 1995). Consistent with this expectation, alpha diversity revealed mean richness values of 346 ASVs (interquartile range (IQR) 241–435; Figure 2a) and a Shannon H (based on Hill numbers) of 128 ASVs (IQR 89–183). These values fall within the range observed in other local and regional peatland studies (Cadillo‐Quiroz et al. 2019; Richy et al. 2024), but at the lower end of the spectrum for soil bacterial diversity more broadly (Delgado‐Baquerizo and Eldridge 2019; Labouyrie et al. 2023). This confirms that SDPs harbour less diverse bacterial communities than other organic‐ or mineral‐dominated soil systems, where environmental constraints are generally less severe (Andersen et al. 2013). Bacterial cell density was similarly low, with a median of 5.11 × 10^7^ cells per gram of dry moss (IQR 6.96 × 10^6^–1.98 × 10^8^ cells per gram of dry moss; Figure 2b), lower than values reported for other soil biomes (Fierer 2017). This suggests that peatland conditions constrain both bacterial diversity and bacterial growth.

Across all sites, we identified 472 bacterial genera across 125 bacterial orders and 47 phyla (Figure 2c). Bacteria from 10 orders represented 59.3% of all our reads, with Acetobacterales (34.1%), Terriglobales (12.3%), and Burkholderiales (3.09%) being the dominant orders (Figure 2c, Figure S9). Two genera, *Acidocella* and *Granulicella*, were particularly dominant across SDPs, accounting for 14.1% and 7.9% of all sequences, respectively (Figure S9). Moreover, a large proportion of bacterial diversity remained taxonomically unresolved, with 8618 ASVs (48.8% of all reads) unclassified at the genus level (Figure S9). To better understand how this diversity is distributed across the Holarctic region, we examined regional‐scale (gamma) biodiversity across the three bioclimatic regions using asymptotic estimates based on incidence data (see Methods). We sampled a median of 42.8% (IQR 40.9%–44.6%) of ASVs per bioclimatic area (Figure 2d), suggesting that our sampling provides a broadly representative view of bacterial biodiversity in SDPs. At the same time, these estimates imply that a substantial fraction of bacterial diversity remains to be discovered, particularly given our conservative filtering, denoising procedures, and sampling design (see Methods).

Rank abundance curves (Figure S10) show that a relatively high proportion of abundant ASVs (32.4% with a relative abundance above 0.1%) shape alpha diversity in SDPs. Comparisons with log‐normal rank abundance models (Figure S11) further suggest that SDPs are a highly selective environment, favouring a few dominant taxa and limiting the persistence of transient and rare taxa (Shoemaker et al. 2017). The hyperdominance of these few taxa likely underpins the consistent patterns of bacterial richness and cell density observed across bioclimatic regions (Figure 2a,b). However, despite their apparent stability, both bacterial richness and cell density show predictable variation along environmental gradients (Figures S12 and S13). Specifically, ASV richness declined with increasing minimum temperature (Spearman *ρ* = −0.21, *p* = 0.02) and pH (*ρ* = −0.29, *p* = 0.017), while it increased with higher soil moisture (*ρ* = 0.29, *p* < 0.01) and snow water equivalent (*ρ* = 0.2, *p* = 0.03; Figure S12).

While pH is a well‐known driver of bacterial richness in peatlands (Andersen et al. 2013) and in soils more broadly (Delgado‐Baquerizo and Eldridge 2019; Fierer 2017; Wang et al. 2022), our findings emphasise the additional importance of minimum temperature, soil moisture, and snow cover—three defining features of northern ecosystems. Snow accumulation insulates the soil, increasing its temperature and moisture and likely supporting bacterial richness by creating a more stable, resource‐rich environment that allows a broader range of microbial niches to persist (Buckeridge et al. 2010; Nemergut et al. 2013; Zinger et al. 2009). However, the observed decrease in ASV richness along the minimum temperature gradient contrasts with studies reporting increasing bacterial diversity with rising temperature (e.g., along elevation gradients (Nottingham et al. 2018)). This discrepancy may reflect the strong thermal sensitivity of SDP bacterial communities, which are adapted to low‐temperature conditions. Even modest warming can induce physiological stress and reduce bacterial diversity in communities outside their historical range, as observed in other cold‐adapted soil systems (Nottingham et al. 2022; Weedon et al. 2023). An alternative, non‐exclusive explanation is that the niche for SDP bacteria is particularly narrow. Given the dual constraints of low temperature and acidity, fewer taxa may possess the adaptations required to thrive under both conditions. Consequently, lower richness may stem not only from thermal stress, but also from the difficulty of adapting to such a demanding environment.

### 3.2 High Local and Regional Specificity

**FIGURE 3:**
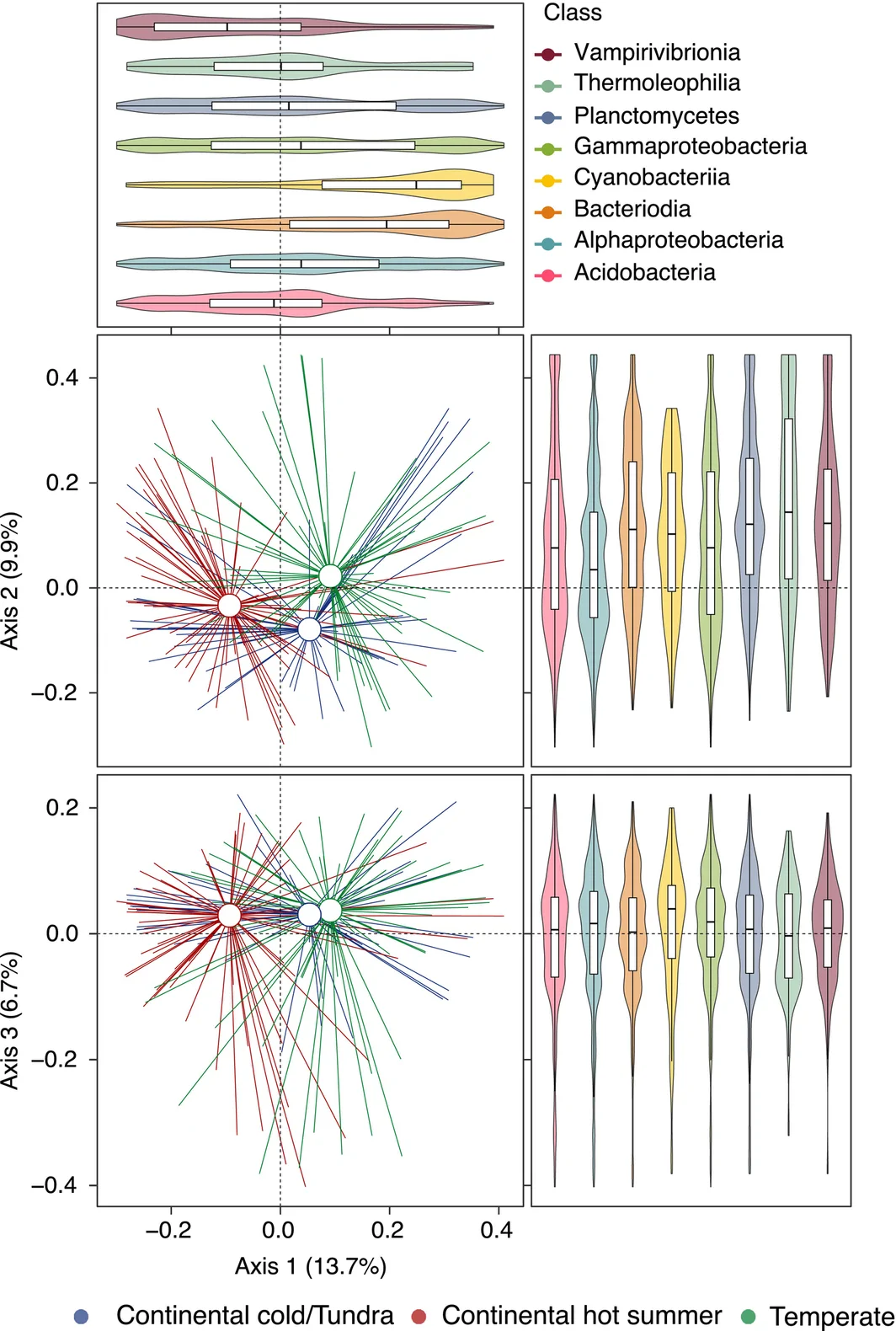
Bacterial community variations among and within bioclimatic regions. Biplots of PCoA1 and PCoA2 or PCoA3 scores from a PCoA describing variations in SDP bacterial communities. Colours separate bacterial communities by bioclimatic area. Boxplots depict differences in bacterial eight dominant classes along each PCoA axis. Boxplots indicate the median (centre line), first and third quartiles (lower and upper box edges) and 1.5× the interquartile range (IQR) (box whiskers). Violin areas show data distribution for each bacterial class (*n* = 171 SDPs).

Our data enabled us to highlight the spatial structure of the SDP bacterial microbiome. We found high community dissimilarities (Morisita‐Horn dissimilarity index), both within (0.79, IQR 0.78–0.81) and between (0.83, IQR 0.81–0.84) bioclimatic SDP ranges (Figure S14). This high beta diversity was driven by strong ASV turnover among SDPs, as shown by the high community dissimilarity indices derived from presence‐absence data (0.84, IQR 0.81–0.85; Figure S14b). Despite this consequent ASV turnover, we identified distinct biogeographical patterns in SDP bacterial composition, with a significant segregation among the three bioclimatic regions (Figure 3; *F* = 4.9, *p* < 0.05, PERMANOVA). The first PCoA axis mainly discriminated Continental hot summer SDPs (on the left) from the other bioclimatic regions (on the right). Continental hot summer SDPs were associated with Vampirivibrionia, while Cyanobacteriia and Bacteroidia characterised Temperate and Continental cold/Tundra SDPs (Figure 3). The second PCoA axis mainly discriminated Continental cold/Tundra from Temperate bioclimatic regions, where Temperate SDPs were positively associated with Thermoleophilia, Planctomycetes, and Bacteroidia (Figure 3). The third PCoA axis did not clearly discriminate among the bioclimatic regions (Figure 3). These pronounced dissimilarities among SDP bioclimatic regions emphasise the relevance of regionality for the compositional turnover of the SDP microbiome.

To further characterise the beta diversity patterns across bioclimatic ranges, we explored the specificity of ASVs within SDP bacterial communities. In other words, we investigated how many ASVs occurred in less than 5% of SDPs (hereafter referred to as ‘specific’ ASVs, see Methods). Overall, 92.1% of all ASVs were specific, with 61.1% unique to a single SDP and 31% present in fewer than 10 SDPs. These specific and unique ASVs occurred at relatively high abundances (28%, Figure S14a), underscoring their importance for overall SDP bacterial biodiversity. Only 8% of all ASVs were consistently detected across multiple SDPs, and just 13 ASVs (0.3% of all ASVs) were ubiquitous (prevalence > 75%; Figure S15a). This pattern mirrors global soil bacterial communities, where only about 0.4% of bacterial species are widespread across bioclimatic ranges (Bickel and Or 2021).

ASV‐specificity was not uniformly distributed across SDP bioclimatic regions (Figure 15b), with the Temperate region exhibiting the highest specificity (57.1% of specific and unique ASVs). This contrasts with the low endemicity typically observed in soil fungal communities (Tedersoo et al. 2022) but aligns with island biogeography theory. Temperate SDPs, often fragmented (< 5 ha) in the landscape (Xu et al. 2018), act as biogeographical ‘islands’ within the extensive boreal and sub‐arctic peatlands, suggesting that spatial isolation, ecological drift, and long‐term climate stability promote speciation and endemism in these peatlands. We caution that our results do not unequivocally demonstrate ASV endemism in SDPs. However, they add to the ongoing debates on microbial endemism (Gilbert et al. 2025) and advocate for the need to integrate microbial conservation into peatland management plans.

### 3.3 Ecological Processes Governing Bacterial Community Assembly

To elucidate the processes underlying the biogeographic patterns observed across bioclimatic regions, we employed generalised dissimilarity modelling (GDM), which quantifies the relationships among beta diversity, geographic and environmental distances. GDM explained 27% (*p* < 0.001) of the variation in bacterial community composition. Climate (i.e., precipitation, snow water equivalent, minimum air temperature; 61% of the explained variation) was more important in driving bacterial turnover at a large spatial scale than geography (18%) and local conditions (i.e., soil moisture, elevation, and percentage of non‐tree cover; 20%; Figure S16a). This aligns with a growing body of evidence showing that climatic factors often outweigh local environmental factors in shaping microbial community composition (Bahram et al. 2018; Delgado Baquerizo et al. 2018). More particularly, precipitation, snow water equivalent, and minimum temperature significantly explained bacterial dissimilarity, while soil moisture, elevation, and non‐tree cover only had marginal effects (Figure S16b). The effect of geographic distance increased continuously along the distance gradient (Figure S16b), which aligns with the classic distance‐decay relationship found in soil microbiomes (Bahram et al. 2018). Most of the effect of precipitation and snow water equivalent occurred within the first 50% of their variation gradient (Figure S16b), while the effect of minimum temperature and elevation was strongest above 50% of their variation (Figure S16b). These results indicate that precipitation, snow and temperature are important environmental filters of SDP bacterial communities. Additionally, GDM showed that a substantial portion of the bacterial community variation (73%) remained unexplained, suggesting that bacterial communities may be strongly influenced by unmeasured local environmental conditions, such as nutrient availability and plant community composition (Lamit et al. 2021; Richy et al. 2024), as well as by stochastic processes, including homogenising dispersal, dispersal limitation, and ecological drift (Riddley et al. 2025).

**FIGURE 4:**
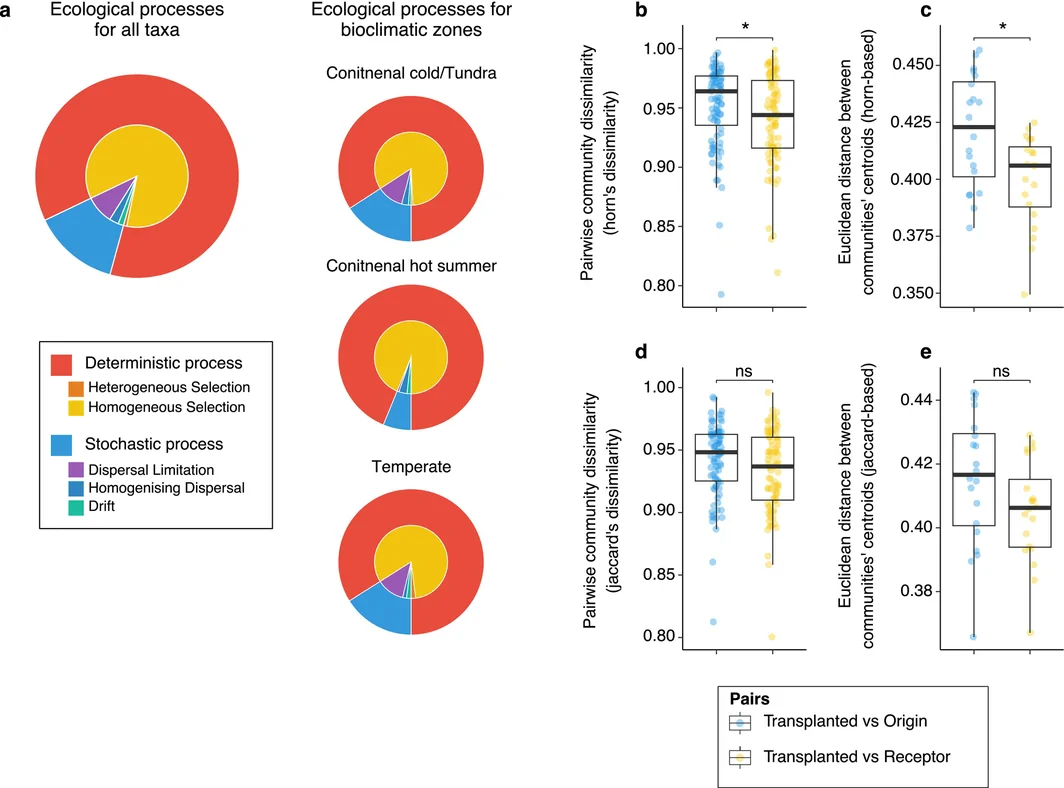
Deterministic and stochastic processes play different roles in the SDP bacterial community. (a) Quantified importance of ecological processes to the community assembly for all taxa and each bioclimatic region. (b) Reciprocal transplant experiment assessing the relative influence of contemporary environmental selection versus historical processes on bacterial community composition (see Methods). Panels show pairwise‐dissimilarities between transplanted and receptor‐site or donor‐site communities evaluated using (a) Horn‐based taxonomic dissimilarities (relative abundance) and (c) Jaccard‐based dissimilarities (presence–absence) (*n* = 70). Euclidean distances between transplanted and receptor‐site or donor‐site community centroids in PCoA space based on Horn‐based taxonomic dissimilarities (b) and Jaccard‐based dissimilarities (d) (*n* = 25). Boxplots indicate the median (centre line), first and third quartiles (lower and upper box edges) and 1.5× the interquartile range (IQR) (box whiskers). Letters indicate significant differences between treatments (ANOVAs).

We found that deterministic processes, and especially homogeneous selection, accounted for almost 80% of bacterial community assembly across SDPs (Figure 4a), which differed from previous observations in wetlands and soils in general (Riddley et al. 2025). Mantel tests for asymmetric matrices revealed significant correlations between environmental factors (excluding pH) and deterministic processes, but not between stochastic processes and environmental factors (Figure S17). These results suggest that environmental factors trigger deterministic processes that govern bacterial community assembly in SDPs and promote phylogenetically similar community structures across locations (Riddley et al. 2025; Zhou and Ning 2017). Furthermore, the predominance of deterministic processes indicates strong local environmental selection in SDPs (Riddley et al. 2025), corroborating potentially high levels of regional endemism across bioclimatic regions. This interpretation is directly supported by our reciprocal transplant experiment. Specifically, we tested whether bacterial communities exposed to novel climatic and local conditions would converge towards the composition of receptor‐site communities (indicating contemporary environmental selection) or instead retain characteristics of their original sites (reflecting historical processes such as dispersal limitation, drift and past environmental selection). Analyses based on relative abundance and presence‐absence dissimilarity indices showed that transplanted communities exhibited greater similarity to receptor‐site communities than to their original sites (Figure 4b,c). However, this pattern was only observed using the relative abundance‐based index, suggesting that shifts in ASV relative abundances, rather than species turnover, are more sensitive to contemporary environmental filtering (Figure 4b–e). Together, these findings emphasise the vulnerability of SDP bacterial communities to environmental disturbances and suggest that ongoing environmental change could lead to significant biodiversity loss and ecological degradation in northern peatlands.

### 3.4 Peatland Functions Are Sensitive to Bacterial Biodiversity Loss

Extracellular enzyme activities (EEA) varied significantly among bioclimatic regions, peaking in the Continental hot summer and Temperate regions (*p* < 0.05, ANOVAs; Figure S18). Enzyme activities were primarily related to climatic variables (Figure S19). This contrasts with previous soil studies, in which pH was generally regarded as the most significant factor influencing enzyme activity (Sinsabaugh et al. 2008). This discrepancy is likely explained by the relatively narrow pH range across the studied SDPs (Figure S20). Importantly, this observation aligns with our results on bacterial richness and community composition, which also demonstrated a limited pH influence (Figures S12, S13, and S16).

To determine whether changes in EEA across the environmental gradient are mediated by bacterial biodiversity, we employed structural equation modelling (SEM; see Methods). Overall, our SEM showed that environmentally driven shifts in bacterial richness strongly structured bacterial community composition (39% of the variance explained) and cell density (8% of the variance explained), which together accounted for 68% of the variation in EEA (Figure S21). This finding indicates that bacterial richness may regulate C and nutrient cycling in peatlands primarily by restructuring bacterial communities rather than simply by altering overall cell density. To identify the taxa underlying this relationship, we examined associations between bacterial ASVs and enzyme activities. We identified 123 ASVs (0.8% of the total bacterial richness, but 6.5% of total reads) whose relative abundance correlated with enzyme activities (Figure S22a,b). These ASVs belonged to Alphaproteobacteria, Acidobacteria, Planctomycetes, Gammaproteobacteria, and Bacteroidia (Figure S22c), which corroborates prior soil studies (Trivedi et al. 2016) and genomic evidence (Trivedi et al. 2013).

### 3.5 Critical Thresholds in Bacterial Richness Drive EEA

**FIGURE 5:**
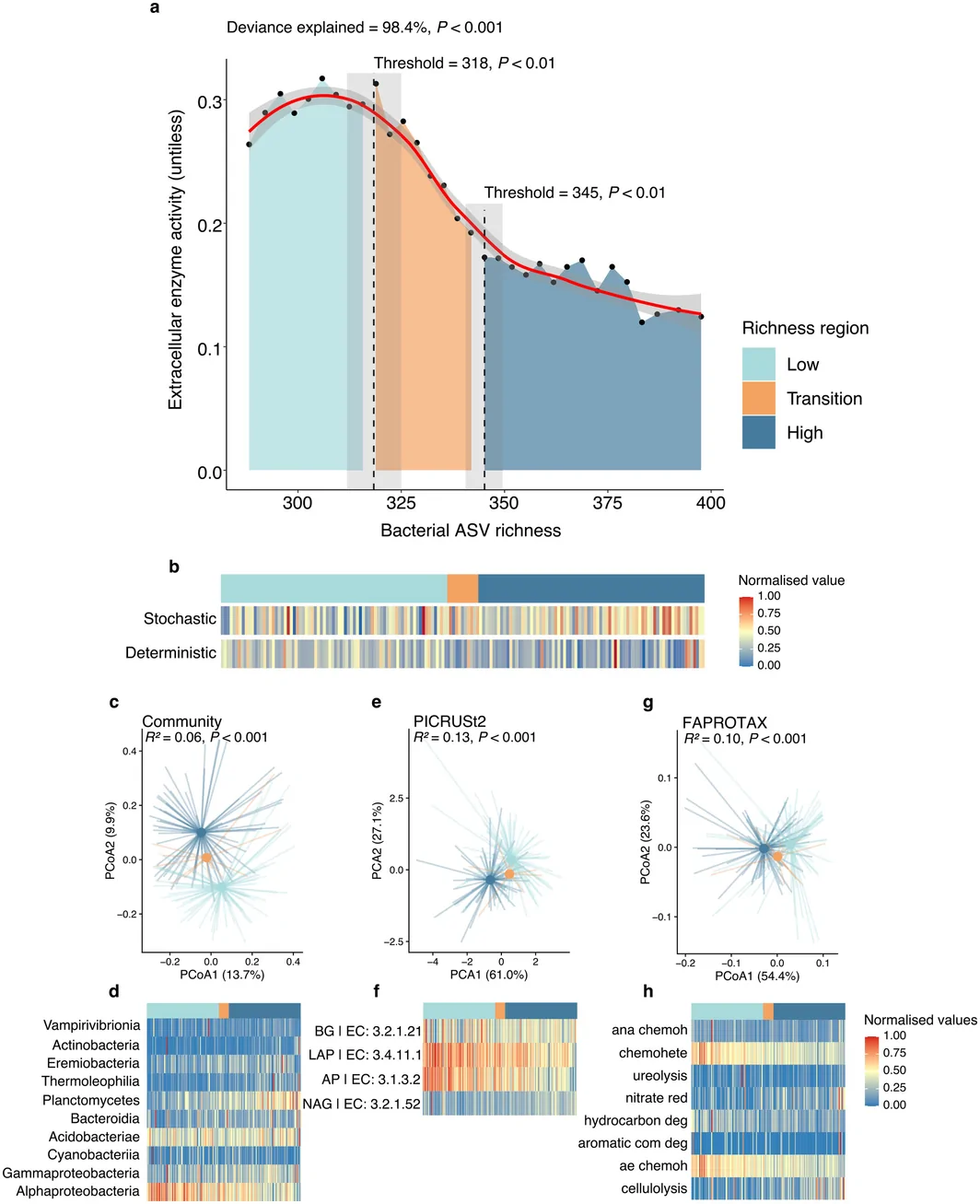
Breaking points in bacterial biodiversity loss amplify extracellular enzyme activity (EEA). (a) Moving window structural equation model showing the response of EEA (75th percentile, latent variable in SEM models, see Methods) to increasing bacterial richness. Open symbols indicate SEMs with unsatisfactory model fit, whereas filled symbols indicate satisfactory model fit. The full a priori SEM is shown in Figure S5 and the global SEM results in Figure S21. Dashed lines indicate the estimated breakpoints identified by broken‐line regression. Deviance explained was calculated using a generalized additive model (GAM). Gray bars represent the confidence interval of breakpoint locations estimated across window sizes of 85–95 samples (see Methods). (b) Heatmaps showing the relative contribution of deterministic and stochastic community assembly processes along the bacterial richness gradient. (c) Principal coordinates analysis (PCoA) of bacterial community composition across bacterial richness regions. (d) Heatmaps showing changes in the relative abundance of dominant bacterial classes across the bacterial richness gradient. (e) Principal component analysis (PCA) of predicted EC numbers associated with the four measured extracellular enzymes (PICRUSt2) across bacterial richness regions. (f) Heatmaps showing changes in the relative abundance of EC numbers associated with the four measured extracellular enzymes across the bacterial richness gradient. (g) PCoA of FAPROTAX‐based functional annotations across bacterial richness regions. (h) Heatmaps showing changes in the relative abundance of dominant bacterial functional annotations across the bacterial richness gradient. Abbreviations are: ana chemoh = anaerobic chemoheterotrophy, chemohete = chemoheterotrophy, nitrate red = nitrate reductase, hydrocarbon deg. = hydrocarbon degradation, aromatic com deg. = aromatic compounds degradation, ae chemoh = aerobic chemoheterotrophy.

The smoothed SEM approach revealed that EEA increased non‐linearly with bacterial biodiversity loss (Figure 5a, Table S6), suggesting that EEA is constrained at high levels of bacterial richness but increases sharply beyond a critical threshold. A broken line model revealed two breaking points that delineated three distinct richness regions: a high bacterial richness region (345–400 ASVs), where EEA is constrained and remained stable; a transitional region (between 318 and 345 ASVs), where EEA begins to increase; and a low ASV richness region (< 318 ASVs), where EEA reaches its highest levels (Figure 5a). A randomisation exercise (see Methods) further confirmed that this non‐linear trend was unlikely to result from sampling effects or spatial structure (Figure S8).

The three bacterial richness regions significantly differed in community assembly processes (Figure 5b). High‐diversity communities exhibited a greater contribution of stochastic processes (*F*_*1,2*_ = 5.4, *p* < 0.01, ANOVA), whereas deterministic processes increasingly dominated as richness declined (*F*_*1,2*_ = 3.5, *p* < 0.05, ANOVA; Figure 5b). This shift in assembly processes coincided with significant changes in community composition (PERMANOVA, *p* < 0.01; Figure 5c), including increases in the relative abundance of Alphaproteobacteria and declines in Planctomycetes, Gammaproteobacteria and Eremiobacteria (Figure 5d). This taxonomic turnover was accompanied by changes in predicted functional potential: the abundance of functional genes associated with the four measured enzymes differed significantly among bacterial richness regions (PERMANOVA, *p* < 0.01, Figure 5c). Specifically, EC numbers associated with LAP and AP enriched under low bacterial richness (Figure 5d). This pattern was further supported by a strong correlation between the abundance of the EEA‐associated ASVs and EC numbers associated with enzymes (*r* = 0.73, *p* < 0.001), further linking changes in community composition to the observed increase in enzyme activities. Consistent with these results, FAPROTAX‐based functional annotations also differed significantly among bacterial richness regions (PERMANOVA, *p* < 0.01; Figure 5c), revealing a shift towards heterotrophic functions such as aerobic chemoheterotrophy, chemoheterotrophy and hydrocarbon degradation, as bacterial richness declined (Figure 5d). Together, these results suggest that bacterial richness exhibits a critical threshold at approximately 345 bacterial ASVs (CI: 338–348), marking a transition in community assembly and composition towards communities with distinct functional potential. Although the exact richness value is expected to vary with methodological choices (e.g., ASV filtering) (Nearing et al. 2022), the existence of a threshold separating functionally distinct community states appears robust (Figure 5, Figures S6b and S8).

### 3.6 General Implications

Overall, our trans‐Holarctic survey clearly shows that peatland bacterial communities are not only taxonomically diverse but also display high regional and local specificity. This diversity is mainly shaped by strong and persistent environmental filters within bioclimatic regions, reflecting the long‐standing selective regime imposed by SDPs (Treat et al. 2019). This supports previous research where deterministic processes are more influential in bacterial assembly in highly selective systems (Riddley et al. 2025). Our functional assessments further highlight bacterial richness as an important driver regulating peatland functions on a trans‐Holarctic scale. We show that the observed increase in EEA across environmental gradients is not simply a consequence of bacterial richness loss per se, but reflects a broader reorganisation of bacterial communities along the gradient. Below the richness threshold, bacterial communities were increasingly assembled through deterministic processes, indicating stronger environmental filtering. Rather than representing impoverished versions of more diverse communities, these assemblages constitute a distinct ecological state characterised by taxa with greater predicted capacities for extracellular nutrient acquisition and heterotrophic metabolism. Under the resource constraints characteristic of peatlands, environmental selection may therefore favour life‐history strategies that prioritise investment in extracellular enzymes to enhance access to carbon and nutrient resources (Malik et al. 2020; Sinsabaugh and Shah 2012). Conversely, high‐bacterial diversity SDPs showed a greater contribution of stochastic assembly, leading to more variable taxonomic composition but stable community‐level EEAs. This aligns with the idea that stochastic processes create multiple taxonomic configurations capable of maintaining similar ecosystem functioning through functional redundancy (Louca et al. 2018; Zhou and Ning 2017). Although environmental filtering provides the most parsimonious explanation for our observations, other non‐exclusive mechanisms may also contribute. For example, reduced interspecific competition in low‐diversity communities could enable the remaining taxa to allocate more resources to extracellular enzyme production (Folse III and Allison 2012), a process known to be energetically costly (Ramin and Allison 2019). Moreover, we cannot exclude that environmental differences in substrate quality or resource availability also contribute to these patterns by simultaneously influencing bacterial diversity, community composition and enzyme production (Allison and Vitousek 2005; Fierer 2017; Sinsabaugh and Shah 2012).

Against the backdrop of ongoing climate warming, which has been shown to reduce bacterial diversity over the long term (Sun et al. 2025), these results take on added significance. Warming‐induced biodiversity loss could push peatland microbial communities across the bacterial richness threshold identified here, shifting them towards the low‐richness state characterised by elevated EEA. Because elevated enzyme activities are associated with increased respiration rates (Jassey et al. 2018), this ecological transition could ultimately accelerate C loss from peatlands. Incorporating bacterial richness and nonlinear functional thresholds into Earth system models could significantly improve predictions of peatland responses to environmental change. Although this entails scaling up from local to global levels (Lennon et al. 2024), such efforts could transform our ability to forecast peatland responses to environmental change and refine global carbon budgets.

While our findings advance the understanding of microbial controls on soil C cycling, they are not exempt from limitations. Our large‐scale sampling only captures a snapshot of bacterial diversity in SDPs and may therefore include transient communities while overlooking the contribution of slow‐growing and dormant taxa to community structure and enzyme activities. Nevertheless, the relative stability of bacterial communities across seasons (Richy et al. 2024) suggests that our sampling still provides a representative view of the core bacterial diversity in SDPs. While standardising enzymatic assays improves comparability across bioclimatic regions, it may also introduce bias by homogenising enzyme activities and obscuring the true functional diversity, particularly in cold‐adapted communities (Marasco et al. 2023). To address this, we supplemented our trans‐Holarctic approach with the reciprocal transplant experiment and expanded our enzymatic assays to include multiple incubation temperatures. This enabled us to capture both the potential and realised enzyme activities under diverse thermal regimes (see Methods). Our findings confirm that, while higher incubation temperatures elevate enzyme activities, they do not alter the observed biogeographical patterns (Figure S23). This consistency across temperature regimes highlights a critical implication: the simplification of SDP bacterial communities driven by environmental change may destabilise peatland biogeochemical cycles. This makes peatlands more vulnerable to external conditions and more dependent on a limited set of taxa for their functioning, thereby threatening the long‐term stability of the C pool. Given the isolation of many peatlands, natural recolonisation is unlikely, leaving microbiomes permanently altered and less resilient. Preserving microbial diversity in these ecosystems is therefore essential for maintaining peatland resilience and safeguarding their role in global climate regulation.

## Author Contributions

**Vincent E. J. Jassey:** conceptualization, investigation, funding acquisition, writing – original draft, methodology, validation, visualization, writing – review and editing, formal analysis, project administration, data curation, supervision, resources. **Tristan Lafont Rapnouil:** writing – original draft, visualization, writing – review and editing, formal analysis, data curation. **Marie Le Geay:** writing – review and editing, visualization, validation, methodology. **Romain Walcker:** investigation, writing – review and editing, methodology, resources. **Laure Gandois:** investigation, writing – review and editing, conceptualization. **Maialen Barret:** investigation, conceptualization, writing – review and editing. **Nicolas Fanin:** methodology, validation, visualization, writing – review and editing. **Bjorn J. M. Robroek:** validation, visualization, writing – review and editing. **Béatrice Lauga:** methodology, visualization, validation, writing – review and editing. **Luke O. Andrews:** writing – review and editing, resources. **Alix Badré Greuza:** resources, writing – review and editing. **Mark R. Bakker:** writing – review and editing, resources. **Guillaume Bertrand:** writing – review and editing, resources. **Tobias Birkwald:** writing – review and editing, resources. **Paulo A. V. Borges:** writing – review and editing, resources. **Brian Branfireun:** writing – review and editing, resources. **Francis Q. Brearley:** writing – review and editing, resources. **Cécile Brousseau:** resources, writing – review and editing. **Zhao‐Jun Bu:** resources, writing – review and editing. **Jean‐François Carrias:** resources, writing – review and editing. **Sarah Coffinet:** resources, writing – review and editing. **Bruno Corbarra:** resources, writing – review and editing. **Eduardo M. F. Dias:** resources, writing – review and editing. **Ellen Dorrepaal:** conceptualization, writing – review and editing. **André‐Jean Francez:** resources, writing – review and editing. **Yuwen Fu:** resources, writing – review and editing. **Marvin Gabriel:** resources, writing – review and editing. **Rosalina Gabriel:** resources, writing – review and editing. **Stéphanie Gerin:** resources, writing – review and editing. **Daniel Gilbert:** resources, writing – review and editing. **Sébastien Gogo:** resources, writing – review and editing. **Gustaf Granath:** resources, writing – review and editing. **Emma Gray:** resources, writing – review and editing. **Liam Heffernan:** resources, writing – review and editing. **Thierry J. Heger:** resources, writing – review and editing. **Sari Juutinen:** resources. **Paul Kardol:** resources, writing – review and editing. **Edgar Karofeld:** resources, writing – review and editing. **Agata Klimkowska:** resources, writing – review and editing. **Martin Küttim:** resources, writing – review and editing. **Liisa Küttim:** resources, writing – review and editing. **Olivia Kuuri‐Riutta:** resources, writing – review and editing. **Terri Lacourse:** resources, writing – review and editing. **Mariusz Lamentowicz:** resources, writing – review and editing. **Gaël Le Roux:** resources, writing – review and editing. **Zoë Lindo:** resources, writing – review and editing. **Roy Mackenzie:** resources, writing – review and editing. **Katarzyna Marcisz:** resources, writing – review and editing. **Yuri A. Mazei:** resources, writing – review and editing. **Natalia G. Mazei:** resources, writing – review and editing. **Cândida M. F. Mendes:** resources, writing – review and editing. **Edward A. D. Mitchell:** resources, writing – review and editing. **Juanita Mora‐Gomez:** resources, writing – review and editing. **Raphael Müller:** resources, writing – review and editing. **Kathy Pouliot:** resources, writing – review and editing. **Anna‐Helena Purre:** resources, writing – review and editing. **Achim Quaiser:** resources, writing – review and editing. **Brenda Riquelme‐del Río:** resources, writing – review and editing. **Allison R. Rober:** resources, writing – review and editing. **Line Rochefort:** resources, writing – review and editing. **Ana C. Rodríguez:** resources, writing – review and editing. **A. Britta K. Sannel:** resources, writing – review and editing. **Fabian Seemann:** resources, writing – review and editing. **Laurent Servière:** resources, writing – review and editing. **Maria Strack:** writing – review and editing, resources. **Graeme T. Swindles:** resources, writing – review and editing. **Julie Talbot:** resources, writing – review and editing. **Andrey N. Tsyganov:** resources, writing – review and editing. **Eeva‐Stiina Tuittila:** resources, writing – review and editing. **Alexander T. Tveit:** resources, writing – review and editing. **Minna M. Väliranta:** resources, writing – review and editing. **Kevin H. Wyatt:** writing – review and editing, resources. **Henni Ylänne:** writing – review and editing, resources. **Zicheng Yu:** writing – review and editing, resources. **Evgeny A. Zarov:** writing – review and editing, resources. **Samuel Hamard:** writing – review and editing, methodology, investigation, conceptualization, formal analysis.

## Funding

This work was supported by the French National program EC2CO (Ecosphère Continentale et Côtière), as well as by MIXOPEAT (Grant ANR‐17‐CE01‐0007), BALANCE (Grant ANR‐23‐ERCC‐0001‐01), and MICE (Grant ANR‐24‐CE02‐6475) projects, all funded by the French National Research Agency to V.E.J.J.

## Conflicts of Interest

The authors declare no conflicts of interest.

## Supporting information


**Data S1:** gcb71099‐sup‐0001‐supinfo01.pdf.

**Table S1:** Coordinates and Köppen climate classification of the different SDPs included in this study.

**Table S2:** Details of the different environmental predictors used in this study.

**Table S3:** Enzymes included in this study.

**Table S4:** Components of hypotheses represented by the structural equation model.

**Table S5:** Summary of the environmental variable selection within SEM process. Best model is highlighted in bold.

**Table S6:** Model fit of each SEM window.

**Figure S1:** A comparison of the bacterial community structure within‐site (different microhabitats) and between‐sites (same microhabitat).
**Figure S2:** Relationship between the pH measured in the field and the one retrieved from OpenLand.
**Figure S3:** Rarefaction curves of bacterial amplicon sequence variants (ASVs) for the 171 SDPs.
**Figure S4:** Environmental variable selection.
**Figure S5:** A priori conceptual structural equation model (SEM).
**Figure S6:** Selection of the moving‐window size for the smoothed SEM.
**Figure S7:** Comparison of two approaches to quantify enzyme multifunctionality, and the latent SEM variable used here.
**Figure S8:** Effect of randomization on the link between EEA (75th percentile of the latent variable) and bacterial ASV richness in smoothed SEMs.
**Figure S9:** Bacterial community composition.
**Figure S10:** Rank abundance model fits.
**Figure S11:** Rank‐abundance model fits.
**Figure S12:** Linkages between bacterial ASV richness and geographic and environmental parameters.
**Figure S13:** Linkages between bacterial cell density and geographic and environmental parameters.
**Figure S14:** Taxonomic turnover along geographic distance.
**Figure S15:** Components and taxonomy of the SDP microbiome.
**Figure S16:** Environmental drivers of bacterial turnover in SDPs.
**Figure S17:** Linkages between deterministic and stochastic processes and main environmental variables.
**Figure S18:** Extracellular enzyme activities in SDPs.
**Figure S19:** Main drivers of enzyme activities in SDPs.
**Figure S20:** Range of key environmental variables across SDPs.
**Figure S21:** Structural equation modelling (SEM) depicting the linkages between environmental variables, bacterial features (community composition, ASV richness and cell density), and enzyme activities.
**Figure S22:** Linkages between ASVs and enzyme activities.
**Figure S23:** Incubation temperature effect on enzyme activities.

## Data Availability

Source data are archived on Zenodo (https://doi.org/10.5281/zenodo.18837024) and GitHub (https://github.com/vjassey/SDP_16S_MS). The 16S rRNA sequencing reads will be deposited at NCBI Sequence Read Archive upon publication. Raw sequence data are available from the corresponding author upon reasonable request. The data will be made publicly available following completion of ongoing analyses.
