# Supplementary material for: Tipping Points in Bacterial Richness Amplify Extracellular Enzyme Activities in Northern Peatlands
Source: Glob Chang Biol. 2026 Sep 17;32(9):e71099. doi: 10.1111/gcb.71099 (PMC13586543; doi:10.1111/gcb.71099)
Supplement: Supplementary file 1 [file GCB-32-e71099-s001.pdf]

# Supplementary methods

## Tipping points in bacterial richness amplify extracellular enzyme activities in northern peatlands

Authors

Vincent E.J. Jassey , Tristan Lafont Rapnouil, *et al*[#].

[#]A full list of authors and their affiliations appears on the paper.

Contact: vincent.jassey@utoulouse.fr

## Bacterial diversity and community composition

**DNA extraction, library preparation and sequencing.** DNA was extracted from *Sphagnum* using the DNeasy PowerSoil Pro Kit (Qiagen) according to the manufacturer's instructions. We amplified the V3-V4 hypervariable region of bacterial 16S ribosomal RNA gene using the primer pair PCR1_515 forward (5'-GTGYCAGCMGCCGCGGTA-3') and PCR1_909 reverse (5'- CCCCGYCAATTCMTTTRAGT-3') (Jassey, Hamard, et al., 2022). PCRs were performed in a total volume of 50 µL, containing 25 $\mu$L of Amplitaq Gold polymerase (Applied Biosystem, ThermoFisher), 1 $\mu$L of each primer (20 $\mu$M at final concentration), 21 $\mu$L of ultrapure water, and 2 $\mu$L of template DNA. Thermal conditions applied following initial denaturation at 95 °C for 10 min were 94 °C for 60 s, 55 °C for 40 s and 72 °C for 30 s for 35 cycles, with a final extension at 72 °C for 10 min. Amplification quality was subsequently verified on a 1.5% agarose gel. Amplicon libraries were prepared according to the MiSeq manufacturer's protocol. In short, PCR was conducted for the addition of unique indices for each sample together with Illumina tags, allowing for extensive multiplexing of samples on a single sequencing lane of the MiSeq (Illumina) platform. Samples were sequenced using a 250-base-paired-end protocol at the Genotoul platform, Toulouse, France. Blanks were performed throughout the different analyses and subsequently sequenced to check for eventual contamination. All molecular work was carried out in a dedicated laboratory with UV-decontaminated material.

**Metabarcoding.** Paired-end sequencing generated a total of 38,473,476 × 2 reads, with a mean read number of 182,047 ± 51,388 per SDP. Amplicon sequences were initially processed using Quantitative Insights into Microbial Ecology 2 (QIIME2, 2020.8). Primers were trimmed from the raw sequences with the cutadapt plugin and demultiplexed with demux. Read quality was assessed using the interactive quality plots. Sequences were then processed using a Snakemake-encapsulated DADA2 workflow with the following trimming parameters: trimLeft=20 and trimRight=70. These steps resulted in a total of 6,421,253 reads and 19,182 ASVs. Taxonomy was assigned against the SILVA reference database (v.138.2) (Quast et al., 2013). ASV and taxonomy tables, along with the metadata, were transferred to the *R* (v.4.1) environment for subsequent statistical analyses. Eukaryote, Archaea, mitochondria, and chloroplast sequences were discarded. We then applied a prevalence threshold to our dataset such that only ASVs present in at least two SDPs were retained. Finally, sequencing depth was rarefied to 7,000 reads per sample. This threshold was selected to retain all samples (n = 171) in the dataset, as the rarefaction curves for ASV richness plateaued well before this depth (Fig. S3), indicating sufficient sampling effort. Additionally, taxonomic assignation with a bootstrap value inferior to 75% as returned by DADA2 was considered uncertain, and the corresponding taxonomic level was changed to "Unknown [taxonomic level]" (*e.g.* Unknown Genus), and ASVs not assigned at the Kingdom level were removed from the dataset.

The phylogenetic tree of ASVs was constructed based on the full sequence alignment of the 16S rRNA gene using maximum likelihood phylogenetic analysis with the GTR substitution model and 1,000

bootstraps. Trees were optimised using stochastic rearrangements and NNI moves to refine topology and branch lengths for high-confidence microbial community comparisons.

**Prediction of bacterial functional potential.** The functional potential of bacterial communities was inferred from 16S rRNA gene sequences using PICRUSt2 (Douglas et al., 2020). Amplicon sequence variants (ASVs) were placed into a reference phylogeny, and gene family abundances were predicted using hidden-state prediction. Predicted gene families were subsequently collapsed into Enzyme Commission (EC) numbers. To facilitate comparison with measured extracellular enzyme activities (EEA), we retained only EC numbers corresponding to the four enzymes quantified in this study (Table S3). To provide an independent assessment of bacterial functional potential, ASVs were also annotated using FAPROTAX (Stilianos Louca et al., 2016), which assigns ecological functions to prokaryotic taxa based on curated taxonomic information from cultured representatives. Functional annotations were generated using microeco *R* package (Liu et al., 2021), and the relative abundances of annotated functions were calculated for each sample. From these annotations, we kept only the main functions related to enzyme activities, and used them to evaluate changes in the distribution of broad bacterial functional groups across our gradients. Because both PICRUSt2 and FAPROTAX infer functional potential from taxonomic composition rather than directly measuring gene content or activity, they were solely used as complementary approaches to support patterns observed in measured extracellular enzyme activities.

## Extracellular enzyme activities

The relative activity of four hydrolytic enzymes commonly associated with microbial metabolic rates and biogeochemical properties (Galen, 2025; Sinsabaugh et al., 2008) was quantified using fluorogenic substrates labelled with 7-amino-4-methylcoumarin (MUC) or methylumbelliferone (MUB; see Table S4): ß-1,4-glucosidase (BG; breaks down cellulose into glucose for C acquisition), ß-1,4-N-acetylglucosaminidase (NAG; breaks down chitin and peptidoglycan for N and C acquisition), leucine aminopeptidase (LAP; breaks down proteins and polypeptides for N acquisition), and acid phosphatase (AP; mineralises organic phosphorus). All substrates were from Sigma-Aldrich, Switzerland (Weedon et al., 2014). Enzyme proteins were extracted following Criquet et al. (1999). Briefly, 3 g (fresh weight) of powdered *Sphagnum* was agitated for one hour in Pyrex bottles with 50 mL of a 0.1 M $CaCl_2$ solution with 0.05% Tween 80 and 20 g of polyvinylpolypyrrolidone. Following agitation, the suspension was pre-filtered through a colander to remove floating debris and centrifuged at 10,000 *g* for 10 min at 4°C. The resulting supernatant was filtered through 1.2 $\mu$m Whatman GF/D filters. The filtrate was then concentrated for 24h in a cellulose-dialysis tube (10 kDa molecular mass cut-off; Medicell International Ltd., London, UK) covered with polyethene glycol (PEG, Sigma-Aldrich, St Louis, MO, USA). Finally, the concentrated enzyme extract was re-diluted in a phosphate buffer (pH 5.6,corresponding to the mean pH of all samples) to achieve a final volume equivalent to 1/10 of the initial volume. Enzymatic rates were assessed in 96-well

microplates under saturating substrate conditions, and so reflected potential activities. For each sample, four pseudo-replicate wells received 38 $\mu$L of enzyme extract and 250 $\mu$L of enzyme substrate. An additional four wells, serving as controls, contained 38 $\mu$L of boiled enzyme extract (3 h at 90 °C) and 250 $\mu$L of enzyme substrate.

To ensure comparability across all sites in the trans-Holarctic survey, enzyme activities were initially incubated at a standardised temperature of 25°C for 3 hours. This approach minimises variability introduced by differing assay conditions and provides a consistent baseline for assessing relative enzyme activities across geographically diverse SDPs (Sinsabaugh et al., 2008; Wallenstein & Weintraub, 2008). However, incubation at a standardised temperature may also influence assay outcomes, potentially yielding values that differ from those obtained under *in situ* thermal conditions and thereby biasing observed biogeographic patterns. To address this potential limitation and assess whether assay temperature obscures enzymatic biogeographic signals, we complemented our analysis by quantifying enzyme activities at different, field-relevant temperatures using samples from the reciprocal transplant experiment. Each sample was incubated at the mean summer temperature of its receptor site as well as at the mean summer temperatures of the other recipient sites. This approach resulted in three incubation temperatures (8°C, 16°C and 20°C), as the Polish, Estonian and Finnish sites exhibited similar mean summer temperatures.

After incubation, fluorescence was measured spectrophotometrically (excitation: 365 nm, emission: 450 nm; Biotek SynergyMX). Enzyme activities were calculated by subtracting the mean fluorescence of the control wells from the mean fluorescence of the sample wells. Standard curves were generated using 7-amino-4-methylcoumarin (MUC) or methylumbelliferone (MUB) dissolved in phosphate buffer. Enzyme activities were expressed as nanomoles of substrate degraded (MUB or MUC) per gram dry mass per hour (nmol $g^{-1}$ DM $h^{-1}$).

## Data analyses

All data analyses were performed with R v4.3.3(R Core Team, 2023) and RStudio v0.9.1+401(Posit Team, 2025) using specific packages, as indicated afterwards. All visualisations were produced using the *ggplot2* package (Wickham, 2016).

**Collinearity among environmental predictors.** To address collinearity among environmental variables, we employed a systematic approach. First, we compared monthly and long-term variables by constructing a correlation matrix. Given the strong correlation observed between the two sets ($r > 0.8$ for all variables), we retained only the long-term variables for further analyses. Next, we performed a principal component analysis (PCA) on the preselected environmental variables. After visually identifying the main gradients, we conducted a second PCA on the selected variables to further reduce collinearity while keeping in mind the ecological logic specific to northern peatlands. Specifically, we aimed to i) avoid strong structural

correlations between highly related variables (*e.g.*, minimum and maximum temperatures), ii) retain the most ecologically significant SDP variables such as snow cover, precipitation, and soil moisture, iii) include local variables with direct relevance for microbial communities (*e.g.*, elevation, soil pH) (Delgado Baquerizo et al., 2018; Fierer & Jackson, 2006), and iv) capture aspects of ecosystem functioning (*e.g.*, gross primary productivity, GPP) (Jassey, Walcker, et al., 2022). Finally, we applied a clustering of variables approach (ClustOfVar) (Chavent et al., 2012) to validate the robustness of the identified main gradients, ensuring consistency in variable grouping and gradient interpretation from the PCA (Fig. 1c, Fig. S4).

**Bioclimatic effects by LMs.** Linear models (LMs) were used to assess the variability of bacterial features and enzymatic activities across the three bioclimatic regions (qualitative variables). The model was fit using the *lm()* function in the R environment. Prior to formal testing, the distribution of variables was visually inspected and log- or square-root transformed when necessary. The model assumptions of residual normality and homogeneity of variances were assessed using visual diagnostic plots (Q-Q plots and Residuals vs. Fitted plots, respectively). Due to the unequal sample sizes across bioclimatic regions (Fig. 1), the effect of the factor was tested using an Analysis of Variance (ANOVA) with Type III sums of squares. This approach ensures that the factor effect is assessed independently of the presence of other terms. Though not strictly necessary in a one-way design, it aligns with best practices for unbalanced data. The Type III ANOVA was performed using the *Anova()* function from the R package *car* (v3.1-3). Post-hoc pairwise comparisons were conducted to determine which specific groups differed from one another. Pairwise mean comparisons were performed using Tukey's Honest Significant Difference (HSD) test.

**Gamma and alpha diversity**. Rank-abundance curves were computed for each SDP's bioclimatic area. Patterns of gamma diversity were explored by calculating both observed and estimated (asymptotic) ASV richness using iNEXT (Hsieh et al., 2016). For alpha diversity, we applied the Hill numbers framework, which enables effective comparison of diversity indices based on the "equivalent number" of features (Jost, 2006). Accordingly, we report observed ASV richness (q = 0) and the effective number of ASVs derived from the exponential of Shannon entropy (q = 1), calculated using the function hill_taxa in the *R* package hillR(Li, 2018) (v.0.5.2). To evaluate the effects of environmental parameters on ASV richness, we fitted generalised linear models. Assumptions of normality and homoscedasticity were visually assessed using model residuals.

**Specific, unique and core ASVs**. Following Ezzat et al. (2025), we classified ASVs based on their distribution and relative abundance. ASVs detected exclusively in a single bioclimatic area, but across multiple SDPs of that particular bioclimatic area, were classified as specific, whereas those found in only two SDPs within a particular bioclimatic area were classified as unique. ASVs present in more than nine SDPs (*i.e.*, 5% of the SDPs) and detected at a relative abundance ≥0.1% were classified as widespread

ASVs. To visualise the relative abundance of the most abundant phyla and families across core, specific, and unique ASVs, we further plotted the mean non-null relative abundance across samples for each ASV against their prevalence in our dataset.

**Beta diversity.** To evaluate differences in community composition, we performed Principal Coordinate Analysis (PCoA) on Hellinger-transformed ASV abundances, which minimises the influence of dominant species on site dissimilarity (Legendre & Gallagher, 2001). Pairwise site dissimilarities were calculated using the Morisita-Horn distance within the phyloseq package(McMurdie & Holmes, 2013). We then applied Generalised Dissimilarity Modelling (GDM) (Ferrier et al., 2007) to quantify how environmental gradients and geographic distance drive ASV turnover among SDPs. GDM is a nonlinear extension of matrix regression that models the relationship between compositional dissimilarity and environmental or spatial predictors, allowing each predictor to contribute flexibly to the overall turnover pattern. The ASV matrix (reads per sample) was Hellinger-transformed, and environmental predictors were standardised prior to analysis. Collinearity among predictors was assessed to select a non-redundant set (see details above). Geographic distance between sites was also included as a predictor to account for spatial effects. Pairwise ASV dissimilarities were calculated using both Morisita-Horn and Jaccard distances to capture differences in abundance-weighted and presence-absence patterns, respectively. Variable significance in the GDM was evaluated via matrix permutations, comparing models with each variable permuted versus unpermuted. At each step, the least important variables were sequentially removed until only significant predictors remained. The final GDM was then refitted using only significant environmental variables, and model significance was verified and its deviance (*i.e.*, the variance explained by the model) calculated. Finally, variance partitioning was performed based on the contribution of each predictor to the total deviance explained by the final GDM model.

**Deterministic and stochastic community assembly processes**. Environmental filtering is a fundamental driver of community assembly, yet distinguishing its influence from that of historical and stochastic processes is often challenging. To evaluate the respective roles of deterministic and stochastic processes in shaping bacterial community assembly, we adopted the two-step framework detailed in Riddley et al. (2025), which is based on calculating the beta Nearest Taxon Index (βNTI) and the abundance-weighted Raup–Crick metric (RC_bray). Using this framework, we quantified the importance of ecological processes for all ASVs as well as for each bioclimatic zone. We also tested for correlations between ecological processes and the main environmental variables using modified Mantel tests (Riddley et al., 2025).

Using data from the reciprocal transplant experiment, we further explored the importance of environmental filtering on bacterial communities. We tested whether transplanted communities become more compositionally similar to receptor site communities than to their site of origin. To this end, we applied two complementary analytical approaches. First, we calculated pairwise community dissimilarities between transplanted communities and (i) their original site communities and (ii) receptor

site communities. Dissimilarities were computed using Morista-Horn's dissimilarity index to account for changes in relative abundance and Jaccard's dissimilarity index to capture presence–absence patterns, thereby disentangling abundance-driven shifts from compositional turnover. Second, we conducted Principal Coordinates Analysis (PCoA) based on Morista-Horn and Jaccard dissimilarity matrices and quantified community divergence in ordination space. For each analysis, we calculated Euclidean distances between centroids representing transplanted communities and centroids of both original and receptor site communities. Distances were computed using the PCoA axes collectively explaining 80% of the total variation in community composition. By comparing distances between transplanted communities and their original versus receptor sites across both dissimilarity-based and ordination-based approaches, we evaluated whether transplanted bacterial communities retained signatures of their origin or converged toward local receptor communities, providing insight into the strength of environmental filtering during community assembly. These comparisons were performed using ANOVAs.

**Drivers of enzymatic activities.** To evaluate the effects of environmental parameters on enzyme activities, we fitted generalised linear models. Assumptions of normality and homoscedasticity were visually assessed using model residuals. We identified the variables of importance by quantifying the strength and significance of the relationships by calculating Spearman's rank correlation coefficients and their associated *P*-values between enzymes and every environmental factor. We further investigated the specific linkages between each enzyme and the relative abundance of each ASV using the Microbiome Multivariate Associations with Linear Models framework (MasAsLin2) (Mallick et al., 2021). We applied a Benjamin-Hochberg FDR correction across all tests and addressed the compositional constraints of amplicon data via log-transformation. Effect sizes of the linear relationship were represented by the $\beta$ coefficients in the LMs.

Enzyme activities can be influenced by multiple, interconnected biotic and environmental mechanisms that operate simultaneously. To capture these complex relationships, we applied structural equation modelling (J B Grace et al., 2014) (SEM). Based on current knowledge of the potential direct and indirect drivers of enzyme activities (Fanin et al., 2022), we constructed an *a priori* path diagram (Fig. S5, Table S4) that allowed causal interpretation of the linkages among environmental factors, bacterial richness and community composition, and overall extracellular enzyme activity (EEA) (James B Grace & Keeley, 2006). While the overall model structure was theory-driven, the inclusion of environmental variables was, however, evaluated empirically among a set of pre-identified environmental covariates to address potential collinearity and avoid overfitting (James B Grace et al., 2016). All combinations among environmental covariates were evaluated, and the model with the best fit was selected based on fit indices and Akaike Information Criterion (AIC). The optimal SEM model included minimum temperature and pH as environmental covariates (Table S5). Our SEM model also included two latent constructs: Community Composition, defined by PCoA axes, and EEA, defined by the combined activity of each enzyme. By capturing the shared variance among the four enzymes, the EEA latent variable reflects underlying

microbial resource acquisition and nutrient cycling. Consequently, larger EEA values indicate greater overall enzymatic activity, although not necessarily uniformly across all enzymes. To assess the robustness of the relationships identified with the EEA latent variable, we also fitted separate SEMs for each enzyme. All models were fitted using the *lavaan* (Rosseel, 2012) (v.0.6-19) *R* package. Model fit adequacy was evaluated using $\chi^2$ tests ($P$-value > 0.05) and Akaike Information Criteria (AIC). Additional model fit was assessed with a low root-mean-square error of approximation index (RMSEA ≤ 0.1), low standardised root-mean-square residual index (SRMR ≤ 0.1) and high comparative fit index (CFI ≥ 0.95).

**Identifying critical thresholds in enzyme activities.** In an attempt to identify the critical threshold at which peatland functions begin to change alongside bacterial diversity loss, we employed smoothed structural equation modelling (sSEM) (Jassey et al., 2018). The model tested whether environmentally driven declines in bacterial diversity influence enzyme activities through changes in bacterial community structure and cell density. Smoothing was achieved by running moving-window SEMs along the bacterial richness continuum using the same SEM model. To construct the moving windows, we first ranked all SDPs from lowest to highest bacterial richness. At each step, we sequentially updated the subset of SDPs (*i.e.*, the window) by adding the next richest SDP and removing the least rich one. We initially evaluated window sizes ranging from 40 to 100 samples. Window sizes were first screened based on model convergence and the absence of issues with estimated variances, which reduced the range of viable window sizes to 75–100 samples. We then assessed SEM model fit based on model $P$-values, identifying window sizes of 85–100 as suitable. Finally, we compared the resulting trends in enzyme activity along the bacterial richness gradient across window sizes. Although all window sizes produced similar overall patterns (Fig. S6), windows of >95 samples resulted in excessive smoothing that obscured distinct regime shifts. We therefore selected a window size of 85 samples, which preserved the main regime-shift patterns while providing a larger number of overlapping windows across the richness gradient. This resulted in 32 overlapping transitions across the bacterial richness gradient.

For each window, we quantified EEA as the estimated score of the latent variable in the SEM, extracted using the *lavPredict()* function. Since this function returns factor scores with distribution centred near 0 and standardised to unit variance (so, with a mean = 0 for each window), we summarised enzyme multifunctionality by the 75$^{th}$ percentile of predicted latent scores in each window. This provided a robust measure of the upper-tail expression of EEA, identifying where it peaked across the bacterial richness gradient. To support this choice, we additionally calculated the standard deviation and maximum of latent scores per window, as well as a multifunctionality index using the average and multiple-threshold approach (Byrnes et al., 2014). Similar relationships were observed between bacterial richness and both the complementary multifunctionality metrics and the 75$^{th}$ percentile of the latent variable in SEM (Fig. S7). This confirms that our latent variable captured both the intensity and the variability of EEA along the gradient. Finally, we interrogated our SEM by performing a null model analysis to confirm that the latent variable was not simply the product of random associations between enzymes and bacterial community

composition. This was achieved by performing 1,000 randomisations of the entire dataset to break any structure in the data, rerunning smoothed SEM models with randomised data, extracting standardised effect sizes (Fisher-z based on Pearson's correlation) of the relationship between enzyme multifunctionality (latent variable, 75$^{th}$ percentile) and ASV richness and comparing the resulting associations to non-randomised standardised effect sizes (Fig. S8).

To identify thresholds in the response of EEA to declining bacterial richness, we used a Generalised Additive Model (GAM) to test the non-linear relationship between EEA and bacterial richness (*mgvc* R package), whilst broken-line regression models (*segmented R* package method) were used to identify possible tipping points in the response of EEA to bacterial biodiversity loss (Vanacker et al., 2015). To further assess the robustness of the identified tipping point, we calculated confidence intervals for tipping-point locations across window sizes of 85–95 samples, which confirmed the stability of the estimated tipping point across the range of suitable window sizes.

**References**

Byrnes, J. E. K., Gamfeldt, L., Isbell, F., Lefcheck, J. S., Griffin, J. N., Hector, A., Cardinale, B. J., Hooper, D. U., Dee, L. E., & Emmett Duffy, J. (2014). Investigating the relationship between biodiversity and ecosystem multifunctionality: challenges and solutions. *Methods in Ecology and Evolution*, *5*(2), 111–124. https://doi.org/10.1111/2041-210X.12143

Chavent, M., Kuentz-Simonet, V., Liquet, B., & Saracco, J. (2012). ClustOfVar: An R package for the clustering of variables. *Journal of Statistical Software*, *50*(13), 1–16. https://doi.org/10.18637/jss.v050.i13

Criquet, S., Tagger, S., Vogt, G., & Iacazio, G. (1999). Laccase activity of forest litter. *Soil Biology and Biochemistry*. http://www.sciencedirect.com/science/article/pii/S0038071799000383

Delgado Baquerizo, M., Oliverio, A. M., Brewer, T. E., Benavent-Gonzalez, A., Eldridge, D. J., Bardgett, R. D., Maestre, F. T., Singh, B. K., & Fierer, N. (2018). A global atlas of the dominant bacteria found in soil. *Science*, *359*(6373), 320–325. http://www.sciencemag.org/lookup/doi/10.1126/science.aap9516

Douglas, G. M., Maffei, V. J., Zaneveld, J. R., Yurgel, S. N., Brown, J. R., Taylor, C. M., Huttenhower, C., & Langille, M. G. I. (2020). PICRUSt2 for prediction of metagenome functions. *Nature Biotechnology 2020 38:6*, *38*(6), 685–688. https://doi.org/10.1038/s41587-020-0548-6

Ezzat, L., Peter, H., Bourquin, M., Busi, S. B., Michoud, G., Fodelianakis, S., Kohler, T. J., Lamy, T., Geers, A., Pramateftaki, P., Baier, F., Marasco, R., Daffonchio, D., Deluigi, N., Wilmes, P., Styllas, M., Schön, M., Tolosano, M., De Staercke, V., & Battin, T. J. (2025). Diversity and biogeography of the bacterial microbiome in glacier-fed streams. *Nature*, *637*, 622–630. https://doi.org/10.1038/s41586-024-08313-z

Fanin, N., Mooshammer, M., Sauvadet, M., Meng, C., Alvarez, G., Bernard, L., Bertrand, I., Blagodatskaya, E., Bon, L., Fontaine, S., Niu, S., Lashermes, G., Maxwell, T. L., Weintraub, M. N.,

Wingate, L., Moorhead, D., & Nottingham, A. T. (2022). Soil enzymes in response to climate warming: Mechanisms and feedbacks. *Functional Ecology*, *36*(6), 1378–1395. https://doi.org/10.1111/1365-2435.14027

Ferrier, S., Manion, G., Elith, J., & Richardson, K. (2007). Using Generalized Dissimilarity Modelling to Analyse and Predict Patterns of Beta Diversity in Regional Biodiversity Assessment. *Diversity and Distributions*, *13*(3), 252–264. papers3://publication/doi/10.2307/4539919?refreqid=search-gateway:976bd3bb75ba15ee80b27beb1be94bcb

Fierer, N., & Jackson, R. B. (2006). The diversity and biogeography of soil bacterial communities. *Proceedings of the National Academy of Sciences of the United States of America*, *103*(3), 626–631. http://eutils.ncbi.nlm.nih.gov/entrez/eutils/elink.fcgi?dbfrom=pubmed&id=16407148&retmode=ref&cmd=prlinks

Galen, L. G. Van. (2025). A global database of soil microbial phospholipid fatty acids and enzyme activities. *Scientific Data*, *12*, 1568.

Grace, J B, Adler, P. B., Harpole, W. S., Borer, E. T., & Seabloom, E. W. (2014). Causal networks clarify productivity–richness interrelations, bivariate plots do not. *Functional Ecology*, *28*(4), 787–798. http://onlinelibrary.wiley.com/doi/10.1111/1365-2435.12269/full

Grace, James B, Anderson, T. M., Seabloom, E. W., Borer, E. T., Adler, P. B., Harpole, W. S., Hautier, Y., Hillebrand, H., Lind, E. M., Pärtel, M., Bakker, J. D., Buckley, Y. M., Crawley, M. J., Damschen, E. I., Davies, K. F., Fay, P. A., Firn, J., Gruner, D. S., Hector, A., … Smith, M. D. (2016). Integrative modelling reveals mechanisms linking productivity and plant species richness. *Nature*, *529*(7586), 390–393. http://www.nature.com/doifinder/10.1038/nature16524

Grace, James B, & Keeley, J. E. (2006). A structural equation model analysis of postfire plant diversity in California shrublands. *Ecological Applications : A Publication of the Ecological Society of America*, *16*(2), 503–514. http://eutils.ncbi.nlm.nih.gov/entrez/eutils/elink.fcgi?dbfrom=pubmed&id=16711040&retmode=ref&cmd=prlinks

Hsieh, T. C., Ma, K. H., & Chao, A. (2016). iNEXT: an R package for rarefaction and extrapolation of species diversity ( <scp>H</scp> ill numbers). *Methods in Ecology and Evolution*, *7*(12), 1451–1456. https://doi.org/10.1111/2041-210X.12613

Jassey, V. E. J., Hamard, S., Lepère, C., Céréghino, R., Corbara, B., Küttim, M., Leflaive, J., Leroy, C., & Carrias, J.-F. (2022). Photosynthetic microorganisms effectively contribute to bryophyte CO2 fixation in boreal and tropical regions. *ISME Communications 2022 2:1*, *2*(1), 1–10. https://doi.org/10.1038/s43705-022-00149-w

Jassey, V. E. J., Reczuga, M. K., Zielińska, M., Słowińska, S., Robroek, B. J. M., Mariotte, P., Seppey, C. V. W., Lara, E., Barabach, J., Słowiński, M., Bragazza, L., Chojnicki, B. H., Lamentowicz, M., Mitchell, E. A. D., & Buttler, A. (2018). Tipping point in plant-fungal interactions under severe drought causes

abrupt rise in peatland ecosystem respiration. *Global Change Biology*, *24*(3), 972–986. http://eutils.ncbi.nlm.nih.gov/entrez/eutils/elink.fcgi?dbfrom=pubmed&id=28991408&retmode=ref&cmd=prlinks

Jassey, V. E. J., Walcker, R., Kardol, P., Geisen, S., Heger, T., Lamentowicz, M., Hamard, S., & Lara, E. (2022). Contribution of soil algae to the global carbon cycle. *New Phytologist*, *234*(1), 64–76. https://doi.org/10.1111/nph.17950

Jost, L. (2006). Entropy and diversity. *Oikos*, *113*(2), 363–375. https://doi.org/10.1111/j.2006.0030-1299.14714.x

Legendre, P., & Gallagher, E. D. (2001). Ecologically meaningful transformations for ordination of species data. *Oecologia*, *129*(2), 271–280. http://link.springer.com/article/10.1007/s004420100716

Li, D. (2018). hillR: taxonomic, functional, and phylogenetic diversity and similarity through Hill Numbers. *Journal of Open Source Software*, *3*(31), 1041. https://doi.org/10.21105/joss.01041

Liu, C., Cui, Y., Li, X., & Yao, M. (2021). microeco : an R package for data mining in microbial community ecology. *FEMS Microbiology Ecology*, *97*, fiaa255. https://doi.org/10.1093/femsec/fiaa255

Mallick, H., Rahnavard, A., McIver, L. J., Ma, S., Zhang, Y., Nguyen, L. H., Tickle, T. L., Weingart, G., Ren, B., Schwager, E. H., Chatterjee, S., Thompson, K. N., Wilkinson, J. E., Subramanian, A., Lu, Y., Waldron, L., Paulson, J. N., Franzosa, E. A., Bravo, H. C., & Huttenhower, C. (2021). Multivariable association discovery in population-scale meta-omics studies. *PLOS Computational Biology*, *17*, e1009442. https://doi.org/10.1371/journal.pcbi.1009442

McMurdie, P. J., & Holmes, S. (2013). phyloseq: An R Package for Reproducible Interactive Analysis and Graphics of Microbiome Census Data. *PLOS ONE*, *8*(4), e61217. https://doi.org/10.1371/JOURNAL.PONE.0061217

Posit Team. (2025). RStudio: Integrated Development Environment for R. *Posit Software, PBC, Boston, MA.* http://www.posit.co/.

Quast, C., Pruesse, E., Yilmaz, P., Gerken, J., Schweer, T., Yarza, P., Peplies, J., & Glöckner, F. O. (2013). The SILVA ribosomal RNA gene database project: improved data processing and web-based tools. *Nucleic Acids Research*, *41*(D1), D590–D596. https://doi.org/10.1093/NAR/GKS1219

R Core Team. (2023). *R: A language and environment for statistical computing. R Foundation for Statistical Computing, Vienna, Austria* (4.0.2).

Riddley, M., Hepp, S., Hardeep, F. N. U., Nayak, A., Liu, M., Xing, X., Zhang, H., & Liao, J. (2025). Differential roles of deterministic and stochastic processes in structuring soil bacterial ecotypes across terrestrial ecosystems. *Nature Communications 2025 16:1*, *16*(1), 1–13. https://doi.org/10.1038/s41467-025-57526-x

Rosseel, Y. (2012). lavaan: An R Package for Structural Equation Modeling. *Journal of Statistical Software*, *48*(2), 1–36. http://www.jstatsoft.org/v48/i02/

Sinsabaugh, R. L., Lauber, C. L., Weintraub, M. N., Ahmed, B., Allison, S. D., Crenshaw, C., Contosta, A.

R., Cusack, D., Frey, S., Gallo, M. E., Gartner, T. B., Hobbie, S. E., Holland, K., Keeler, B. L., Powers, J. S., Stursova, M., Takacs-Vesbach, C., Waldrop, M. P., Wallenstein, M. D., … Zeglin, L. H. (2008). Stoichiometry of soil enzyme activity at global scale. *Ecology Letters*, *11*(11), 1252–1264. https://doi.org/10.1111/j.1461-0248.2008.01245.x

Stilianos Louca, Parfrey, L. W., & Doebeli, M. (2016). Decoupling function and taxonomy in the global ocean microbiome. *Science*, *353*, 1272–1277. https://doi.org/10.1126/science.aaf4507

Vanacker, M., Wezel, A., Payet, V., & Robin, J. (2015). Determining tipping points in aquatic ecosystems: The case of biodiversity and chlorophyll α relations in fish pond systems. *Ecological Indicators*, *52*, 184–193. http://linkinghub.elsevier.com/retrieve/pii/S1470160X14005792

Wallenstein, M. D., & Weintraub, M. N. (2008). *Soil Biology & Biochemistry Emerging tools for measuring and modeling the in situ activity of soil extracellular enzymes*. *40*, 2098–2106. https://doi.org/10.1016/j.soilbio.2008.01.024

Weedon, J. T., Aerts, R., Kowalchuk, G. A., & van Bodegom, P. M. (2014). No effects of experimental warming but contrasting seasonal patterns for soil peptidase and glycosidase enzymes in a sub-arctic peat bog. *Biogeochemistry*, *117*(1), 55–66. http://link.springer.com/article/10.1007/s10533-013-9870-0/fulltext.html
