# Supplementary material for: Tipping Points in Bacterial Richness Amplify Extracellular Enzyme Activities in Northern Peatlands
Source: Glob Chang Biol. 2026 Sep 17;32(9):e71099. doi: 10.1111/gcb.71099 (PMC13586543; doi:10.1111/gcb.71099)

Supplementary figures

Tipping points in bacterial richness amplify extracellular enzyme activities in northern peatlands

Authors

Vincent E.J. Jassey, Tristan Lafont Rapnouil, *et al*[#].

[#]A full list of authors and their affiliations appears on the paper.

Contact : vincent.jassey@utoulouse.fr

Fig. S1. A comparison of the bacterial community structure within-site (different microhabitats) and between-sites (same microhabitat). The dissimilarity (based on Horn's distance) within and between sites is shown in **a**, the global survey, in which some extensive sites were sampled several times in different microhabitats, and **b**, using an independent data set from (Hamard et al., 2021). Asterisks indicate statistical differences at $P < 0.001$ (ANOVA, Type III error).

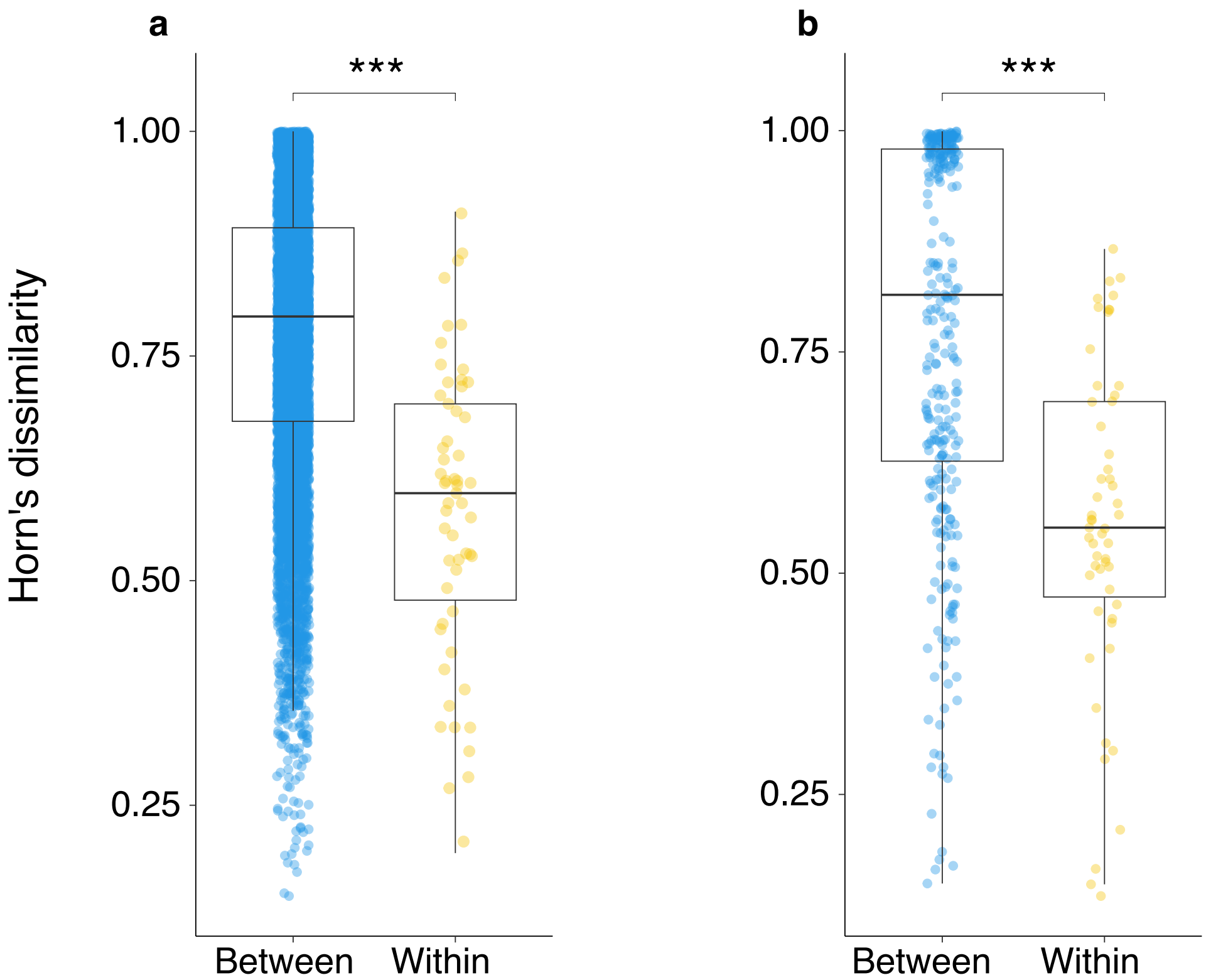

Fig. S2. Relationship between the pH measured in the field and the one retrieved from OpenLand. The line represent the linear predictor of the regression equation with its standard error (grey area) from ANOVAs. Spearman correlation coefficient (ρ) and P-values are also given on the biplot.

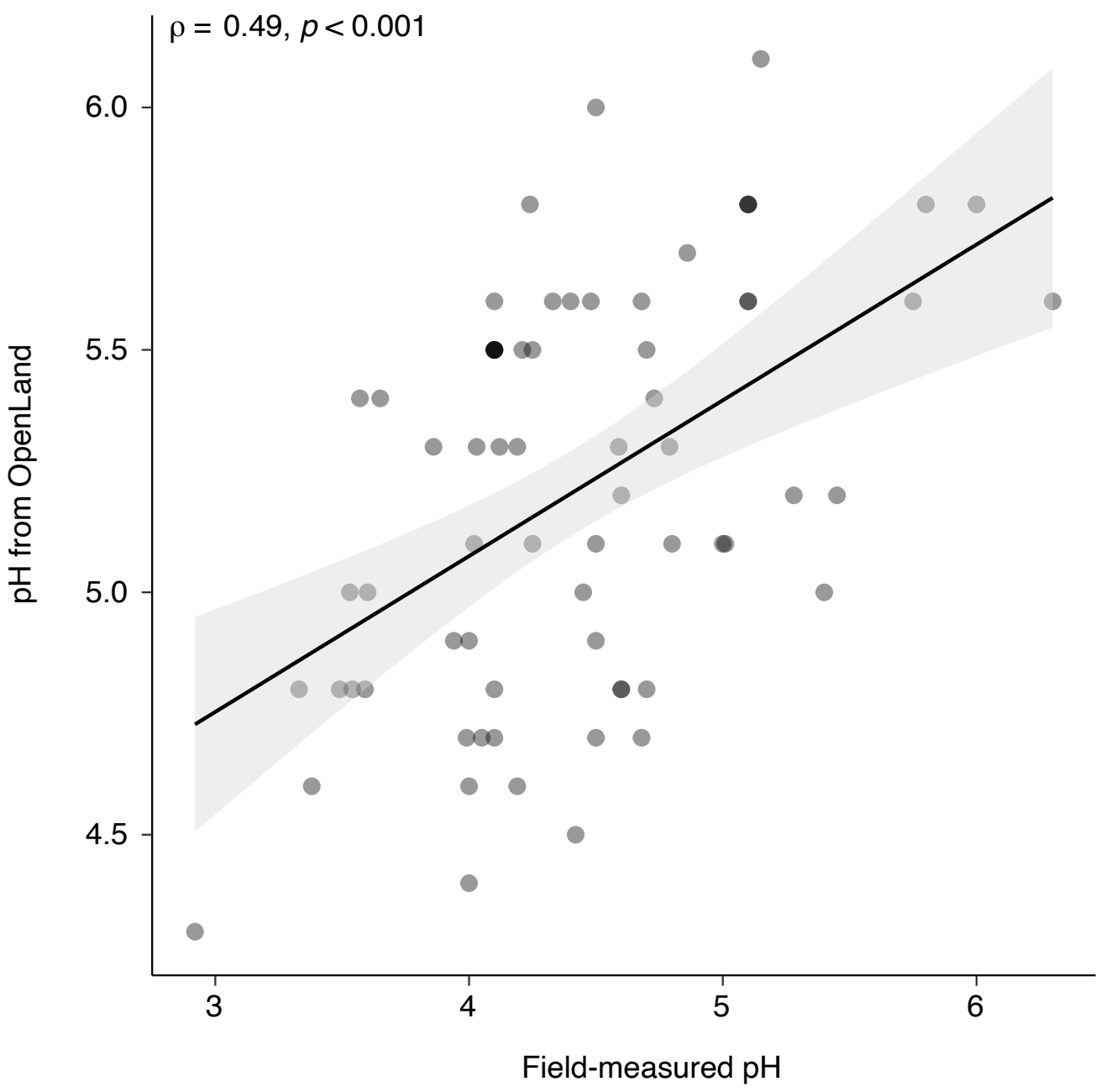

Fig. S3. Rarefaction curves of bacterial amplicon sequence variants (ASVs) for the 171 SDPs. Each curve represents one SDP sample. Colours indicate bioclimatic regions: red = Continental hot summer, green = Temperate, blue = Continental Cold/Tundra. Curves approaches an asymptote for all samples, indicating that the sequencing depth captured the majority of bacterial diversity.

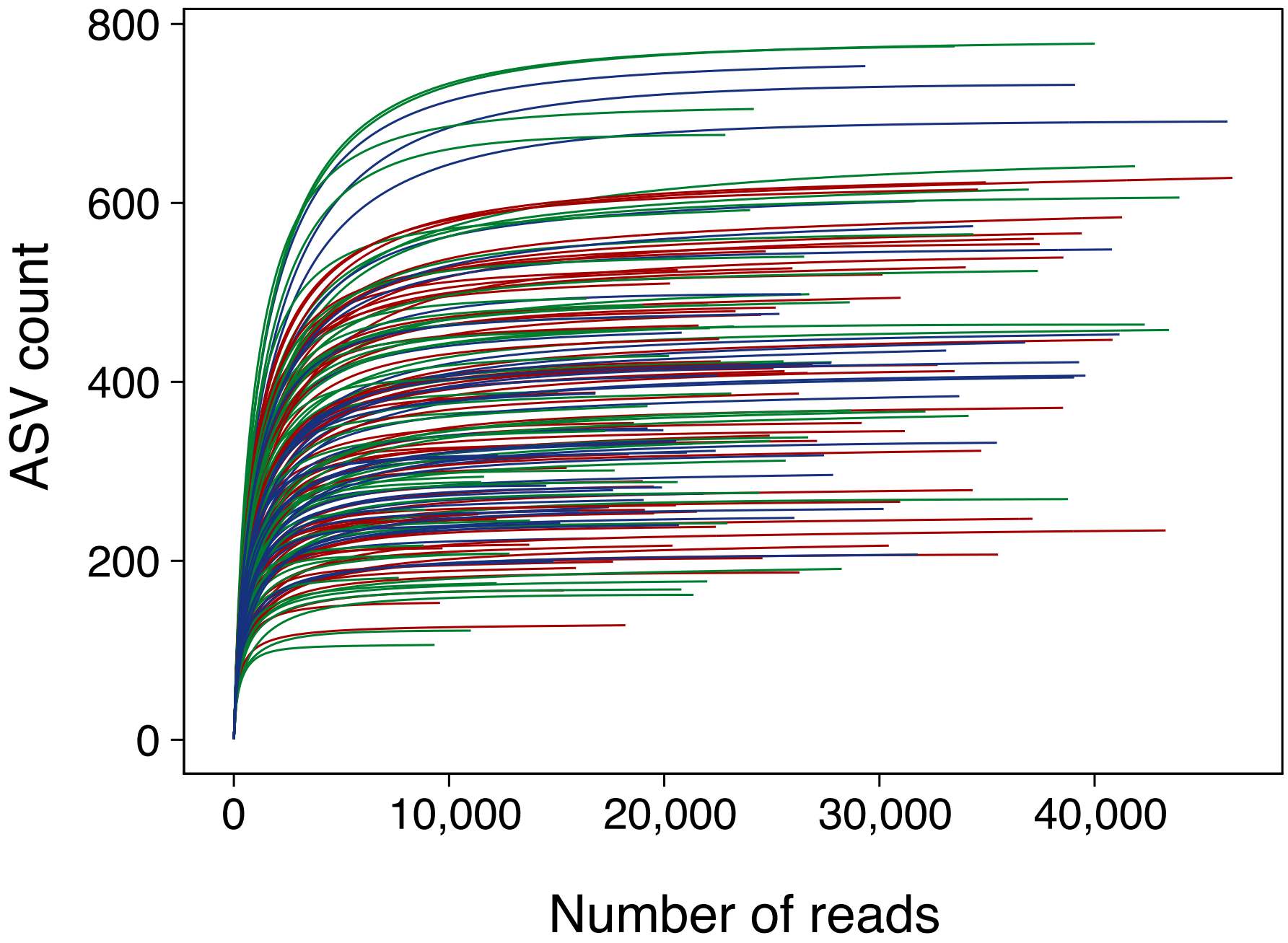

Fig. S4. Environmental variable selection. Dendrogram of hierarchical cluster analysis of quantitative predictors in a pre-selected dataset. Height is expressed as the aggregation level of the clusters. Colours characterize the six main clusters identified with ClustOfVar *R* package.

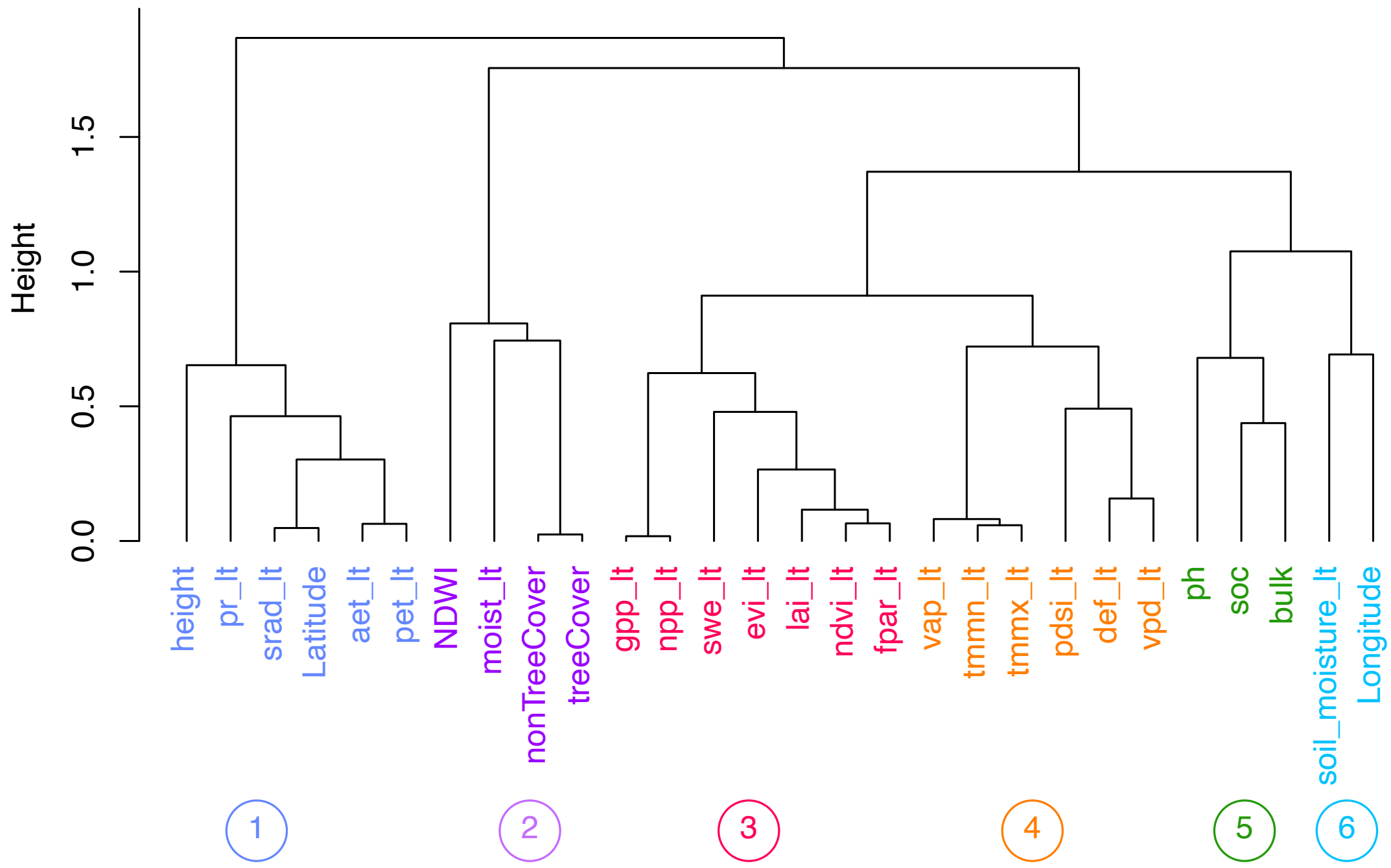

Fig. S5. A priori conceptual structural equation model (SEM). This a priori model illustrates how vegetation, climate, and soil properties may directly influence enzyme activities, as well as indirectly through their effects on bacterial community features, such as ASV richness, cell density, and community composition. For clarity, only global pathways by variable category are shown. Numbers correspond to hypothetical pathways described in Supplementary Table S4.

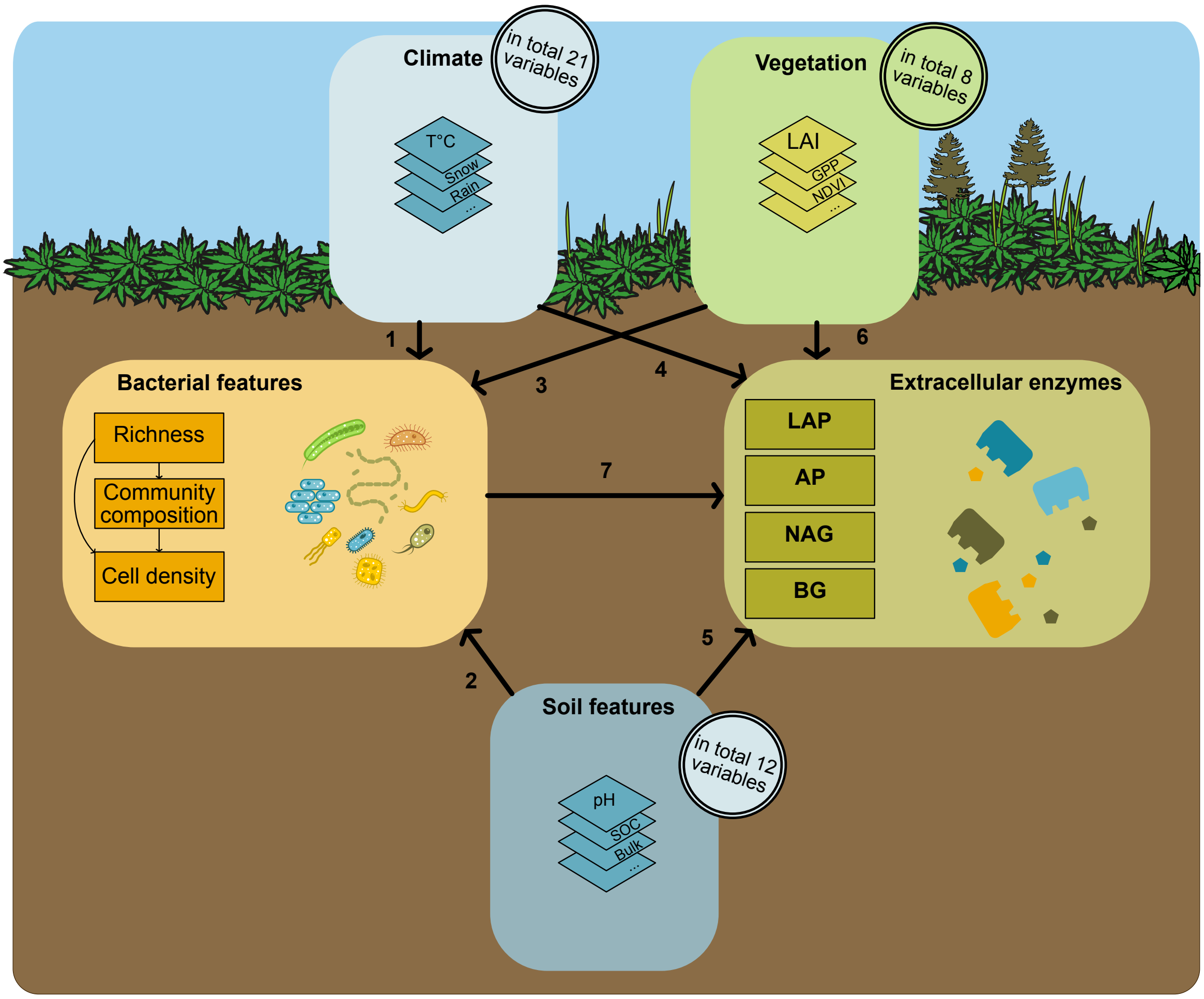

Fig. S6. Selection of the moving-window size for the smoothed SEM. (**a**) Number of SEMs with convergence or estimated variance issues (blue line, left axis) and number of SEMs with unsatisfactory model fit (red line, right axis) across moving-window sizes. Window sizes between 85 and 100 samples provided the best compromise between model convergence and fit. (**b**) Response of extracellular enzyme activity (EEA; 75th quartile of latent variable from the SEMs) to bacterial richness for representative moving-window sizes. Although all window sizes produced similar overall patterns, larger window sizes progressively smoothed the relationship, reducing the visibility of the transition around the bacterial richness threshold. Based on these results, a window size of 85 samples was selected for subsequent analyses.

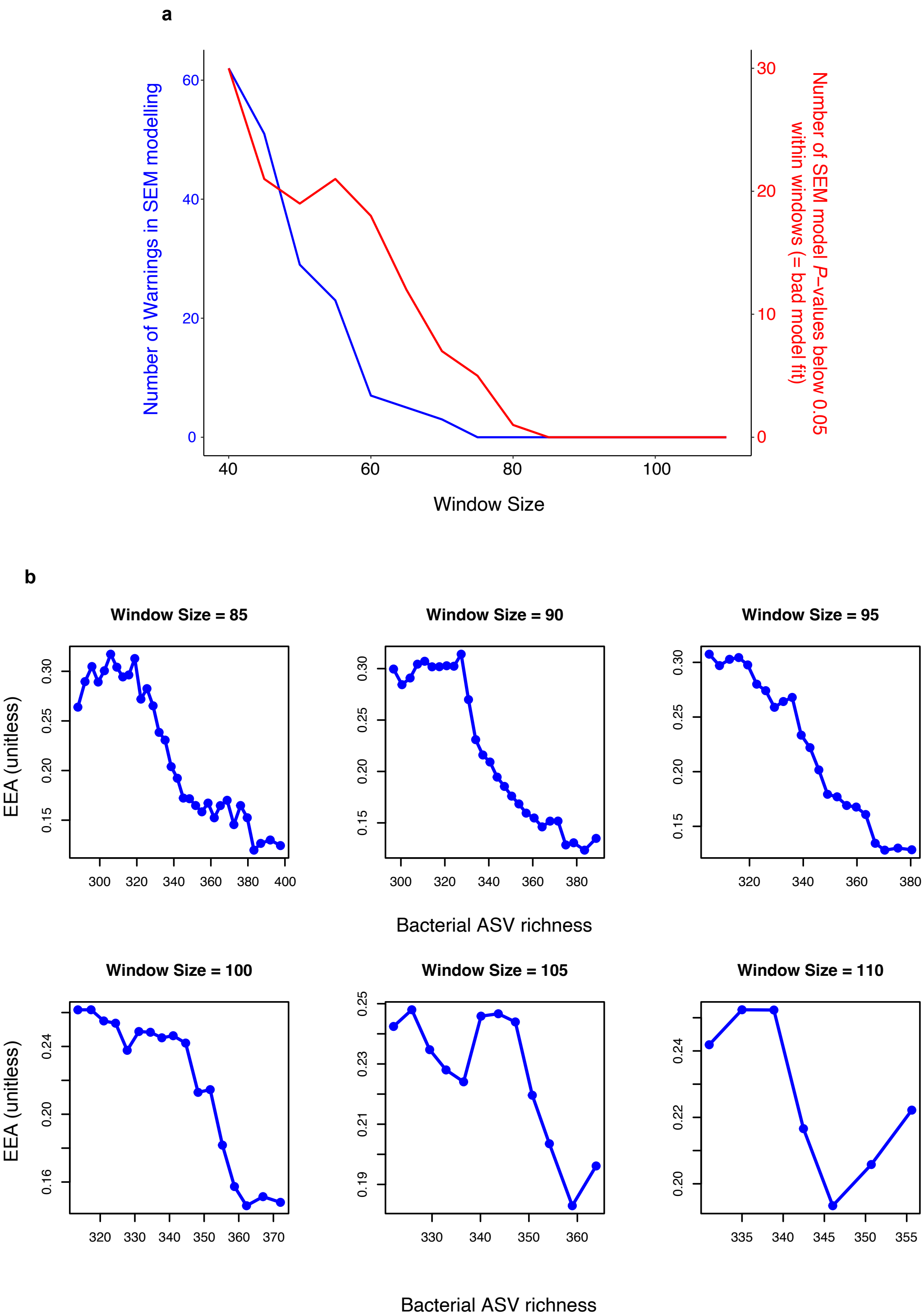

Fig. S7. Comparison of two approaches to quantify enzyme multifunctionality, and the latent SEM variable used here. Standardized effect sizes (SES) showing the strength of the response of enzyme multifunctionality to decreasing bacterial richness. The dots show the mean of SESs and the bars the standard error. The multifunctionality index based on the latent variable in SEM (75th percentile) is in grey, while approaches based averaging and multiple thresholds are in black. See Supplementary methods for details.

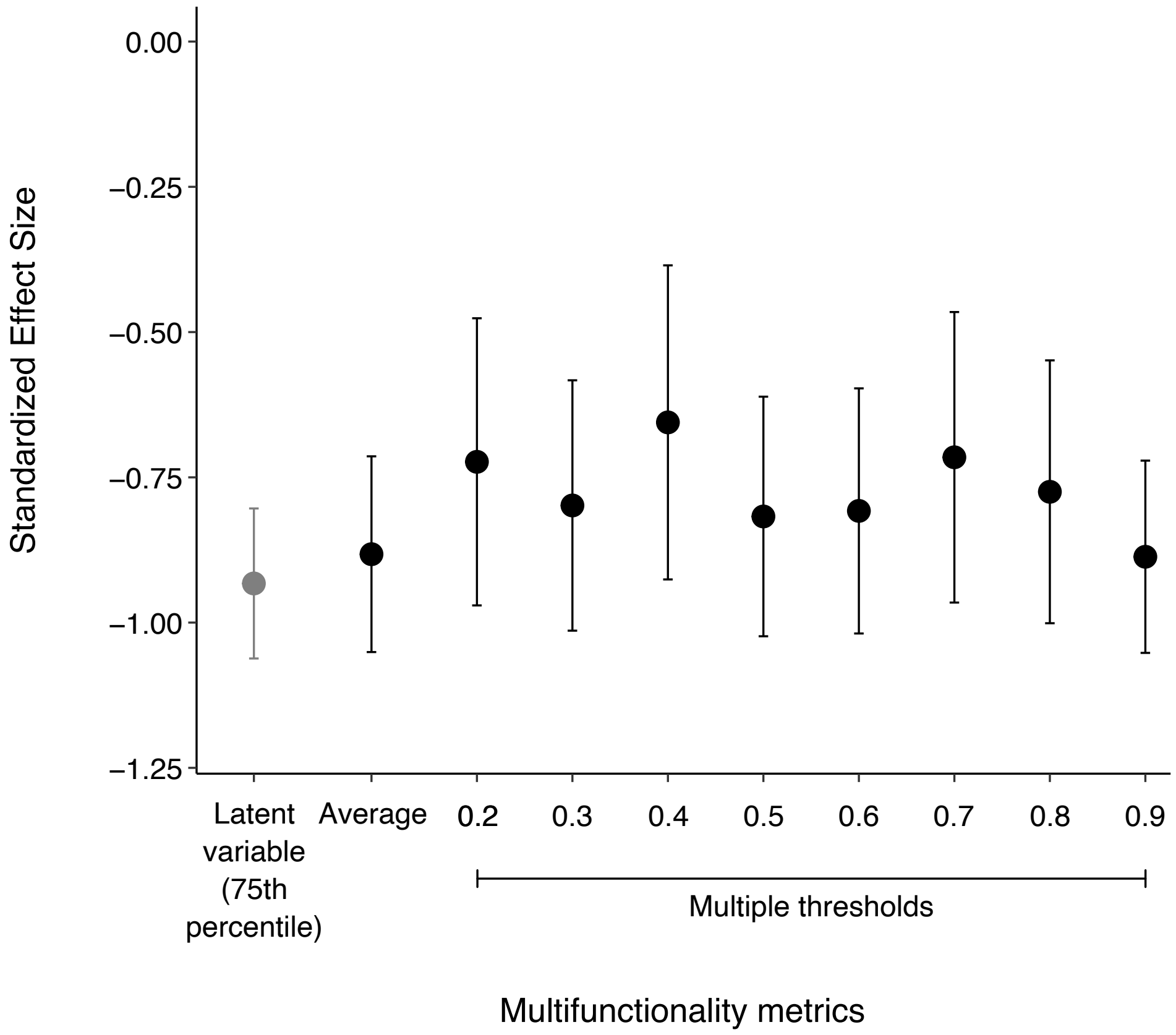

Fig. S8. Effect of randomization on the link between EEA (75th percentile of the latent variable) and bacterial ASV richness in smoothed SEMs. The response of enzyme multifunctionality to bacterial ASV richness in each randomization iteration (k = 1,000) is expressed as standardized effect size (fisher-z). Boxplot indicate the median (centre line), first and third quartiles (lower and upper box edges) and 1.5x the interquartile range (IQR) (box whiskers). The red dot shows the observed SES based on non-randomized data. See Supplementary methods for details.

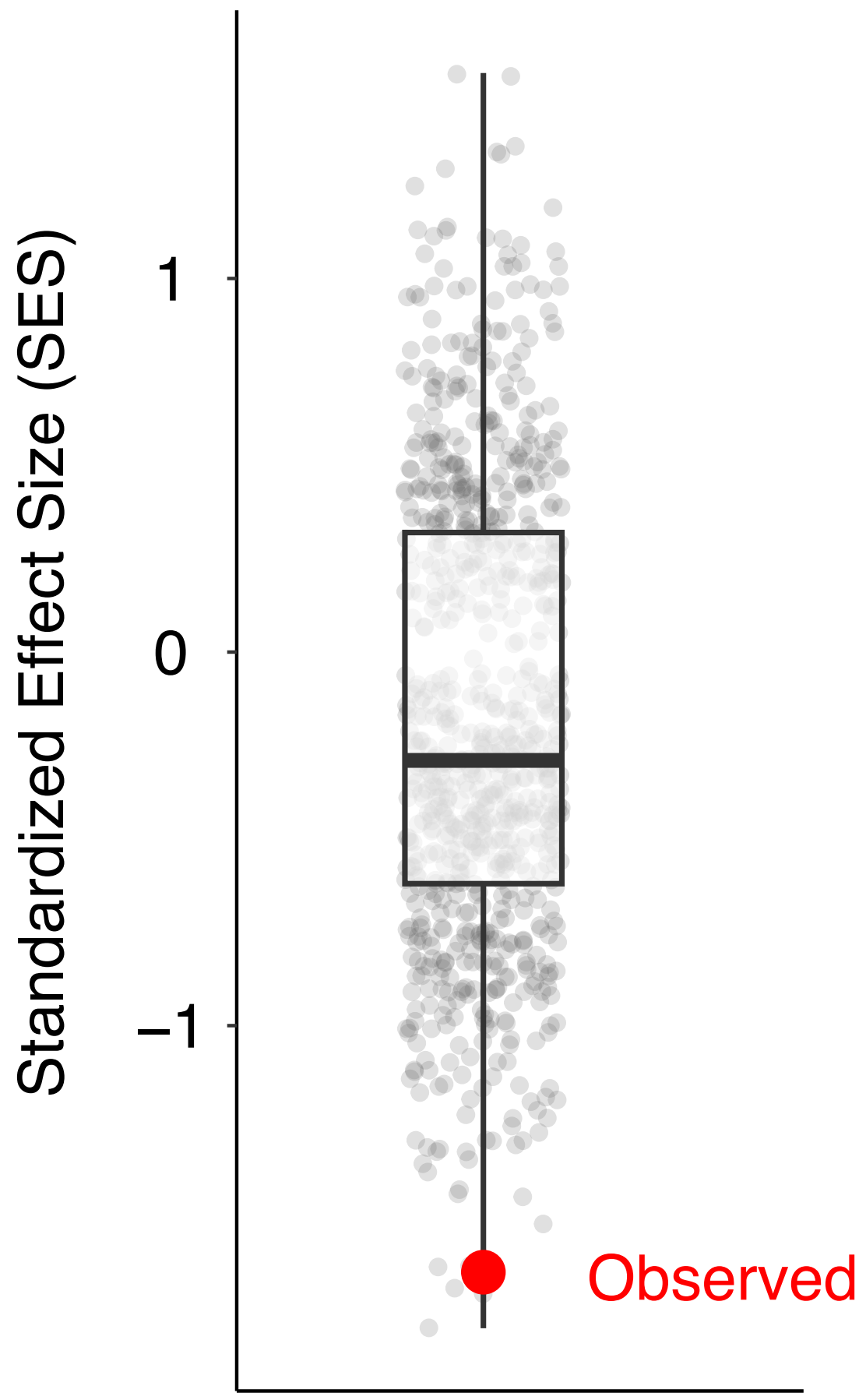

Fig. S9. Bacterial community composition. Relative abundance (%) of bacterial orders (left panel) and genus (right panel) retrieved in SDPs, grouped by bioclimatic area.

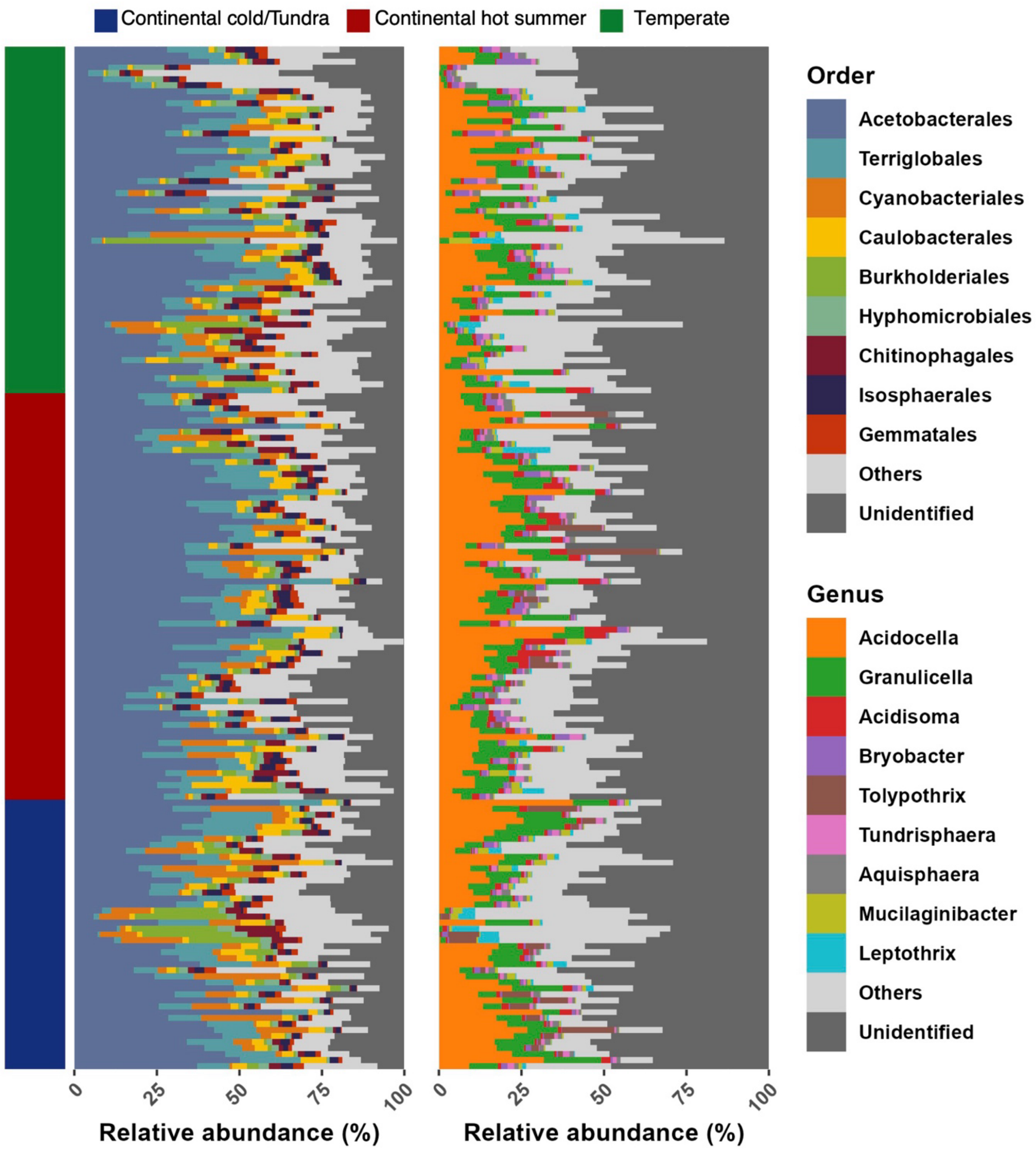

Fig. S10. Rank abundance model fits. Rank abundance curves (RAC) for the different SDP bioclimatic regions. Bold lines represent the mean RAC for a given bioclimatic area (n = 171 SDPs). Rank abundance distributions with log-normal model and Brokenstick null model fits can be found in Fig. S11.

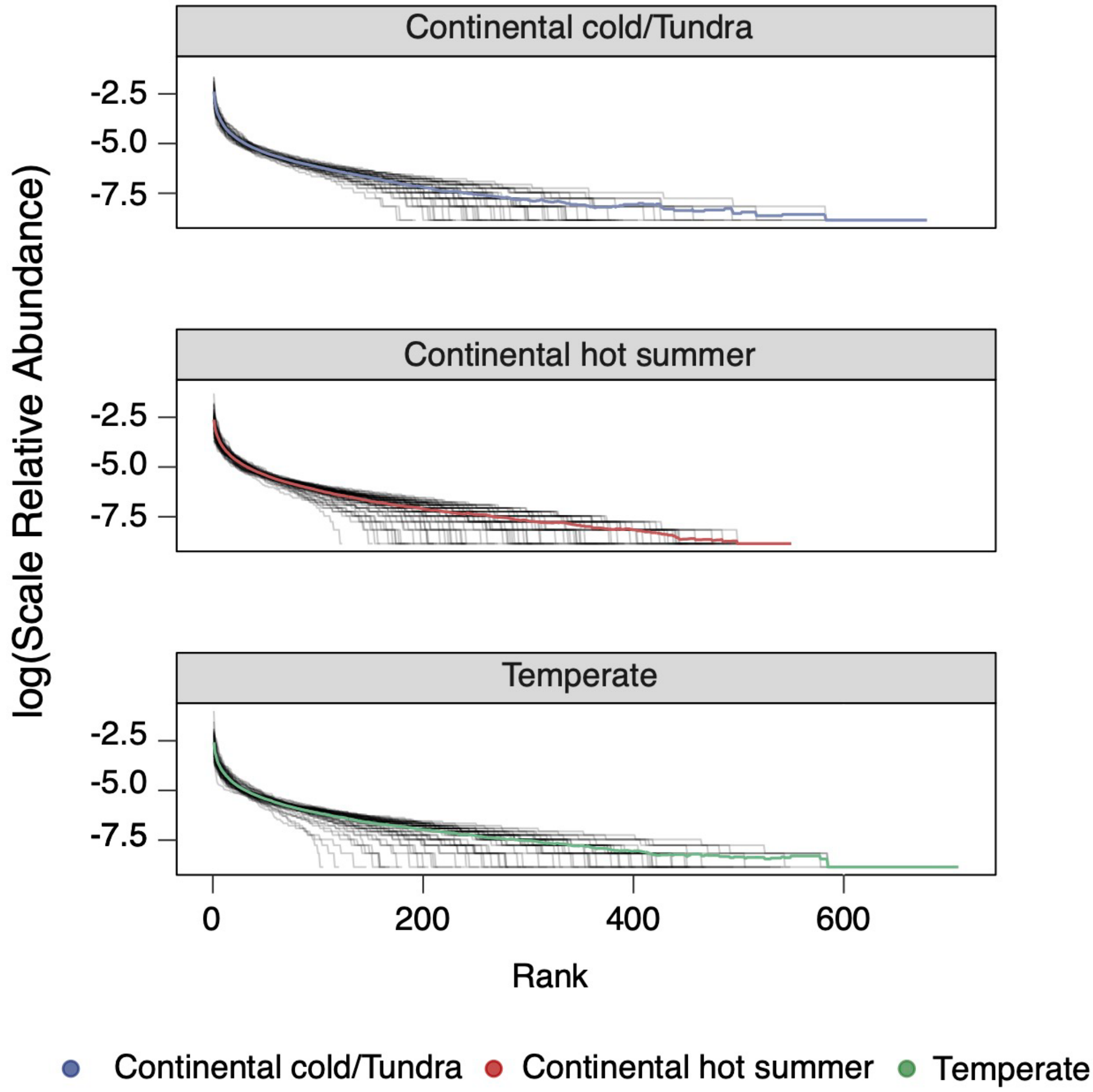

Fig. S11. **Rank-abundance model fits**. Rank-abundance distributions with log-normal model (red) and Brokenstick null model (cyan) fits for a subset of 28 SDPs. The entire set of distributions for both filtered-rarefied and unfiltered datasets is found on data repository (see Data Availability section). In most SDP communities, the log-normal model closely fits empirical abundance distributions (grey). This model, which becomes approximately normal after log-transformation, is expected for large communities due to the central limit theorem and likely emerges from stochastic processes like growth and dispersal.

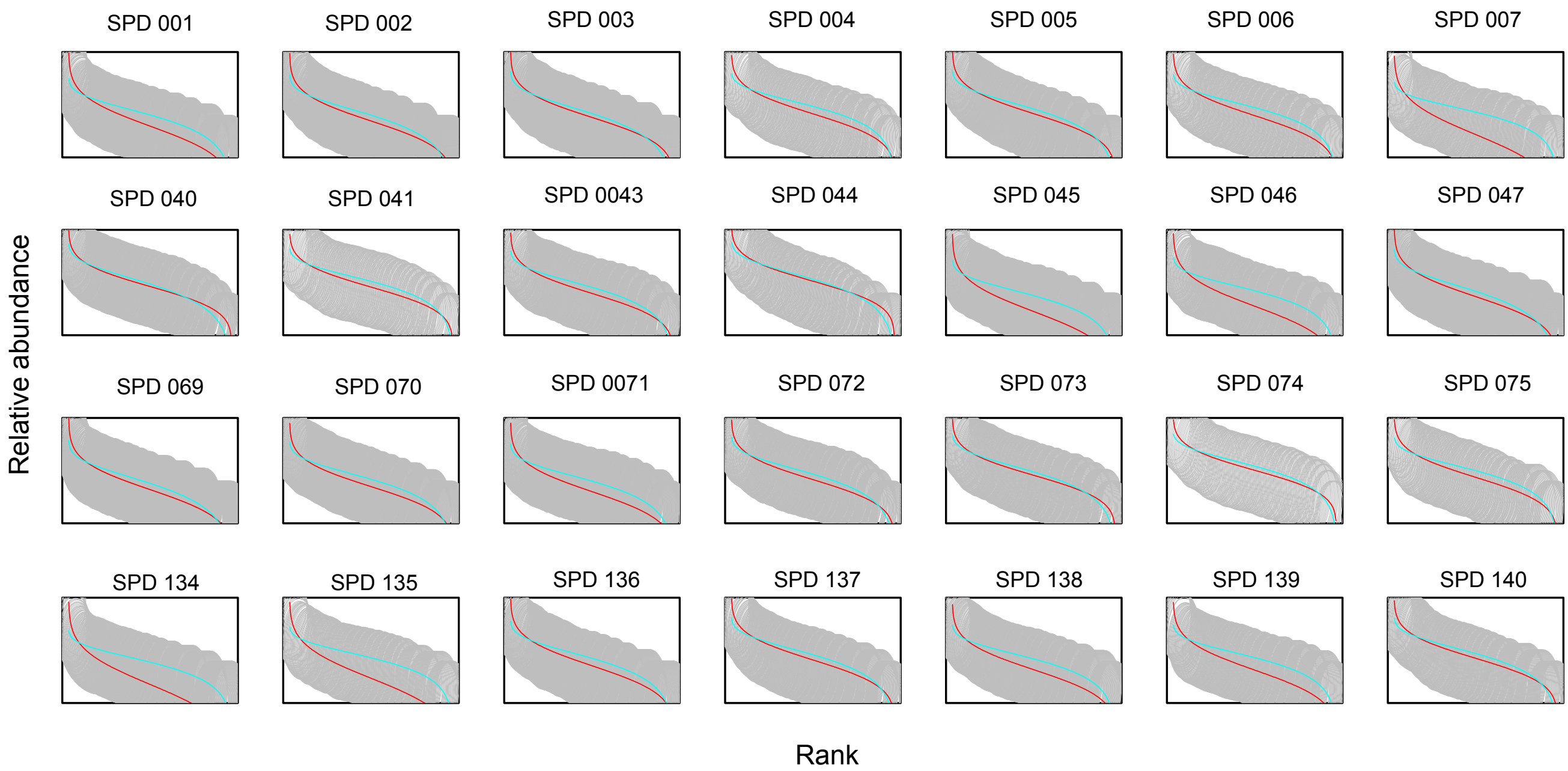

Fig. S12. Linkages between bacterial ASV richness and geographic and environmental parameters. The lines represent the linear predictor of the regression equation with its standard error (grey area) from ANOVAs (see Methods). Spearman correlation coefficient and *P*-values are also given on each biplot.

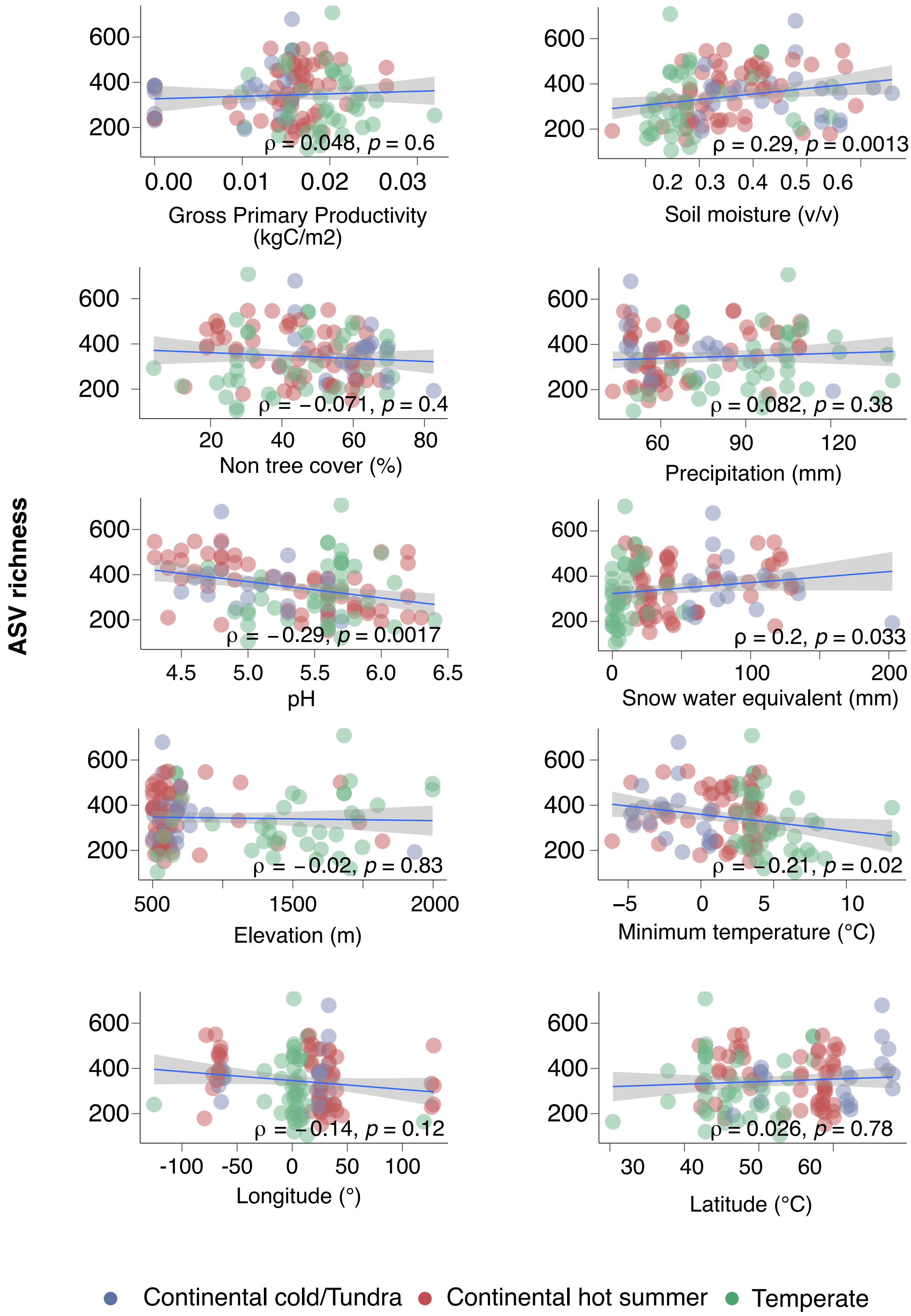

Fig. S13. Linkages between bacterial cell density and geographic and environmental parameters. The lines represent the linear predictor of the regression equation with its standard error (grey area) from ANOVAs (see Methods). Spearman correlation coefficient and *P*-values are also given on each biplot.

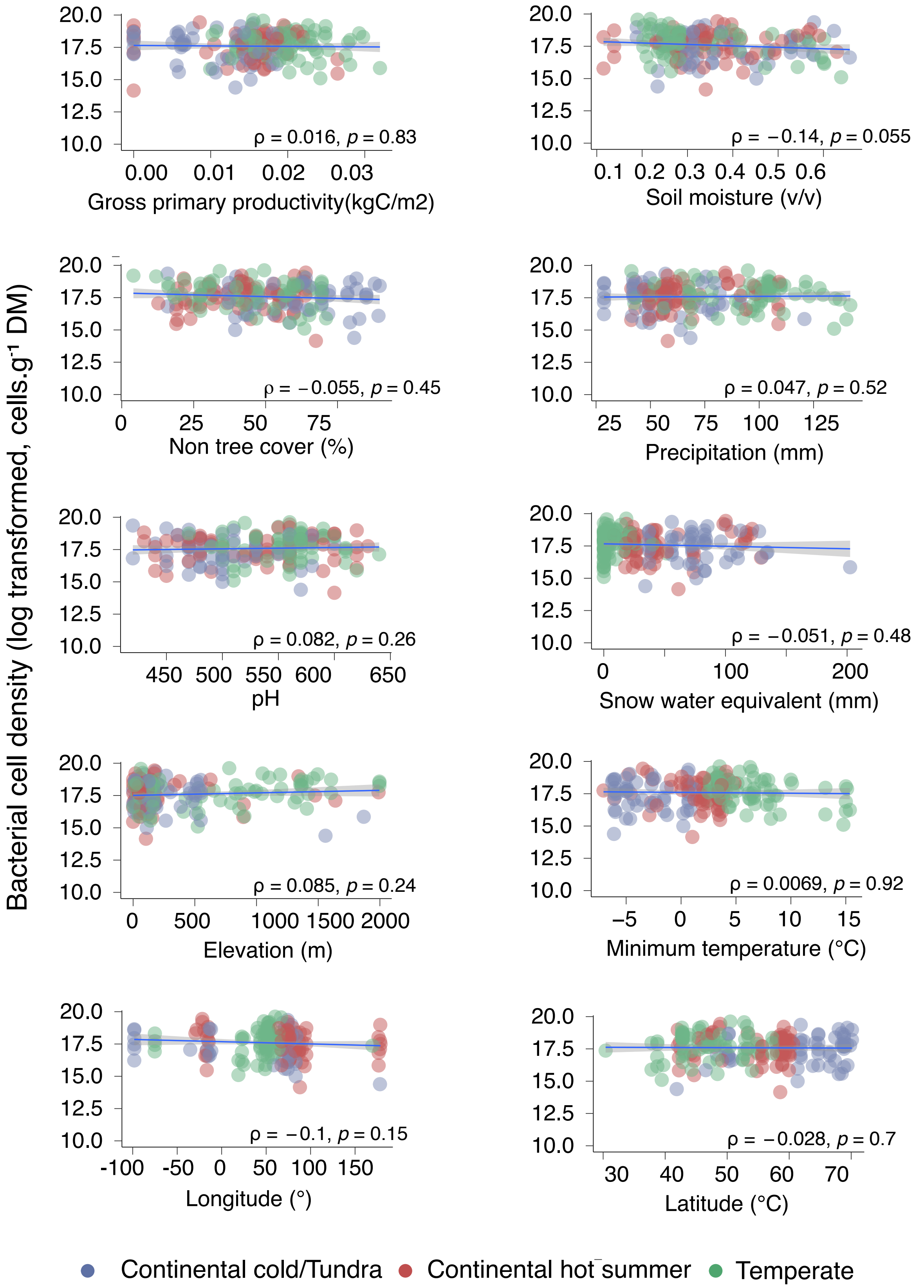

Fig. S14. **Taxonomic turnover along geographic distance**. **a**, Relationship between observed bacterial community compositional dissimilarity between site pairs (ASV relative abundance, β-diversity, Bray-Curtis distance) and their geographic distance. **b**, Relationship between observed bacterial community compositional dissimilarity between site pairs (ASV presence/absence, β-diversity, Jaccard distance) and their geographic distance. The lines represent the smoothed predictor of the regression equation from generalised dissimilarity modelling (GDM, see Methods). Dot colours show between (red) and within (blue) SDP bioclimatic region dissimilarity.

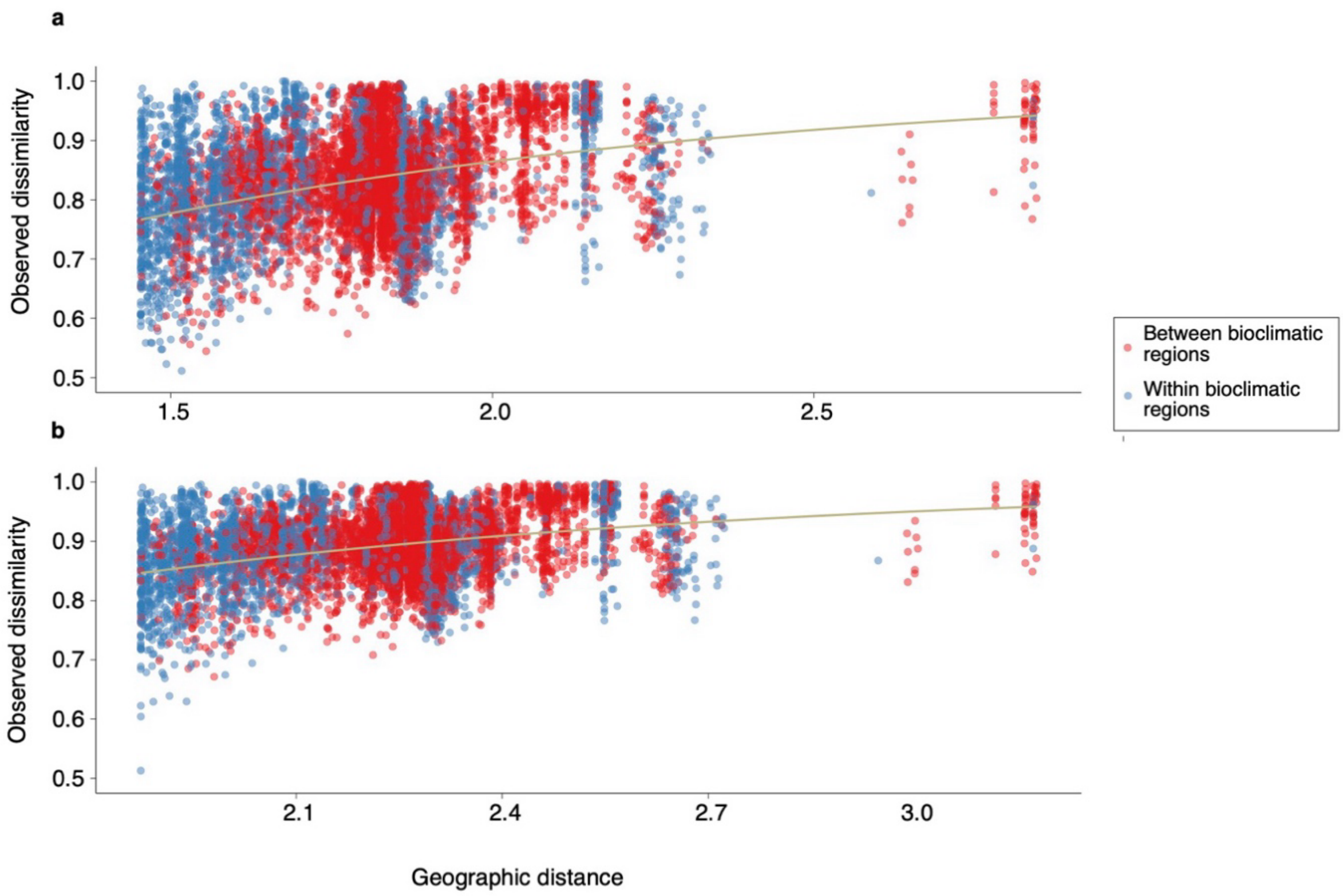

Figure S15. Components and taxonomy of the SDP microbiome. **a**, Distribution of global SDP microbiome genera according to their relative prevalence and abundance. Dot colour denotes genus. **b**, Distribution of specific ASVs (*i.e.*, occurrence in less than 5% of SDPs) across bioclimatic regions, shown as absolute numbers (*x* axis) and fraction (%) of ASVs per bioclimatic area. Blue and grey correspond to unique and other ASVs, respectively.

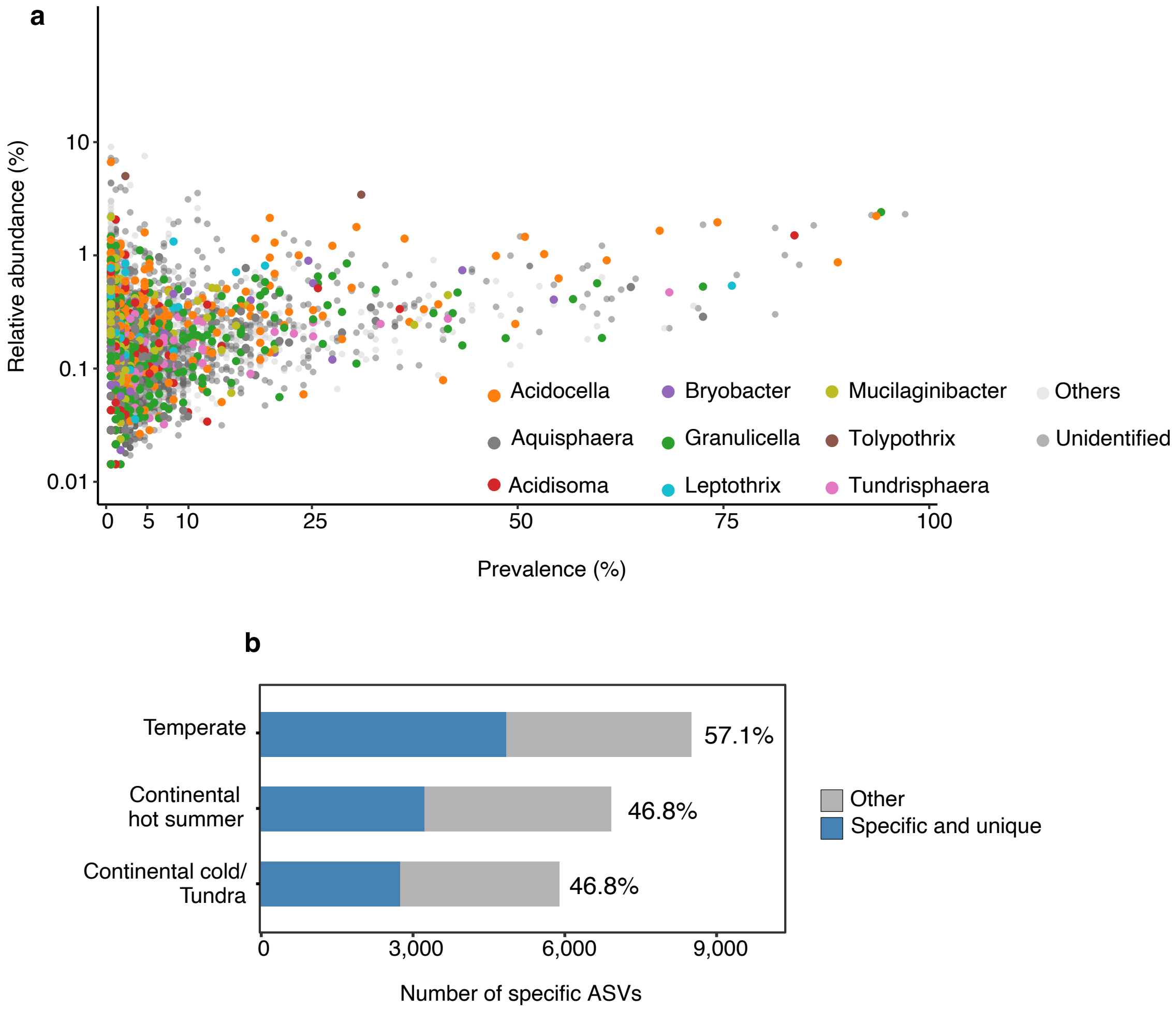

Fig. S16. Environmental drivers of bacterial turnover in SDPs. **a**, Reduction in deviance explained between the full Generalised Dissimilarity model (GDM) and the null Generalised Dissimilarity model (environmental variable omitted), *i.e.*, an indicator of the proportion of deviance attributed to that variable. Variables tested were geographical distance (geographic), minimum annual temperature (tmmn), annual precipitation (precipitation), snow water equivalent (swe), elevation, and pH. **b**, Partial GDM regression fits (Model-fitted-I-splines) for variables significantly associated with bacterial community species turnover. All variables have been normalised. The shape of each function provides an indication of how the rate of compositional turnover varies along the environmental gradient. Dashed lines are non-significant ($P > 0.05$).

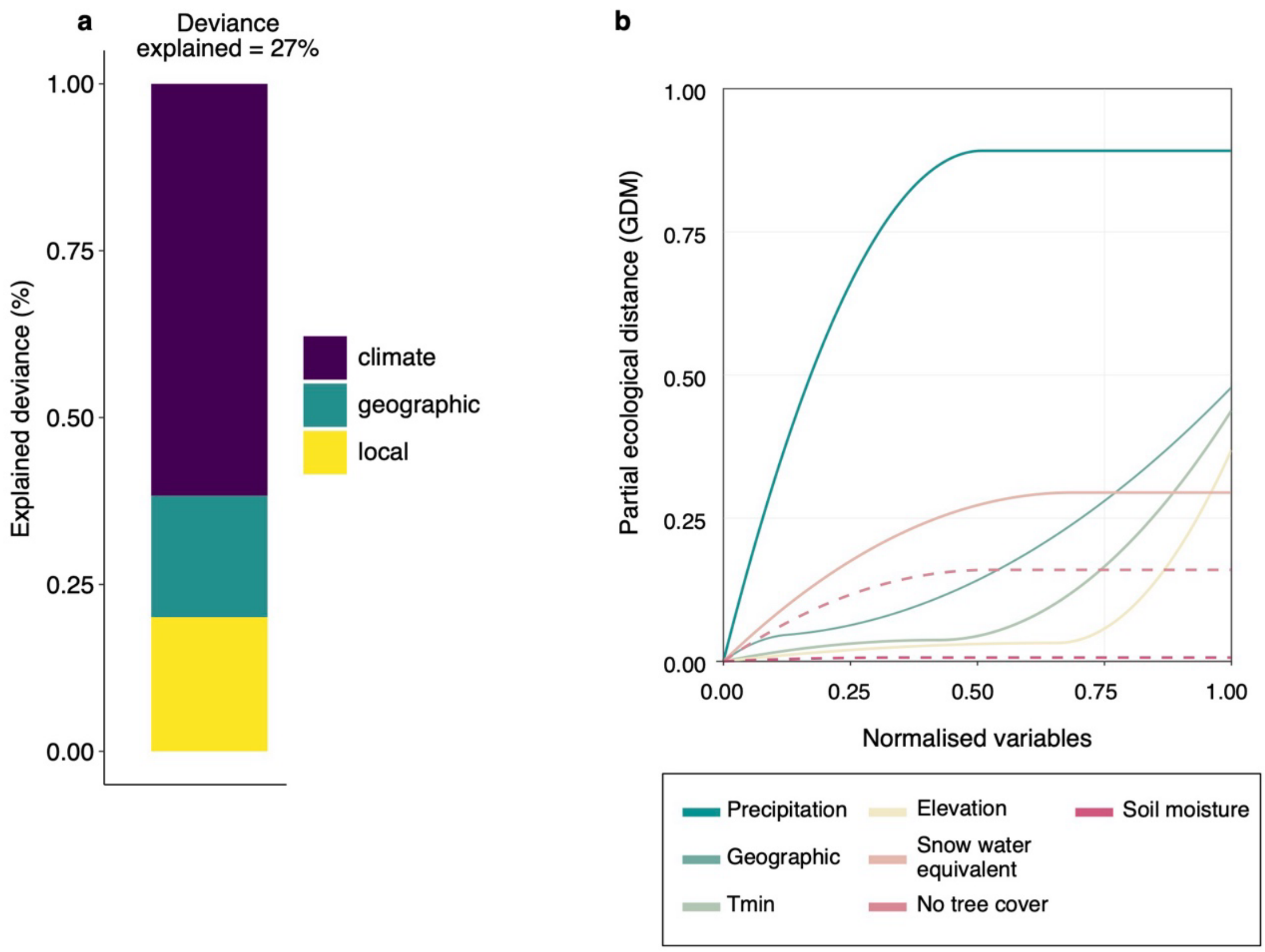

Fig. S17. Linkages between deterministic and stochastic processes and main environmental variables. Modified Mantel correlation between environmental variables and ecological processes. The grey dashed line indicates adjusted two-sided $P < 0.05$ (log).

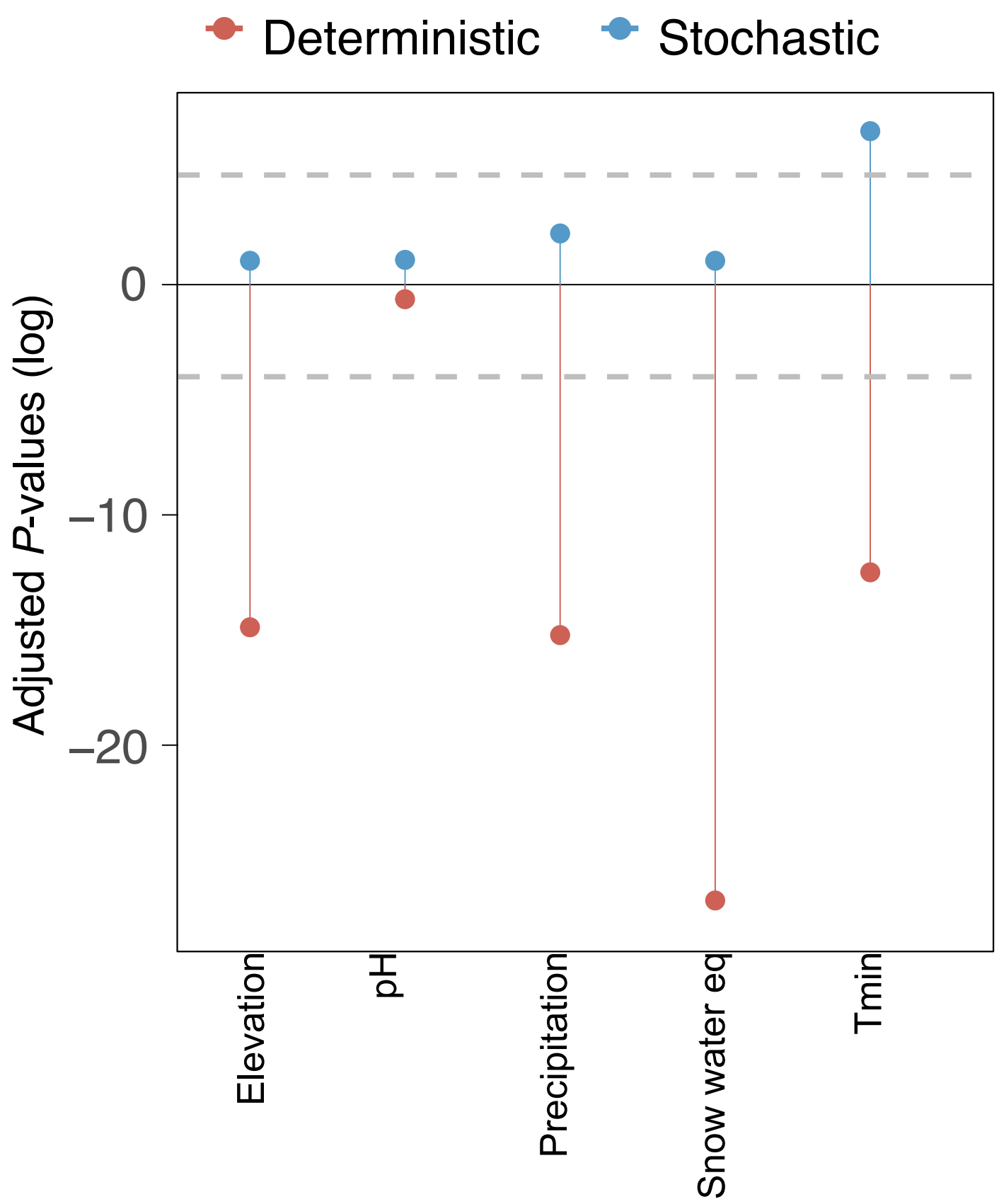

Fig. S18. Extracellular enzyme activities in SDPs. ß-1,4-glucosidase (BG, **a**), ß-1,4-N-acetylglucosaminidase (NAG, **b**), leucine aminopeptidase (LAP, **c**), and acid phosphatase (AP, **d**) enzyme activities in SDPs. Enzymatic patterns across bioclimatic regions are shown in boxplots. Boxplots indicate the median (centre line), first and third quartiles (lower and upper box edges) and 1.5x the interquartile range (IQR) (box whiskers). Letters indicate significant differences between bioclimatic regions (ANOVAs).

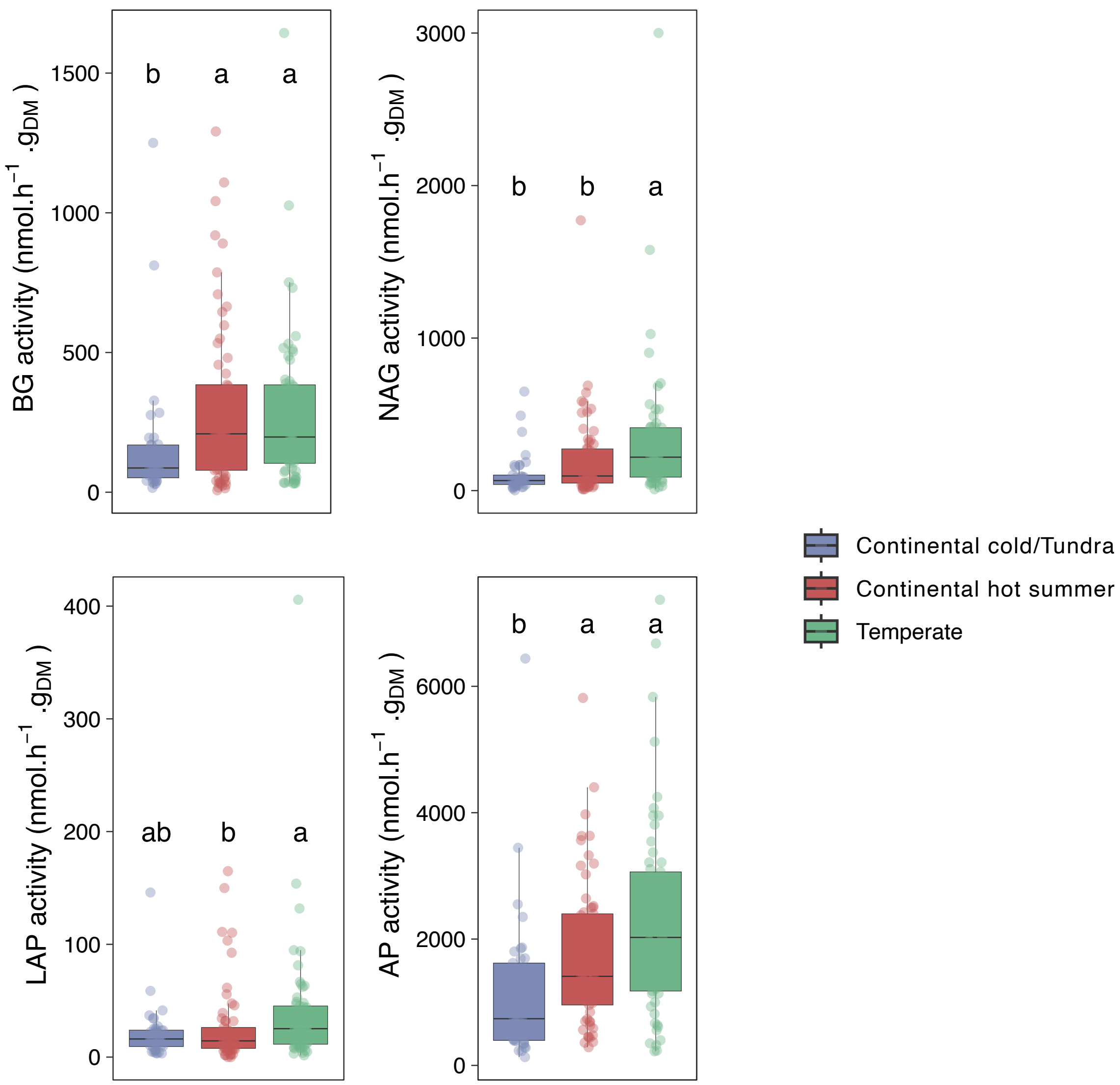

Fig. S19. Main drivers of enzyme activities in SDPs. Importance of main environmental variables in explaining the activity of the four enzymes (BG, NAG, LAP, AP) based on Spearman correlation coefficients (ρ). Bars represent the absolute value of the Spearman correlation coefficient (ρ) for each variable, with significant correlations indicated by asterisks (* $P < 0.05$, ** $P < 0.01$, *** $P < 0.001$). Variables are minimum annual temperature (tmin), precipitation (pr), percentage of non-tree cover (non-tree cover), soil moisture, pH (ph), gross primary productivity (gpp) and snow water equivalent (swe). The intensity of the red colour highlights the strength of the correlation.

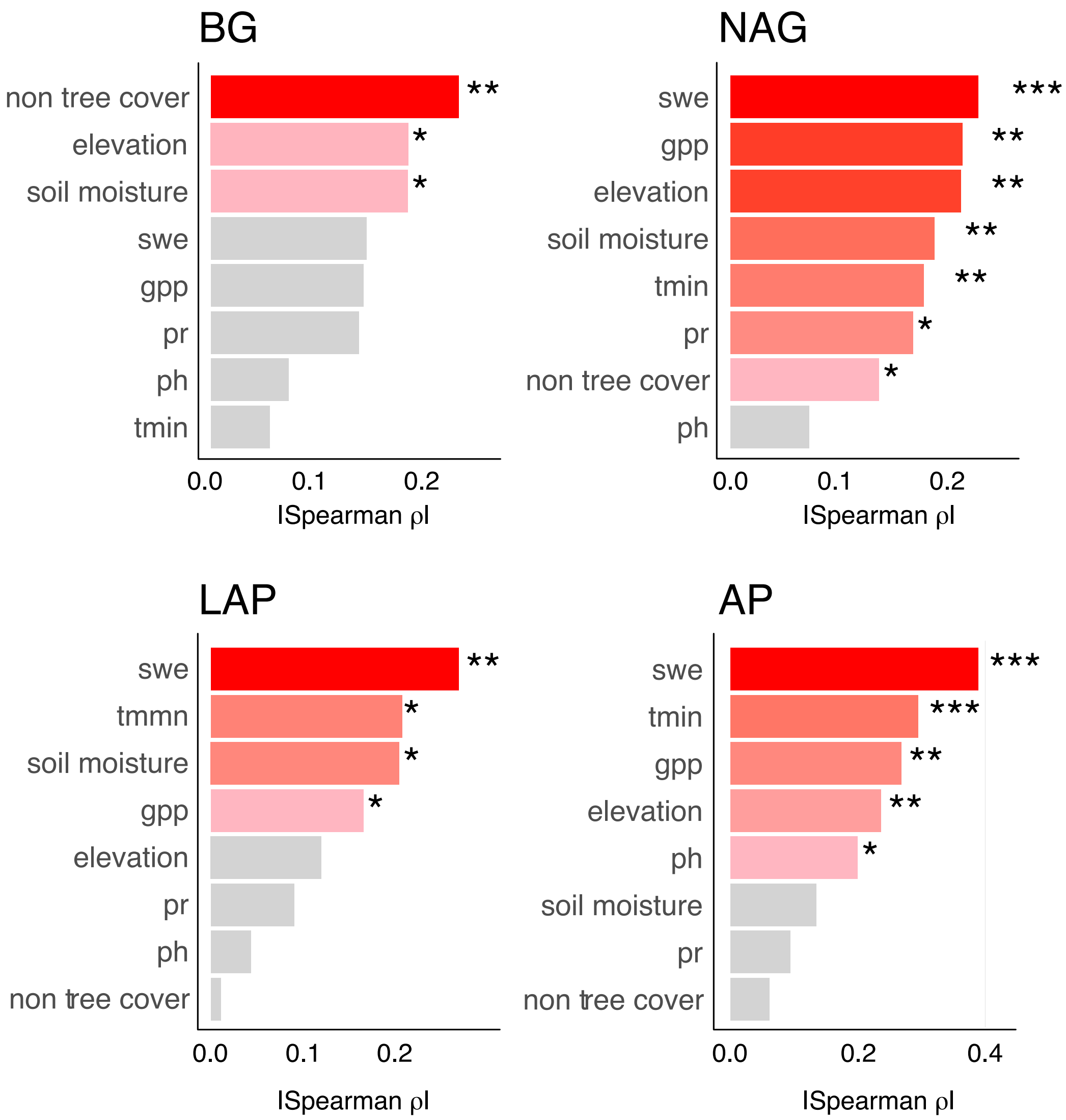

Fig. S20. Range of key environmental variables across SDPs. Distributions of key environmental and geographic variables across sampled SDPs. Each histogram illustrates the frequency of observed values for the respective variable for the sampling period.

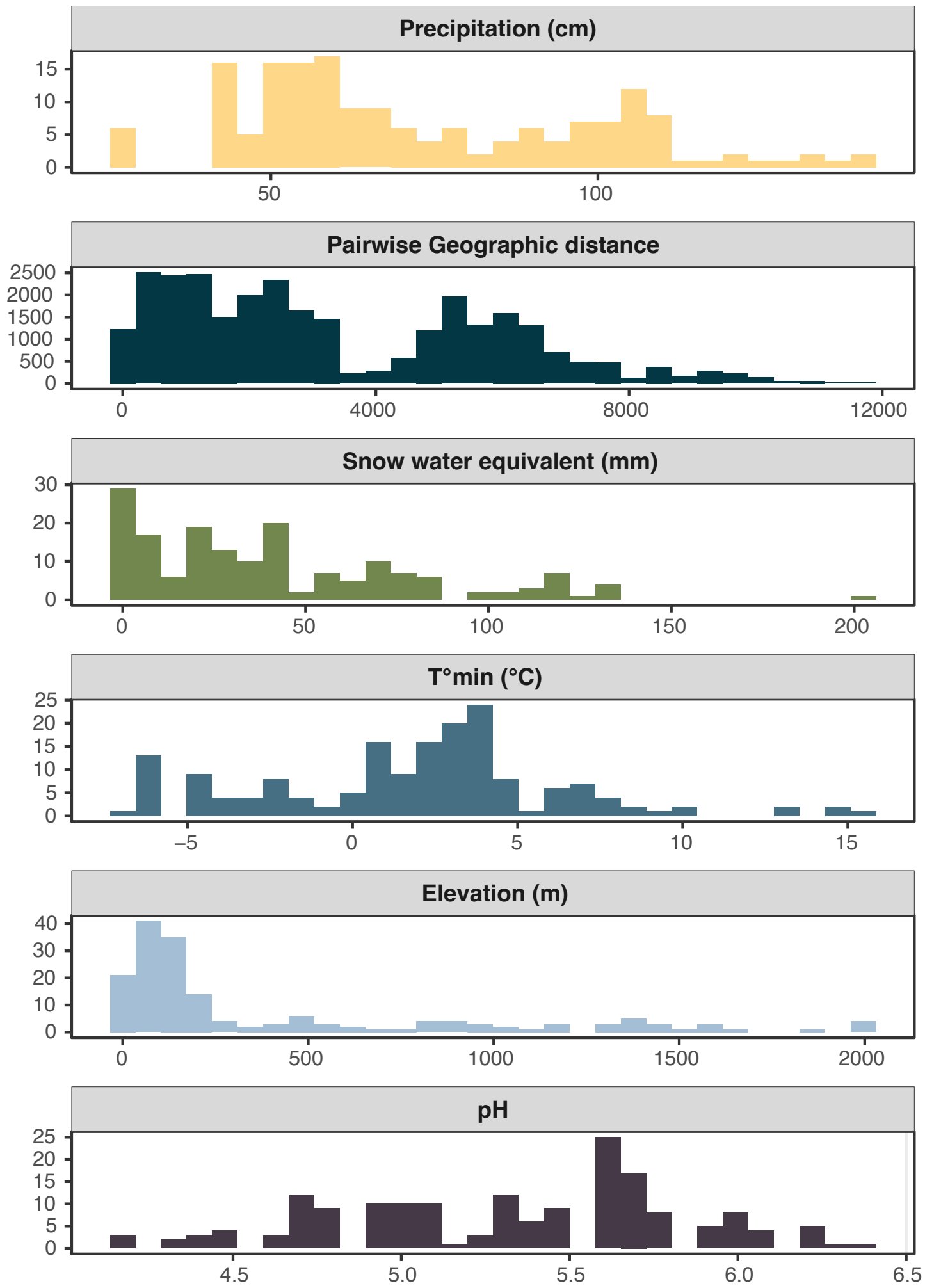

Fig. S21. Structural equation modelling (SEM) depicting the linkages between environmental variables, bacterial features (community composition, ASV richness and cell density), and enzyme activities. Bacterial community composition is represented by a latent variable defined by site scores on the PCoA axes (see Fig. 3). Enzyme multifunctionality is also represented by a latent variable, defined by each enzyme activity (see Methods). Grey lines indicate nonsignificant paths. The amount of variance explained (R^2) for each latent response variable are given on their respective box. Model fit was very good, with X^2 = 12.89, df = 19, *P* = 0.84; CFI = 1, RMSEA = 0, SRMR = 0.037. This model was built according to our *a priori* SEM model (Fig. S5, Table S4).

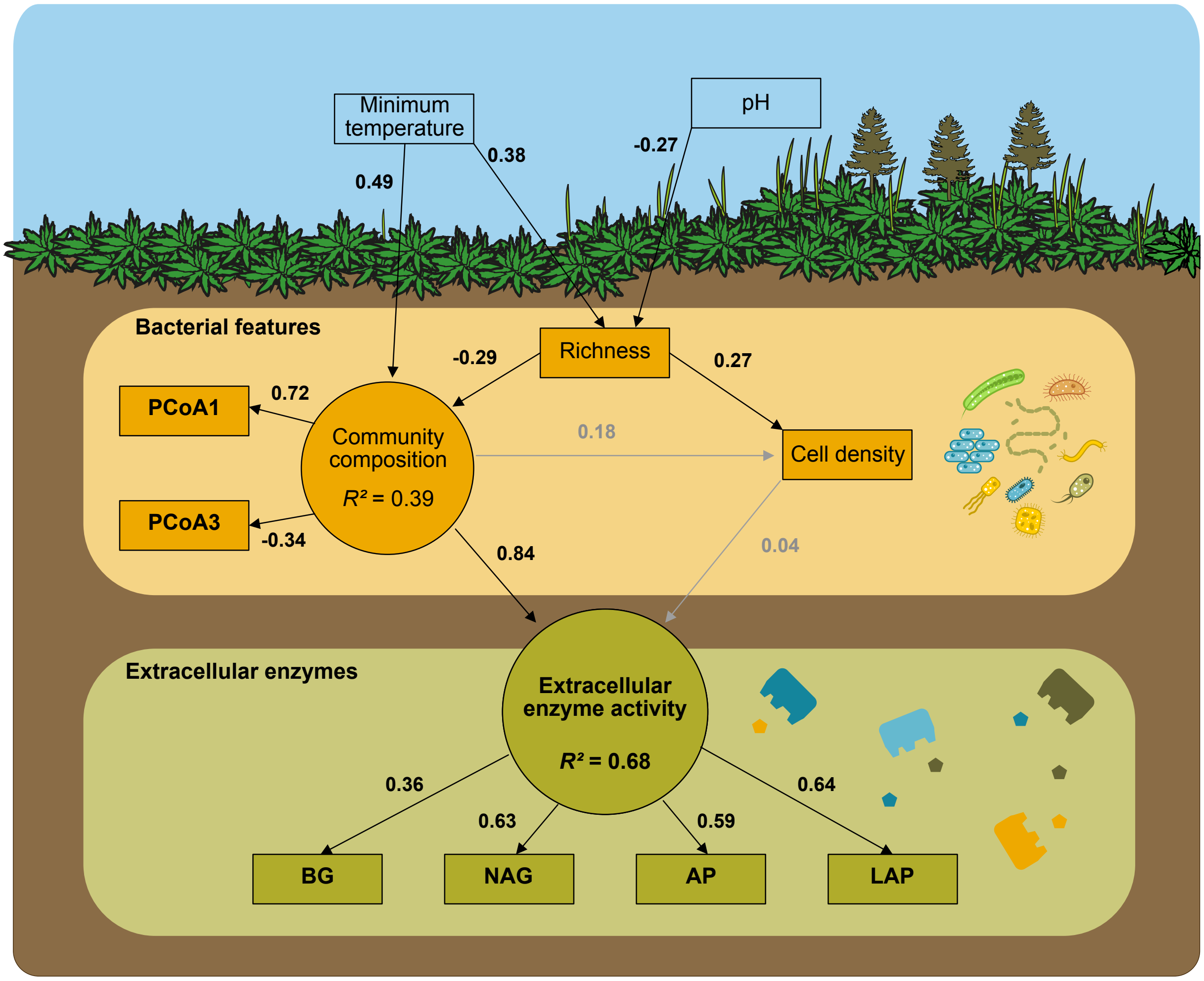

Fig. S22. Linkages between ASVs and enzyme activities. a, Taxonomic assignment and distribution of bacterial ASVs related to enzyme activities (in total 123 ASVs). Heatmap showing the strength of link between ASVs' relative abundance and the enzymatic rate (ANOVA β-coefficient, after FDR correction). **b**, Percentage of ASVs and Reads associated with enzyme activities. **c**, Number of ASVs per bacterial class positively (in green) or negative (in red) associated with enzyme activities.

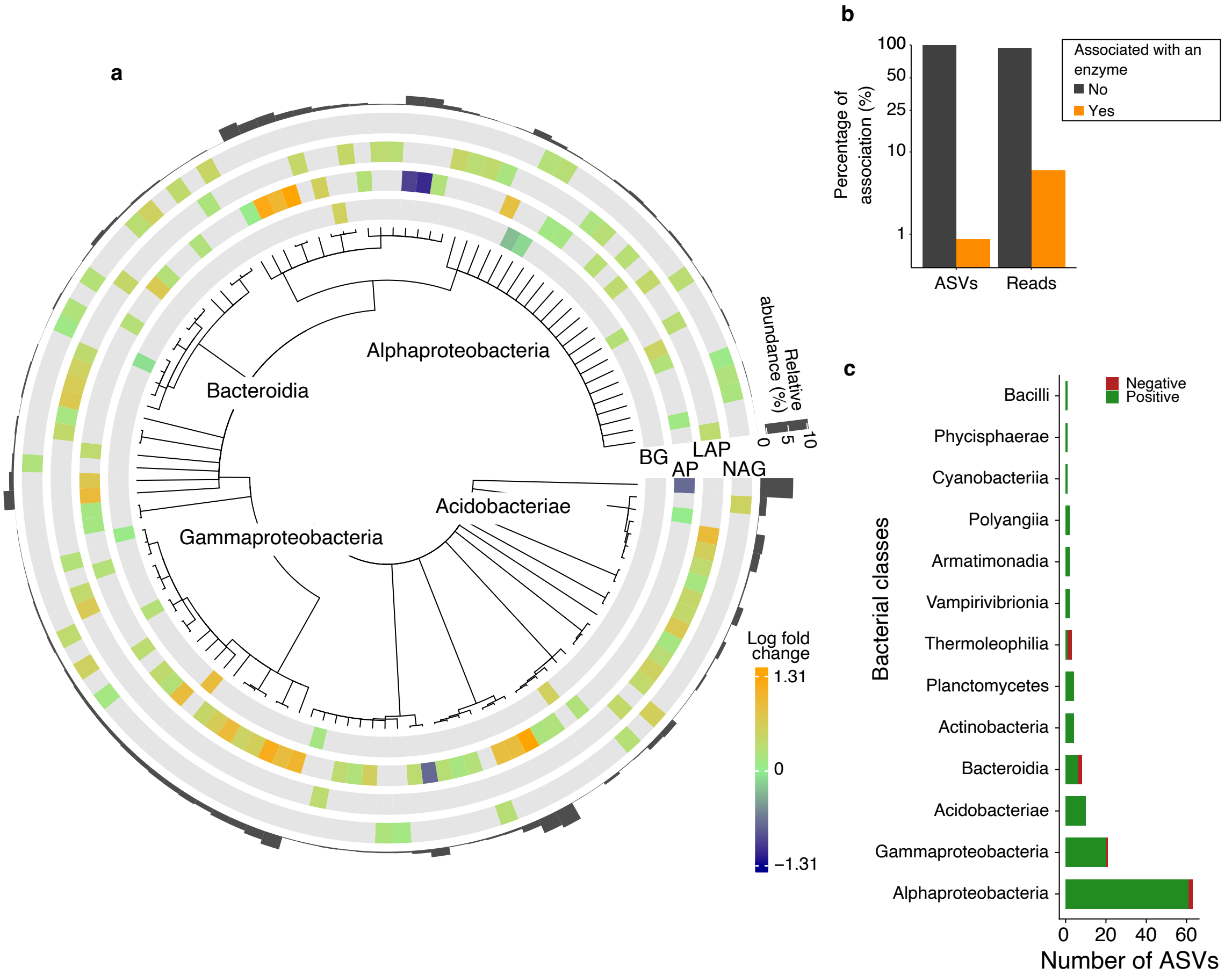

Fig. S23. Incubation temperature effect on enzyme activities. Enzyme activities in the reciprocal transplant experiment were incubated at the temperature of the receptor site as well as of the other donor sites (8, 16 and 20°C, see Methods for details). **a**, ß-1,4-glucosidase (BG), **b**, ß-1,4-N-acetylglucosaminidase (NAG), **c**, acid phosphatase (AP), and **d,** leucine aminopeptidase (LAP) enzyme activities. Enzymatic activities are compared across bioclimatic zones and split according to their incubation temperature. Boxplots indicate the median (centre line), first and third quartiles (lower and upper box edges) and 1.5x the interquartile range (IQR) (box whiskers). Letters indicate significant differences between treatments (bioclimatic zone, $P < 0.05$, ANOVAs; temperature x bioclimatic zone, $P < 0.05$ for all enzymes, ANOVAs).

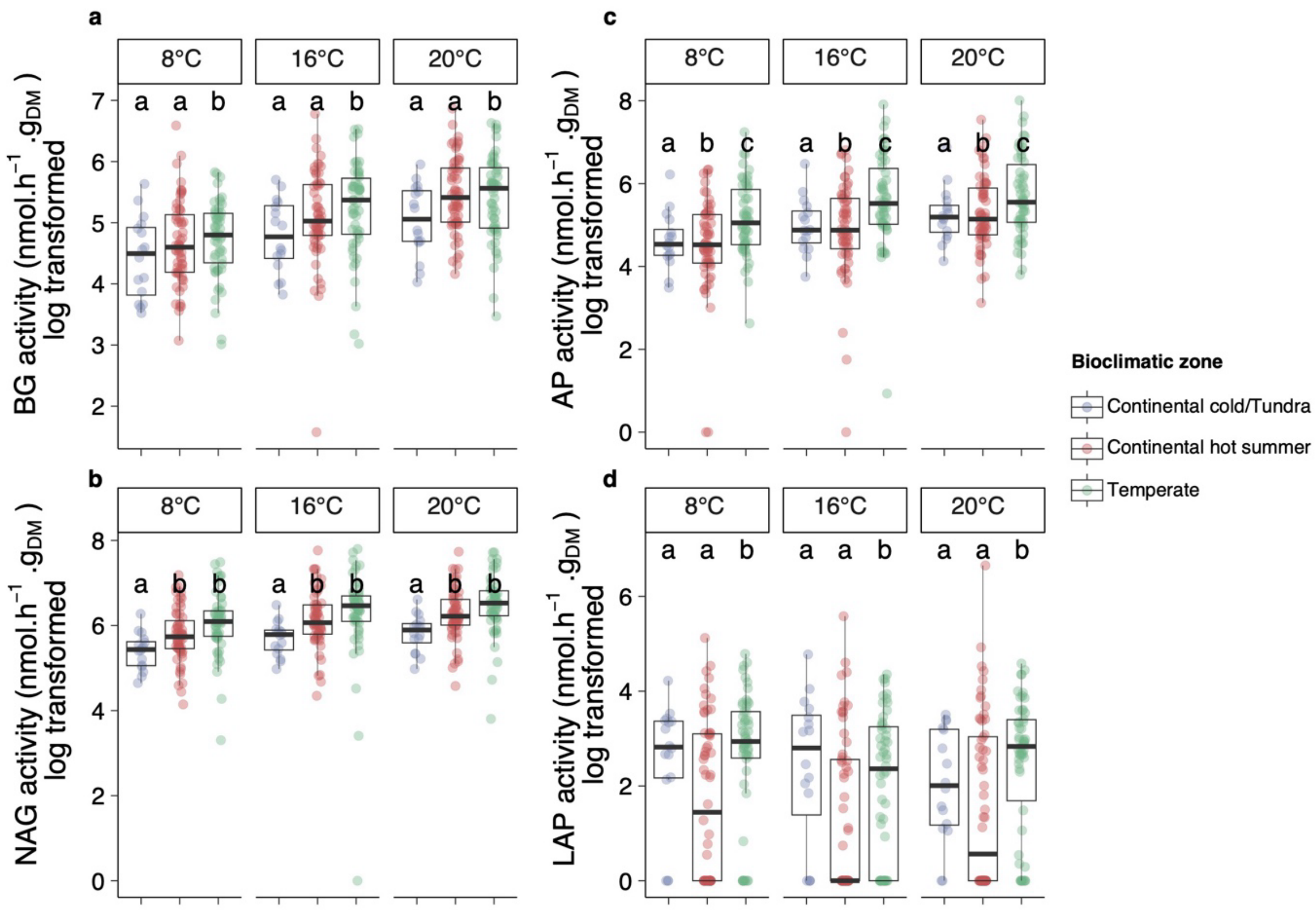

Supplement: Supplementary file 2 [file GCB-32-e71099-s007.pdf]
