# Supplementary material for: Tipping Points in Bacterial Richness Amplify Extracellular Enzyme Activities in Northern Peatlands
Source: Glob Chang Biol. 2026 Sep 17;32(9):e71099. doi: 10.1111/gcb.71099 (PMC13586543; doi:10.1111/gcb.71099)
Supplement: Supplementary file 3 [file GCB-32-e71099-s004.docx]

**Table S4: Components of hypotheses represented by the structural equation model**

| **Path** | **Causal hypothesis** | **References** |
| --- | --- | --- |
| 1-2 | Climatic and local abiotic conditions act as environmental filters that shape bacterial diversity, community composition and cell density. For example, temperature, moisture, pH and nutrient availability can alter bacterial growth, survival and community assembly. | (Bahram et al., 2018; Fierer & Jackson, 2006) |
| 3 | Vegetation influences bacterial diversity, community composition and cell density by modifying resource availability and habitat conditions. For example, higher plant productivity can increase the supply of organic substrates through litter inputs and root exudates, thereby affecting bacterial communities. | (Bardgett & van der Putten, 2014; Wardle et al., 2004) |
| 4-5 | Climatic and abiotic conditions may directly regulate extracellular enzyme activities by altering microbial metabolism, substrate availability and enzyme stability, independently of changes in bacterial community properties. | (Fanin et al., 2022; Sinsabaugh et al., 2008) |
| 6 | Vegetation may directly stimulate extracellular enzyme activities through increased carbon inputs (e.g. root exudates and fresh litter), which enhance microbial activity and enzyme production (Dieleman et al., 2017; Robroek et al., 2016). | (Dieleman et al., 2017; Robroek et al., 2016) |
| 7 | Bacterial richness, community composition and cell density regulate extracellular enzyme activities by determining the abundance of enzyme-producing bacteria, the diversity of functional traits and the overall microbial biomass available for enzyme production. | (Schimel & Schaeffer, 2012) |

**References cited**

Bahram, M., Hildebrand, F., Forslund, S. K., Anderson, J. L., Soudzilovskaia, N. A., Bodegom, P. M., Bengtsson-Palme, J., Anslan, S., Coelho, L. P., Harend, H., Huerta-Cepas, J., Medema, M. H., Maltz, M. R., Mundra, S., Olsson, A., Pent, M., Põlme, S., Sunagawa, S., Ryberg, M., & Tedersoo, L. (2018). Structure and function of the global topsoil microbiome. *Nature*. https://doi.org/10.1038/s41586-018-0386-6

Bardgett, R. D., & van der Putten, W. H. (2014). Belowground biodiversity and ecosystem functioning. *Nature*, *515*(7528), 505–511. http://dx.doi.org/10.1038/nature13855

Dieleman, C. M., Branfireun, B. A., & Lindo, Z. (2017). Northern peatland carbon dynamics driven by plant growth form—the role of graminoids. *Plant and Soil*, *415*, 25–35. https://link.springer.com/article/10.1007/s11104-016-3099-3

Fanin, N., Mooshammer, M., Sauvadet, M., Meng, C., Alvarez, G., Bernard, L., Bertrand, I., Blagodatskaya, E., Bon, L., Fontaine, S., Niu, S., Lashermes, G., Maxwell, T. L., Weintraub, M. N., Wingate, L., Moorhead, D., & Nottingham, A. T. (2022). Soil enzymes in response to climate warming: Mechanisms and feedbacks. *Functional Ecology*, *36*(6), 1378–1395. https://doi.org/10.1111/1365-2435.14027

Fierer, N., & Jackson, R. B. (2006). The diversity and biogeography of soil bacterial communities. *Proceedings of the National Academy of Sciences of the United States of America*, *103*(3), 626–631. http://eutils.ncbi.nlm.nih.gov/entrez/eutils/elink.fcgi?dbfrom=pubmed&id=16407148&retmode=ref&cmd=prlinks

Robroek, B. J. M., Albrecht, R. J. H., Hamard, S., Pulgarin, A., Bragazza, L., Buttler, A., & Jassey, V. E. J. (2016). Peatland vascular plant functional types affect dissolved organic matter chemistry. *Plant and Soil*, *407*(1–2), 135–143. http://link.springer.com/10.1007/s11104-015-2710-3

Schimel, J. P., & Schaeffer, S. M. (2012). Microbial control over carbon cycling in soil. *Frontiers in Microbiology*, *3*, 348. http://journal.frontiersin.org/article/10.3389/fmicb.2012.00348/abstract

Sinsabaugh, R. L., Lauber, C. L., Weintraub, M. N., Ahmed, B., Allison, S. D., Crenshaw, C., Contosta, A. R., Cusack, D., Frey, S., Gallo, M. E., Gartner, T. B., Hobbie, S. E., Holland, K., Keeler, B. L., Powers, J. S., Stursova, M., Takacs‐Vesbach, C., Waldrop, M. P., Wallenstein, M. D., … Zeglin, L. H. (2008). Stoichiometry of soil enzyme activity at global scale. *Ecology Letters*, *11*(11), 1252–1264. https://doi.org/10.1111/j.1461-0248.2008.01245.x

Wardle, D. A., Bardgett, R. D., Klironomos, J. N., Setälä, H., van der Putten, W. H., & Wall, D. H. (2004). Ecological linkages between aboveground and belowground biota. *Science*, *304*(5677), 1629–1633. http://www.sciencemag.org/content/304/5677/1629.short
